# Comparative analysis reveals the modular functional structure of conjugative megaplasmid pTTS12 of *Pseudomonas putida* S12: A paradigm for transferable traits, plasmid stability, and inheritance?

**DOI:** 10.3389/fmicb.2022.1001472

**Published:** 2022-09-23

**Authors:** Hadiastri Kusumawardhani, Rohola Hosseini, Jo-Anne Verschoor, Johannes H. de Winde

**Affiliations:** ^1^Institute of Biology Leiden, Leiden University, Leiden, Netherlands; ^2^Department of Fundamental Microbiology, University of Lausanne, Lausanne, Switzerland

**Keywords:** *Pseudomonas putida*, genome sequence, solvent tolerance, megaplasmid, mobile genetic elements, comparative analysis

## Abstract

Originating from various environmental niches, large numbers of bacterial plasmids have been found carrying heavy metal and antibiotic resistance genes, degradation pathways and specific transporter genes for organic solvents or aromatic compounds. Such genes may constitute promising candidates for novel synthetic biology applications. Our systematic analysis of gene clusters encoded on megaplasmid pTTS12 from *Pseudomonas putida* S12 underscores that a large portion of its genes is involved in stress response to increase survival under harsh conditions like the presence of heavy metal and organic solvent. We investigated putative roles of genes encoded on pTTS12 and further elaborated on their roles in the establishment and maintenance under several stress conditions, specifically focusing on solvent tolerance in *P. putida* strains. The backbone of pTTS12 was found to be closely related to that of the carbapenem-resistance plasmid pOZ176, member of the IncP-2 incompatibility group, although the carbapenem resistance cassette is absent from pTTS12. Megaplasmid pTTS12 contains multiple transposon-flanked cassettes mediating resistance to various heavy metals such as tellurite, chromate (Tn*7*), and mercury (Tn*5053* and Tn*5563*). Additionally, pTTS12 also contains a P-type, Type IV secretion system (T4SS) supporting self-transfer to other *P. putida* strains. This study increases our understanding in the modular structure of pTTS12 as a member of IncP-2 plasmid family and several promising exchangeable gene clusters to construct robust microbial hosts for biotechnology applications.

## Introduction

Bacteria may use plasmids as autonomous, self-replicating elements driving horizontal transfer of genes (HGT) that confer resistance to otherwise detrimental conditions. As such, these extrachromosomal entities often confer advantageous characteristics for the host strain (Molina et al., 2011; Xiong et al., 2013; Cazares et al., 2020). The rapid spread of resistance genes through large conjugative plasmids is further facilitated by the presence of a variety of mobile genetic elements such as transposons, integrons and insertion sequences (IS’s) (Kholodii et al., 1993; Nojiri et al., 2004; Xiong et al., 2006; Hosseini et al., 2017). The spread of multidrug resistance (MDR) via mobile genetic elements has been the subject of investigation for a number of years (Partridge et al., 2018). Despite those efforts, the role and functioning of megaplasmids is still poorly understood. Recent studies highlighted the role of megaplasmids in the spread of MDR in the opportunistic pathogen *Pseudomonas aeruginosa* (Xiong et al., 2013; Cazares et al., 2020). Strains of its close relative, *Pseudomonas putida*, have been known to harbor large conjugational plasmids, conferring resistance to environmental threats (Ramos et al., 1995; Molina et al., 2011). Plasmids of the *Pseudomonas* family are classified by incompatibility groups, that exhibit various modes of compatibility and transferability (Dennis, 2005; Shintani et al., 2015). We have chosen to study the recently identified megaplasmid pTTS12 of *Pseudomonas putida* S12 in comparison with a number of other large plasmids of the *Pseudomonas* family, in order to elucidate key elements governing HGT, stability and essential functions.

*Pseudomonas putida* S12 is a gram-negative soil bacterium which was isolated to utilize styrene as a sole carbon source (Hartmans et al., 1990). This strain shows a remarkable tolerance toward non-metabolized organic solvents (e.g., toluene) (Kieboom et al., 1998a). Such high tolerance toward organic solvents presents a beneficial trait and advantage for bioproduction of aromatics and biofuel (Mukhopadhyay, 2015; Kusumawardhani et al., 2018). Due to its solvent tolerance and versatile metabolism, *P. putida* S12 excels as a microbial host for production of valuable chemicals (Wierckx et al., 2005; Verhoef et al., 2007, 2009; Koopman et al., 2010; Janardhan Garikipati and Peeples, 2015). Removal of organic solvent molecules from the bacterial cell membrane is essential and carried out by SrpABC, a resistance-nodulation-cell division (RND) family efflux pump (Kieboom et al., 1998a,b). Membrane compaction and the upregulation of chaperones, general stress responses, and TCA cycle-related enzymes support a further intrinsic solvent tolerance in *P. putida* S12 (Volkers et al., 2006, 2015; Wijte et al., 2011; Rühl et al., 2012).

We recently found through whole-genome sequencing that the genome of *P. putida* S12 consists of a 5.8 Mbp chromosome and a 583 kbp single-copy megaplasmid pTTS12 (Kuepper et al., 2015). Both the SrpABC RND-efflux pump and the styrene degradation pathway which are the major distinctive features of *P. putida* S12, are encoded on this megaplasmid. The genome of *P. putida* S12 contains a large number of several types of mobile elements, spread over both chromosome and megaplasmid pTTS12 (Wery et al., 2001; Volkers et al., 2010; Kuepper et al., 2015). Some of these insertion sequences were shown to be involved in the regulation and adaptation toward stress conditions, for example during the solvent stress (Sun and Dennis, 2009; Hosseini et al., 2017). Like *P. putida* S12, related solvent-tolerant *P. putida* DOT-T1E and *Pseudomonas taiwanensis* VLB120 have been shown to harbor megaplasmids of 121 and 312 kb, respectively (Panke et al., 1998; Rodríguez-Herva et al., 2007; Köhler et al., 2013). Indeed, those plasmids encode RND-type efflux pumps and biodegradative pathways for aromatic compounds, similar to *P. putida* S12. To further characterize pTTS12, we here performed a comparative analysis against a large number of other megaplasmids from Refseq and Nuccore databases. With this analysis, we aimed at identifying the origin of the modular structure of environmental-stress related gene clusters in pTTS12.

## Materials and methods

### Cultivation of *Pseudomonas putida*

Strains and plasmids used in this report were listed in Table 1. All *P. putida* strains were grown in Lysogeny Broth (LB) containing 10 g L^–1^ tryptone, 5 g L^–1^ yeast extract and 5 g L^–1^ sodium chloride at 30°C with 200 rpm shaking. *E. coli* strains were cultivated in LB at 37°C with 250 rpm shaking in a horizontal shaker (Innova 4330, New Brunswick Scientific). For solid cultivation, 1.5% (w/v) agar was added to LB. M9 minimal medium used in this report was supplemented with 2 mg L^–1^ MgSO_4_ and 0.2% w/v of citrate as sole carbon source (Hartmans et al., 1990). Bacterial growth was observed by optical density measurement at 600 nm (OD_600*nm*_) using a spectrophotometer (Ultrospec 2100 pro, Amersham Biosciences). Maximum growth rate and other parameters were calculated using growthcurver R-package ver.0.3.0 (Sprouffske and Wagner, 2016). Solvent tolerance analysis was performed by growing *P. putida* strains in LB starting from OD_600*nm*_ = 0.1 in Boston bottles with Mininert bottle caps. When required, potassium tellurite (6.75–200 mg L^–1^), indole (100 mg L^–1^), gentamicin (25 mg L^–1^), ampicillin (100 mg L^–1^), tetracycline (25 mg L^–1^), and kanamycin (50 mg L^–1^) were added to the media.

**TABLE 1:** Bacterial strains and plasmids used in this study.

Strain/plasmid	Relevant characteristics	References
*E. coli* DH5α	*sup* E44, Δ*lacU*169 (Φ*lacZ*ΔM15), *recA*1, *endA*1, *hsdR*17, *thi*-1, *gyrA*96, *relA*1	Bethesda Research Laboratories (1986)
*E. coli* DH5αλpir	*sup* E44, Δ*lacU*169 (Φ*lacZ*ΔM15), *recA*1, *endA*1, *hsdR*17, *thi*-1, *gyrA*96, *relA*1, λpir phage lysogen	Herrero et al. (1990)
*E. coli* HB101	*recA pro leu hsdR* Sm*^R^*	Boyer and Roulland-dussoix (1969)
*E. coli* XL-1 Blue	endA1 gyrA96(nalR) thi-1 recA1 relA1 lac glnV44 F’[:Tn*10* proAB + lacIq Δ(lacZ)M15] hsdR17(rK- mK +) Tc*^R^*	Stratagene
*E. coli* MV1190	delta(lac-proAB) thi supE44 delta(sr1-recA)306:Tn*10* [F’:traD36 proAB lacIq delta(lacZ)M15] Tc*^R^*	ATCC
*P. putida* KT2440	Derived from wild-type *P. putida* mt-2, ΔpWW0	Bagdasarian et al. (1981)
*P. putida* S12	Wild-type *P. putida* S12 (ATCC 700801), harboring megaplasmid pTTS12	Hartmans et al. (1990)
*P. putida* S12.1	*P. putida* S12; Km*^R^* on pTTS12	This paper
*P. putida* KT-BG35	*P. putida* KT2440; Gm*^R^*, *msfgfp*:Tn*7*	This paper
*P. putida* KTpS12	*P. putida* KT2440, Gm*^R^*, *msfgfp*:Tn*7*, pTTS12, Km*^R^*	This paper
pRK2013	RK2-Tra^+^, RK2-Mob^+^, Km*^R^*, *ori* ColE1	Figurski and Helinski (1979)
pTTS12	A 583 kbp megaplasmid of *P. putida* S12	Kuepper et al. (2015)
pTnS-1	Ap*^R^*, *ori* R6K, TnSABCD operon	Choi et al. (2005)
pBG35	Km*^R^*, Gm*^R^*, *ori* R6K, pBG-derived	Zobel et al. (2015)
pEMG	Km*^R^*, Ap*^R^*, *ori* R6K, *lacZ*α MCS flanked by two I-SceI sites	Martínez-García and de Lorenzo (2011)
pEMG-28750	pEMG plasmid for constructing pTTS12 ΔRPPX_28750	This paper
pSW-2	Gm*^R^* ori RK2 *xylS* Pm→I-SceI	Martínez-García and de Lorenzo (2011)

### DNA methods

All PCR reactions were performed using Phire polymerase (Thermo Fischer) according to the manufacturer’s manual. All primers are listed in Table 2 and were obtained from Sigma-Aldrich. PCR reactions were visualized and analyzed by gel electrophoresis on 1% (w/v) TBE agarose gels containing 5 mg L^–1^ ethidium bromide in an electric field (110 V, 0.5× TBE running buffer).

**TABLE 2:** Primers used in this study.

Primer	Sequence	Target
glmS_Fw	AGTCAGAGTTACGGAATTGTAGG	*P. putida* KT2440 (Tn*7* site)
glmS_Rv	GTCGAGAAAATTGCCGAGCT	*P. putida* KT2440 (Tn*7* site)
53,496_Fw	ACTTCGACCAATGCCCCATT	*P. putida* S12 pTTS12
56,596_Rv	GGACACCCTCATCCTTAGCG	*P. putida* S12 pTTS12
200,497_Fw	GTGTATCGAAGGGCCTCCAC	*P. putida* S12 pTTS12
203,602_Rv	TCGACGATGCAGACAGATCG	*P. putida* S12 pTTS12
286,448_Fw	AACACCGAAGATGGGGCTTT	*P. putida* S12 pTTS12
289,462_Rv	GCAGGTCGACAAGCAAGTTG	*P. putida* S12 pTTS12
4,963,661_Fw	ATCACCCAGCTGAGCCATTC	*P. putida* S12 chromosome
4,966,726_Rv	CTGCCGGATAACAAAGCAGC	*P. putida* S12 chromosome
90285_Fw	TTT**TCTAGA**TGCTGAGCAGTTCCTTCAGG	Construction of pEMG-TS for Km*^R^* marker in pTTS12, with *Xba*I restriction site
90825_Rv	TTT**CCCGGG**AGGAAGGGAAGCAAACTCCG	Construction of pEMG-TS for Km*^R^* marker in pTTS12, with *Xma*I restriction site
TS1_28750_Fw	AATCT**GAATTC**GGACGTATTGGGCTTCAATG	Construction of pEMG-Δ28750 for deleting the putative relaxase, with *Eco*RI restriction site
TS1_28750_Rv	GAAAGCCTGTCTGCACATGGCTATCGACTCA TCATTCACCG	Construction of pEMG-Δ28750 for deleting the putative relaxase
TS2_28750_Fw	CGGTGAATGATGAGTCGATCATGTGCAGACA GGCTTTC	Construction of pEMG-Δ28750 for deleting the putative relaxase
TS2_28750_Rv	AACCC**GGATCC**GTTCGACAGCCGCTATTTC	Construction of pEMG-Δ28750 for deleting the putative relaxase, with *Bam*HI restriction site
test_28750_Fw	CCTGATGCACGATTTACCG	Confirming the deletion of the putative relaxase (ΔRPPX_28750)
test_28750_Fw	CTACCTGCCGGTACACATT	Confirming the deletion of the putative relaxase (ΔRPPX_28750)

### Megaplasmid pTTS12 transfer into *Pseudomonas putida* KT2440 and *Escherichia coli* strains

Gentamicin resistance and GFP containing cassette were incorporated into *P. putida* KT2440 chromosome at the Tn*7* site using pBG35 plasmid resulting in the strain *P. putida* KT-BG35 as previously described (Zobel et al., 2015). Correct integration of this construct was verified by observing the gentamicin resistance, GFP expression and colony PCR (glmS_Fw and glmS_Rv primers), followed by Sanger sequencing of the upstream region of *glmS* locus. The resulting strain is free of pTnS-1 plasmid and the helper plasmid pRK2013 by observing the absence of Ampicillin and Kanamycin resistance, respectively.

Kanamycin resistance gene was introduced into the megaplasmid pTTS12 by integrating plasmid pEMG using homologous recombination resulting in *P. putida* S12.1 as previously described (Martínez-García and de Lorenzo, 2011). A single homologous recombination site was obtained by PCR with 90285_Fw and 90825_Rv primer pair and this fragment was used to construct pEMG-TS plasmid. Correct integration into *P. putida* S12 was verified by observing kanamycin resistance and colony PCR using primers 4,963,661_Fw-4,966,726_Rv.

The transfer of pTTS12 into *P. putida* KT-BG35, *E. coli* XL1-Blue or *E. coli* MV1190 were performed by biparental mating between *P. putida* S12.1 and the recipient strain on LB agar. Both donor and recipient strains were grown overnight on LB agar at 30°C for *P. putida* strains and 37°C for *E. coli* strains. The colonies were resuspended in liquid LB media and diluted to about 2 × 10^8^ organisms per ml. A donor and a recipient strain suspension (100 μl) were mixed and collected on a cellulose-acetate filter (Sartorius; 0.45-μm pore size; 25-mm diameter) which was incubated for 24 h at 30°C on LB agar. Cells were resuspended in 1× Phosphate-buffered saline (PBS) solution by vigorous agitation and plated at appropriate dilutions on LB agar containing Gentamicin or Tetracycline and Kanamycin to select for for transconjugants. Plasmid transfer rate was determined by comparing the event of successful plasmid transconjugant with the colony formation unit (cfu) of the recipient strain (*P. putida* KT-BG35, *E. coli* XL1-Blue or *E. coli* MV1190) after biparental mating (Gm*^R^*/Tc*^R^*).

The correct transconjugants were selected using LB agar supplemented with gentamicin and kanamycin for *P. putida* KT-BG35 or with tetracycline and kanamycin for *E. coli* strains. Additionally, transconjugants were verified using colony PCR (53,496_Fw-56,596_Rv, 200,497_Fw-203,602_Rv, and 286,448_Fw-289,462_Rv). Plasmid stability was determined by calculating the event of megaplasmid loss in *P. putida* S12 and *P. putida* KTpS12 grown in liquid media without supplementation of kanamycin as the selective pressure for pTTS12. Plasmid pSW-2 was used as a control for megaplasmid loss events (Martínez-García and de Lorenzo, 2011). *P. putida* S12 ΔpTTS12 (Kusumawardhani et al., 2020) was used as a control strain of plasmid-less *P. putida* S12.

### pTTS12 sequence and comparative analyses

The pTTS12 sequence used in this analysis is the curated version of CP009975, in which we removed extra ISS12 mobile elements according to previous analysis (Hosseini et al., 2017) and corrected the disrupted genes. For this analysis, pTTS12 232 putative operons on pTTS12 were defined as sets of genes in close proximity which have the same direction in forward or reverse strand with less than 20 bps distance. The operons were clustered together based on the function of genes encoded within these clusters (Supplementary Table 1). Transposases were clustered together with the resistance genes if such transposase is known to carry the cassette.

All plasmid sequences were downloaded from the NCBI database, 22389 plasmid sequences from the Refseq database (retrieved 8 March 2020) and complemented with several plasmid sequences from NUCORE that were omitted from Refseq. The CGviewer (Stothard and Wishart, 2005) was used to generate circular plots of entire pTTS12 with standard settings and MultiGeneBlast tool (Medema et al., 2013) was used to generate synteny plots of specific regions and operons. Further basic alignments and visualization of alignments were performed using Geneious software (BioMatters), and selected sequences were aligned using MAFFT (Katoh and Standley, 2013) for DNA sequences or MUSCLE (Edgar, 2004) for protein sequences applying default settings in all cases.

## Results

### Comparative analysis of megaplasmid pTTS12

**FIGURE 1:**
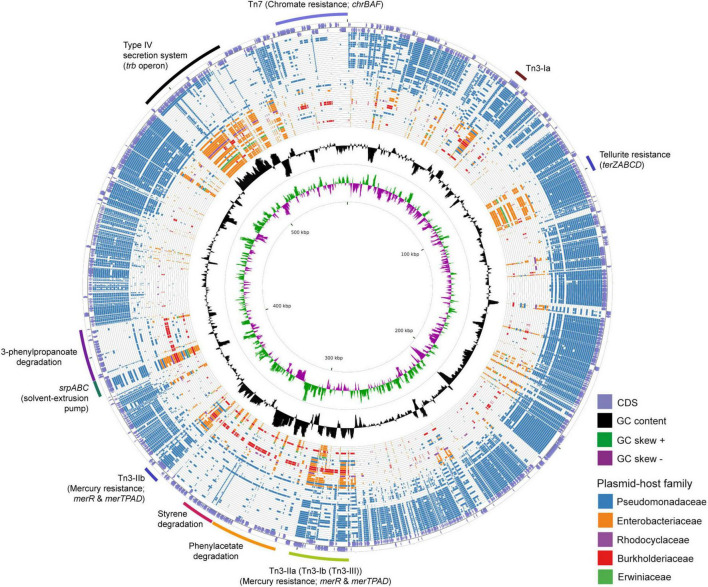
Circular plot of the top 50 plasmids with the highest identity scores to pTTS12. pTTS12 coding sequences (CDS) were aligned to 28915 other plasmids available at NCBI Refseq and Nuccore databases. The outer light purple ring represents the CDS of plus and minus strand of pTTS12. In the inner ring, the GC content is represented in black and the positive and negative GC skew are represented in green and purple respectively. The plasmids are ordered based on their similarity and coverage to pTTS12 CDS from outermost to innermost of the plot with each ring representing a single plasmid as listed in Table 3. In case no homologous protein was identified for a pTTS12 counterpart, that space on the circle was left blank. Ring colors represent different plasmid-host families; Pseudomonadaceae (blue), Enterobacteriaceae (orange), Rhodocyclaceae (purple), Burkholderiaceae (red), and Erwiniaceae (green). The position of the gene clusters of interest are annotated in the figure. An extended circular plot of the top 500 plasmids with highest identity scores to pTTS12 are shown in Supplementary Figure 1.

The 583 Kbps megaplasmid pTTS12 (GenBank accession no. CP009975) is a single-copy plasmid encoded in *P. putida* S12 (Kuepper et al., 2015). pTTS12 encodes 629 genes of which 583 are single copy and 15 genes are duplicated at least once. For this paper, annotations for pTTS12 were further extended (Supplementary Table 1) and the overall sequence was corrected based on our previous observations of an additional seven ISS12 mobile elements (Hosseini et al., 2017). Comparative analysis was performed with 28915 plasmids larger than 2 kb in length, acquired from the Refseq database^1^ combined with additional sequences from the Nuccore database.^2^ The top 50 plasmids encoding homologues of pTTS12 proteins are visualized in a circular plot using CGView (Stothard and Wishart, 2005) as shown on Figure 1 and listed in Table 3. The extended circular plot and list of the top 500 plasmids encoding homologues of pTTS12 can be found in Supplementary Figure 1 and Supplementary Table 2, respectively.

**TABLE 3:** List of 50 plasmids with the highest similarity scores to pTTS12.

Rank	Strain	Plasmid	Taxid	Length (bp)	Relative similarity	Inc group
	*Pseudomonas putida* S12	pTTS12	1215087	583900	100.00%	IncP-2
1	*Pseudomonas aeruginosa* PA96	pOZ176	1457392	500839	72.54%	IncP-2 (Xiong et al., 2006)
2	*Pseudomonas aeruginosa* strain FFUP_PS_37	pJB37	287	464804	69.30%	IncP-2 (Botelho et al., 2017)
3	*Pseudomonas aeruginosa* strain AR_0356	unnamed2	287	438531	67.97%	Unknown
4	*Pseudomonas aeruginosa* strain AR441	unnamed3	287	438529	66.23%	Unknown
5	*Pseudomonas putida* strain SY153	pSY153-MDR	303	468170	64.70%	IncP-2 (Zhang et al., 2021)
6	*Pseudomonas aeruginosa* strain T2436	pBT2436	287	422811	64.69%	IncP-2 (Cazares et al., 2020)
7	*Pseudomonas koreensis* strain P19E3	p1	198620	467568	64.44%	Unknown
8	*Pseudomonas putida* strain 12969	p12969-DIM	303	409102	64.43%	IncP-2 (Sun et al., 2016)
9	*Pseudomonas aeruginosa* strain T2101	pBT2101	287	439744	64.43%	IncP-2 (Cazares et al., 2020)
10	*Pseudomonas aeruginosa* strain PA298	pBM908	287	395774	63.99%	IncP-2 (Zhang et al., 2021)
11	*Pseudomonas aeruginosa* isolate RW109	RW109	287	555265	63.75%	IncP-2 (Weiser et al., 2019)
12	*Pseudomonas aeruginosa* strain PA121617	pBM413	287	423017	63.20%	Unknown
13	*Pseudomonas aeruginosa* strain PABL048	pPABL048	287	414954	62.82%	Unknown
14	*Pseudomonas aeruginosa*	p727-IMP	287	430173	62.75%	IncP-2 (Zhang et al., 2021)
15	*Pseudomonas aeruginosa* strain AR439	unnamed2	287	437392	62.00%	Unknown
16	*Pseudomonas citronellolis* strain SJTE-3	pRBL16	53408	370338	61.12%	IncP-2 (pRBL16) (Jiang et al., 2020)
17	*Pseudomonas aeruginosa*	p12939-PER	287	496436	60.86%	IncP-2 (pRBL16) (Jiang et al., 2020)
18	*Pseudomonas aeruginosa*	pA681-IMP	287	397519	59.50%	IncP-2 (pRBL16) (Jiang et al., 2020)
19	*Pseudomonas aeruginosa*	pR31014-IMP	287	374000	55.58%	IncP-2 (Zhang et al., 2021)
20	*Pseudomonas taiwanensis* VLB120	pSTY	69328	321653	21.65%	Unknown
21	*Pseudomonas fluorescens* SBW25	pQBR103	216595	425094	20.48%	IncA/C-IncP-3 (Tett et al., 2007)
22	*Pseudomonas syringae pv. maculicola* str. ES4326	pPma4326F	629265	387260	20.16%	Unknown
23	*Pseudomonas putida* strain KF715	pKF715A	303	483376	16.63%	IncP−9 (Suenaga et al., 2017)
24	*Pseudomonas stutzeri* strain YC-YH1	pYCY1	316	225945	16.06%	Unknown
25	*Pseudomonas fluorescens* SBW25	pQBR57	216595	307330	14.45%	Unknown
26	*Pseudomonas aeruginosa* strain PA83	unnamed1	287	398087	14.42%	Unknown
27	*Pseudomonas aeruginosa* strain DN1	unnamed1	287	317349	14.24%	Unknown
28	*Pseudomonas monteilii* strain FDAARGOS_171	unnamed	76759	60588	13.85%	Unknown
29	*Salmonella enterica* strain 8025	p8025	28901	311280	11.99%	IncHI1 (Kubasova et al., 2016)
30	*Pseudomonas luteola* strain FDAARGOS_637	unnamed1	47886	585976	10.92%	Unknown
31	*Enterobacter hormaechei subsp. steigerwaltii* strain 34998	p34998	299766	239973	10.39%	IncA/C (Chavda et al., 2016)
32	*Enterobacter hormaechei* strain A1	pIncHI2-1502264	158836	309444	10.37%	IncHI2
33	*Leclercia adecarboxylata* strain Lec-476	pLec-476	83655	311758	10.06%	IncHI1 (Papousek et al., 2017)
34	*Enterobacter hormaechei subsp. hoffmannii* strain AR_0365	unnamed1	1812934	328871	9.98%	Unknown
35	*Azospira sp.* I09	pAZI09	1765049	397391	9.98%	Unknown
36	*Citrobacter freundii* strain SL151	unnamed1	546	229406	9.89%	Unknown
37	*Cupriavidus metallidurans* strain FDAARGOS_675	unnamed3	119219	2586495	9.54%	Unknown
38	*Cupriavidus metallidurans* CH34	megaplasmid	266264	2580084	9.53%	Unknown
39	*Enterobacter hormaechei subsp. hormaechei* strain 34983	p34983	301105	328905	9.41%	IncHI1A, IncHI1B (Chavda et al., 2016)
40	*Escherichia coli* strain CFSAN064035	pGMI17-003_1	562	310064	9.19%	Unknown
41	*Pseudomonas sp.* XWY-1	pXWY	2069256	394537	9.15%	Unknown
42	*Klebsiella oxytoca* strain CAV1374	pKPC_CAV1374	571	332956	9.12%	Unknown
43	*Pseudomonas veronii* 1YdBTEX2	pPVE	1295141	373858	9.06%	Unknown
44	*Pantoea sp.* PSNIH2	pPSP-75c	1484157	378808	9.04%	IncHI1 (Kubasova et al., 2016)
45	*Klebsiella michiganensis* strain AR375	unnamed2	1134687	340462	8.96%	Unknown
46	*Citrobacter freundii* complex sp. CFNIH9	pCFR-eb27	2077149	355789	8.82%	Unknown
47	*Ralstonia solanacearum* strain HA4-1	HA4-1MP	305	1947245	8.82%	Unknown
48	*Enterobacteriaceae bacterium* ENNIH1	pENT-1f0b	2066051	302640	8.78%	Unknown
49	*Leclercia sp.* LSNIH1	pLEC-1cb1	1920114	341250	8.74%	Unknown
50	*Leclercia sp.* LSNIH3	pLEC-7c0d	1920116	330021	8.74%	Unknown

pTTS12 coding sequences (CDS) were aligned to other megaplasmids available at NCBI databases. The relative similarity score was calculated by dividing total scores for each plasmid by total score obtained for pTTS12 itself.

#### pTTS12 is highly similar with the carbapenem-resistance plasmid pOZ176 from *Pseudomonas aeruginosa* PA96

The majority of plasmids highly similar to pTTS12 were found in *Pseudomonas* genera (Table 3). The relative identity scores presented in Table 3 were calculated as a percentage of total scores of each plasmid divided by the total identity score obtained for pTTS12 itself. The plasmid most similar to pTTS12 is pOZ176 from *P. aeruginosa* PA96 which was previously categorized as a member of incompatibility group P-2 (IncP-2). pOZ176 and pTTS12 share more than 71% similarity of their encoded genes indicating that these plasmids share a similar IncP-2 backbone. Whereas pOZ176 encodes a carbapenem-resistance gene cluster typical for several pathogenic *P. aeruginosa* strains (Xiong et al., 2013), pTTS12 of *P. putida* S12 does not share this feature. Instead, pTTS12 contains gene clusters for styrene degradation, phenylpropionic acid degradation, and solvent efflux pump (*srpABC*), which are absent in pOZ176.

#### Other features of pTTS12 are widely distributed among other soil bacteria and enterobacteria

Between 8 to 12% of encoded proteins from pTTS12 can also be found in other genera than *Pseudomonas* (Table 3). Several plasmids from *Salmonella*, *Enterobacter*, *Citrobacter*, *Leclersia*, *Klebsiella*, *Pantoea*, *Polaromonas*, and *Cupriavidus* species shared homologous proteins involved in the T4SS conjugation, replication machinery, and plasmid maintenance. Like other IncP-2 plasmids, pTTS12 contains multiple transposable elements, particularly Tn*3* mobile elements (Figure 1). This mobile element encodes for heavy metal resistance genes that are predominantly present in IncP-2 plasmids. Additionally, pTTS12 carries a unique Tn*7*-element consisting of several chromate resistance genes.

### Replication and maintenance of pTTS12

Megaplasmid pTTS12 contains two replication and partitioning machineries. In a recent report, Shintani and colleagues argued that pOZ176 encodes two replication initiation protein (RIP); the primary RIP gene (pOZ176_183) and the auxiliary RIP gene (pOZ176_301) (Shintani et al., 2022). In pTTS12, we found that RPPX_27060 putatively encodes the primary RIP that shares 99% identity, 100% coverage with the primary RepA protein of pOZ176 (pOZ176_301) (Supplementary Figure 4A). In addition, the putative genes around this region show synteny and high similarity between pTTS12 and pOZ176. It must be noted, however, that there are two mobile elements present in pTTS12, disrupting the homologous regions with pOZ176_182 and pOZ176_186 (Supplementary Figure 4A).

Meanwhile, RPPX_28775 putatively encodes for the auxiliary RIP (285 amino acids) that shares 86.48% identity, 98% coverage with the auxiliary RepA protein of pOZ176 (pOZ176_301) (Supplementary Figure 4B). Downstream of RPPX_28775, pTTS12 contains three copies of direct repeats TCGTGCTATCAGGAGTA (N_7_) TCGTGCTATC**G**GGAGTA (N_6_) TCGTGCTATCAGGAGTA with one base mismatch compared to the direct repeats found in pOZ176 (Xiong et al., 2013). These direct repeats may constitute putative iterons at the origin of replication site. RPPX_28770 and RPPX_28765 loci, downstream of RPPX_28775 and iterons, putatively encodes for ParA-ATPase (91.04% identity, 100% coverage) and ParB (85.88% identity, 89% coverage) similar to pOZ176, respectively.

In addition to this system, pTTS12 contains another putative partitioning system encoded on RPPX_26215, RPPX_26845 and RPPX_26840 loci. RPPX_26215 putatively encodes for TrfA protein, which is essential for initiating DNA replication at the origin of repilcation. The protein encoded at RPPX_26840 locus appears to belong to the ParB/RepB/Spo0J (KorB) family partition protein. This family of partition protein is known as a regulator of replication and maintenance of the IncP plasmids by inhibiting *trfA* expression (Kornacki et al., 1987; Rosche et al., 2000). Typical to low copy plasmid, this putative ParA-ParB protein pair may act as an active partitioning system to ensure that a copy of the plasmid segregates to each daughter cell during cell division by targeting a nearby centromere-like element (Rosche et al., 2000).

### Distinctive conjugation machinery of pTTS12

**FIGURE 2:**
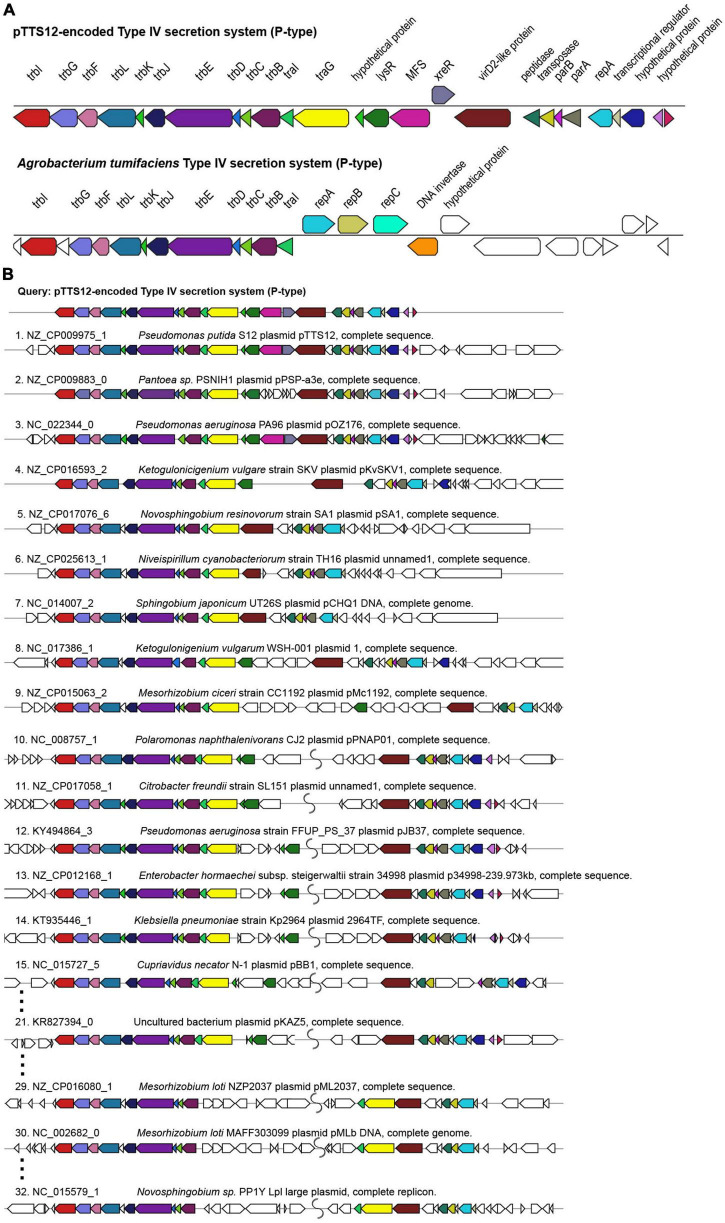
Structure and synteny of pTTS12 conjugation system. **(A)** The arrangement of the T4SS gene cluster found in pTTS12 and the prototype *trb* operon from *Agrobacterium tumefaciens* (pTiC58). The colors represent different genes in the cluster and same colors are assigned for the homologous genes. The gene names are indicated above the respective clusters. pTTS12/T4SS and the *trb* operon of pTiC58 share synteny for 11 genes (*trbI* to *traI*), while other parts are clearly different. **(B)** Synteny plot of the T4SS gene cluster of pTTS12 for plasmid conjugation, replication and partitioning compared with other plasmids. This visualization was generated using multigeneblast software (Medema et al., 2013). The numbers refer to the order of decreasing synteny. For the sake of clarity, several plots were removed from this figure, indicated by the dots. The colors represent different genes in the cluster corresponding to color-coding in panel **(A)**. Putative coupling-protein (T4CP) *traG* is indicated in yellow and the putative relaxase *virD2*-like protein is indicated in brown.

Megaplasmid pTTS12 contains a P-type T4SS (Type IV secretion system) conjugation system (RPPX_28670-RPPX_28725), sharing synteny with the prototype *trb* operon from *A. tumefaciens* pTiC58 (Figure 2A). For an operational T4SS conjugation machinery, a T4SS gene cluster, type IV coupling protein (T4CP) and a relaxase protein are required (Christie et al., 2014). An additional *traG* (RPPX_28650), which may serve as a T4CP, is located upstream of the T4SS cluster. Further upstream of the T4SS and T4CP, a putative virD2-like gene (RPPX_28750) and an operon consisting of *parB*, *parA* and *repA* (RPPX_28765-28775) are encoded on pTTS12. The putative virD2-like gene (RPPX_28750) or the ATP-dependent exonuclease RecD (RPPX_28655) may play a role as a relaxase, which is important for the transferability of pTTS12.

T4SS, *traG*, and upstream genes involved in replication and partitioning shared synteny with the T4SS clusters on other plasmids such as *Pantoea sp.* PSNIH1 (pPSP-a3e), *Pseudomonas aeruginosa* PA96 (pOZ1760), and *Ketogulonicigenium vulgare* SKV (pKvSKV1) (Figure 2B). Typically, *traG* (T4CP) is coupled to the same operon of T4SS while the region encoding replication and partitioning may be separated (Figure 2B), in some cases relatively far. It is interesting to note that in two *Mesorhizobium loti* strains and *Novosphingobium sp.* PP1Y, the T4SS operon and *traG-traI* are completely separated (Figure 2B). Instead, VirD2-like protein (relaxase), *traG* (T4CP) and *traI* formed a single operon.

Most of the Pseudomonadaceae-family plasmids do not contain this *trb* operon (Figure 1) except for pTTS12, pOZ176 from *P. aeruginosa* PA96, pJB37 from *P. aeruginosa* FFUP_PS_37 and an unnamed plasmid from *P. aeruginosa* PA83. Therefore, this conjugative operon may not be common in IncP-2 plasmid family. However, this *trb* operon is abundant among Enterobacteriaceae-family plasmids (Figure 1).

### The unique arrangement of Tn*3* transposable elements is important for the dissemination of styrene degradation pathway

pTTS12 shares 21% of sequence similarity with the pSTY plasmid of *P. taiwanensis* VLB120. The major and only gene clusters shared between pTTS12 and pSTY are involved in styrene degradation, phenylpropionic acid degradation, and solvent efflux pump (SrpABC). The genes shared between pTTS12 and pSTY are absent in all other *Pseudomonas* plasmids, except for the solvent efflux pump gene cluster which is similar with TtgGHI efflux pump from the pGRT-1 plasmid of *P. putida* DOT-T1E. The regions encoding solvent efflux pump and phenylpropionic acid degradation are clustered together and have identical synteny in pTTS12 and pSTY (Supplementary Figure 2). However, pTTS12 and pSTY do not share any mobile genetic elements surrounding the solvent efflux pump and phenylpropionic acid degradation gene clusters that might indicate a shared mechanism of acquisition or transfer.

**FIGURE 3:**
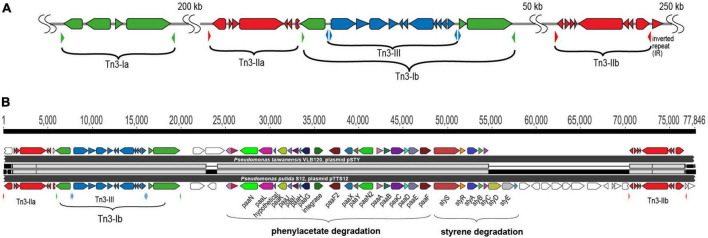
Tn*3* transposon family elements responsible for horizontal gene transfer of styrene and phenylacetate degradation pathway. **(A)** Genetic organization of the three different Tn*3*-family transposable elements in pTTS12. Colors represent the three different Tn*3* transposon families; Tn*3-I* with highest identity to Tn*4656* (green), Tn*3-IIa* and *b* with highest identity to Tn*5053* (red) and Tn*3-III* with highest identity to Tn*5563* (blue). The inverted repeats (IRs) flanking each element are marked by small, lowered triangles in the same colors, accordingly. **(B)** Alignment of the Tn*3*-mediated horizontal gene transfer of the styrene and phenylacetate degradation cluster in pTTS12 (bottom) and pSTY from *Pseudomonas taiwanensis* VLB120 (top). Colors represent the different genes constituting styrene-phenylacetate degradation cluster and the three Tn*3* transposable elements, corresponding to the color-coding in panel A. Gene names are indicated.

The unique arrangement of the three different Tn*3* elements in pTTS12 is present on pSTY of *P. taiwanesis* VLB120, including an additional copy of Tn*5563*. In between these two Tn*5563*-elements, both megaplasmids contain the complete styrene degradation pathway, enabling both strains to grow on styrene as sole carbon source (Köhler et al., 2013; Kuepper et al., 2015). Detailed comparison of the regions between the two Tn*5563* elements from pTTS12 and pSTY reveals a high similarity between Tn*5563-I* and Tn*5563-II* (Figure 3B). This sequence is around 77 kbps and 60 kbps for pTTS12 and pSTY respectively, including the IRs bracketing both sequences.

pTTS12 as well as many other highly similar plasmids contain a multitude of mobile genetic elements, such as ISS12 and Tn*3*-family transposases (Hall et al., 2015). pTTS12 harbors five copies of Tn*3*-family transposable elements; two copies of Tn*3-I* with highest identity to Tn*4656*, two copies of Tn*3-II* that are highly similar to Tn*5053* and a single copy of Tn*3-III* with highest similarity to Tn*5563* (Figure 3A). Tn*3-I* is an identical transposase (RPPX_RS27515, *tnpR* and RPPX_RS27515, *tnpA*) to Tn*4656* encoded on pWW53 plasmid from *P. putida* MT53. Moreover, the 39-bps inverted repeat (IR) sequences found on both ends of these two elements are highly identical with only a single mismatch difference. In addition to *tnpA* and *tnpR*, this Tn*3*-element contains a putative methyl-accepting chemotaxis protein (*mcpT-2*) and an insertion sequence IS256. Tn*3-II* of pTTS12 is identical (99.8% similarity) to Tn*5053* of *Xanthomonas* sp. W17 encoding a mercury resistance gene cluster *merR* and *merTPAD* (Kholodii et al., 1993). This Tn*3-II* has 25 bp inverted repeats, bracketing the ends and 5 bp directed repeats (DR). Tn*3-III* is identical to Tn*5563*, that is found in several other plasmids, e.g., pAMBL of *P. aeruginosa*, pSTY of *P. taiwanensis* VLB-120 and pRA2 of *P. alcaligenes*. The Tn*3-III* has identical 38 bps IRs bracketing the element, also identical to IRs found for this element in other plasmids. This element contains several genes such as *merR*, *merTP*, *pilT* and a gene with a PIN nuclease domain.

The Tn*4656* and Tn*5563* are both duplicated and rearranged on the megaplasmid together with Tn*5053*. This rearrangement resulted in a highly characteristic sequence, which partially resulted from insertion of Tn*5053* transposition in the second copy of Tn*4656* (Tn*4656-II*), between the *mcpT*-2 and *tnpR* loci (Figure 3). The IRs as well as DRs of Tn*5053* indicate the insertion site of this element. The IRs of Tn*4656-II* are well preserved bracketing both elements. Other parts of this sequence consist of the second copy of Tn*5563* (Tn*5563-II*), which is truncated at the right end by insertion of Tn*4656-II* in merT and thus truncated the 5’ end of *merT* sequence, can be identified. Due to this truncation, the IRs of *Tn4656-II* cannot be found on the right side of the element anymore except for the right IR of initial Tn*4656*, which is located 58 kbps upstream of this element.

### Conjugative megaplasmid pTTS12 is highly stable in *Pseudomonas putida* KT2440

To characterize the conjugative transferability of pTTS12, we performed biparental mating between *P. putida* S12.1 and *P. putida* KT-BG35. *P. putida* KT-BG35 is a strain derived from *P. putida* KT2440, does not harbor a megaplasmid and carries a gentamicin resistant marker and green fluorescence protein (GFP) at its *attn7* site (Zobel et al., 2015). The transfer was performed using biparental mating between *P. putida* S12.1 containing pTTS12 with kanamycin resistant marker and *P. putida* KT-BG35. Transconjugant colonies resistant to both kanamycin and gentamicin occurred at the frequency of 4.20 (±0.51) × 10^–7^. After appropriate selection on agar plates, the identity of transconjugant colonies was confirmed by observing GFP expression of the colonies derived from *P. putida* KT-BG35. Additionally, transfer of entire pTTS12 was confirmed using PCR. Amplification of several regions of pTTS12 using primer pairs 53,496_Fw-56,596_Rv, 200,497_Fw-203,602_Rv, and 286,448_Fw-289,462_Rv resulted in an expected band of 3,100, 511, and 3,015 bp in transconjugant colonies, respectively (Supplementary Figure 5). Fifteen randomly selected colonies chosen for colony PCR showed correct bands. This confirmed the transfer of entire pTTS12 into *P. putida* KT-BG35 and the resulting strains will further be referred to as *P. putida* KTpS12. Several attempts to transfer pTTS12 from *P. putida* S12.1 into *E. coli* strains represented by *E. coli* MV1190 and *E. coli* XL1-Blue by biparental mating did not result in any successful transconjugant colonies.

**FIGURE 4:**
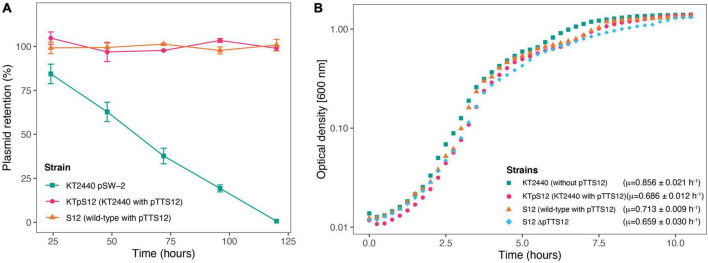
Megaplasmid pTTS12 is highly stable in *P. putida* strains and reduces maximal growth rate in *P. putida* KT2440. **(A)** Plasmid retention of pTTS12 in *P. putida* KT2440 and *P. putida* S12 on liquid LB without selection pressure for approximately 50 generations. Colors and shapes indicate the different strains with green squares indicating *P. putida* KT2440 pSW-2, magenta circles indicating *P. putida* KTpS12, and orange triangles indicating *P. putida* S12 (containing pTTS12). Plasmid pSW-2 in *P. putida* KT2440 was used as a control for loss of unstable plasmid. pTTS12 is stably maintained in both *P. putida* S12 and *P. putida* KTpS12. **(B)** Growth curve of *P. putida* KT2440, *P. putida* KTpTTS12 *P. putida* S12, and *P. putida* S12 ΔpTTS12. Growth was followed on liquid LB at 30°C, 200 rpm shaking. Growth curves represent data obtained from three biological replicates for each strain, starting from OD_600*nm*_ = 0.05. Green squares indicate *P. putida* KT2440 pSW-2, magenta circles indicate *P. putida* KTpS12, orange triangles indicate *P. putida* S12 (containing pTTS12), and blue diamond indicate *P. putida* S12 ΔpTTS12. Maximum growth rates calculated for each strais are indicated within the figure.

The stability of pTTS12 in *P. putida* KTpS12 and *P. putida* S12 was examined for 5 passages in the absence of antibiotic selective pressure (approximately 10 generations/passage step). No plasmid loss was observed from either *P. putida* KTpS12 or *P. putida* S12 growing without selective pressure while the negative control pSW-2 showed a steady plasmid loss (Figure 4A). Serendipitous deletion of pTTS12 was not found in *P. putida* S12 nor *P. putida* KTpS12. Hence, pTTS12 is a stable megaplasmid in both *P. putida* S12 and *P. putida* KT2440.

The occurrence of large plasmids in bacteria may cause metabolic burden (Wein et al., 2020), which is reflected in reduced bacterial growth rate (μ). *P. putida* KTpS12 exhibited a lower maximum growth rate compared to *P. putida* KT2440, 0.686 ± 0.012 and 0.856 ± 0.021 h^–1^ respectively (Figure 4B). However, *P. putida* S12 and *P. putida* S12 ΔpTTS12 did not show a significant maximum growth rate difference (0.713 ± 0.009 and 0.659 ± 0.030 h^–1^ respectively). The length of lag-phase and biomass yield at stationary phase remained unaffected with pTTS12 present in both *P. putida* S12 and KTpS12. Apparently, the presence of pTTS12 imposes significant metabolic burden in *P. putida* KT2440 (Student’s *t*-test, *p*-value 0.0002553), but not in S12.

### Phenotypic features of pTTS12 and attained solvent tolerance in *Pseudomonas putida* KTpS12

**FIGURE 5:**
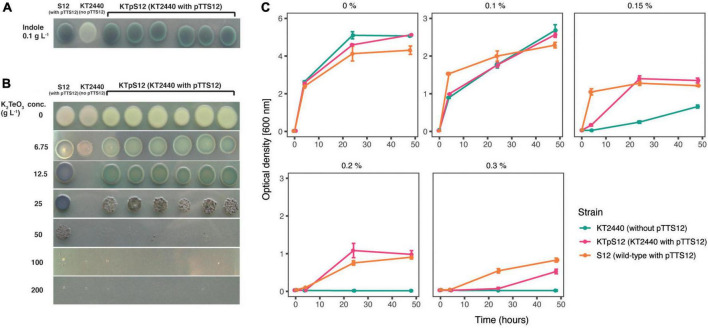
Transfer of pTTS12 phenotypic characteristics. **(A)** Production of indigo from indole indicating activity of styrene monooxygenase (StyA) and styrene oxide isomerase (StyB). Minimal medium (M9) was supplemented with indole which, in the presence of StyAB enzymes encoded on pTTS12, is converted into indigo. Positive control *P. putida* S12 showed indigo coloration, whereas negative control *P. putida* KT2440 remained white. *P. putida* KTpS12 showed indigo coloration indicating activity of pTTS12-encoded *styAB*. **(B)** Growth of *P. putida* strains in the presence of potassium tellurite (K_2_TeO_3_). Minimum inhibitory concentration (MIC) of positive control *P. putida* S12 was 200 g L^– 1^ whereas the MIC of the wild-type *P. putida* KT2440 was 12.5 g L^– 1^. The MIC of *P. putida* KTpS12 was 100 g L^– 1^ indicating the presence and activity of the *ter* operon on pTTS12. **(C)** Growth of *P. putida* strains on increasing concentrations of toluene. Optical density (OD_600*nm*_) of the cultures was measured at 4, 24, and 48-h time points with starting point of OD_600*nm*_ = 0.1. The y-axis range is different between the first panel (0–6) and the other panels (0–3). Green dots indicate *P. putida* KT2440, magenta dots indicate *P. putida* KTpS12, and orange dots indicate *P. putida* S12 (containing pTTS12). *P. putida* KTpS12 showed an increase in solvent tolerance indicating the presence and acivity of the *srp* operon from pTTS12.

*P. putida* S12 is intrinsically tolerant to an assortment of environmental xenobiotics. The majority of these characteristic traits are encoded on pTTS12; the styrene degradation operon *styABCDE*, the tellurite resistance operon *terZABCDE* and a solvent efflux pump *srpABC*. To explore functionality of pTTS12 following conjugative transfer, these characteristic traits were investigated in *P. putida* KTpS12 (Figure 5). Activity of the styrene degradation operon in *P. putida* KTpS12 was tested by growing on minimal media with styrene as carbon source and inspecting transformation of indole into indigo. Similarly as observed in *P. putida* S12, *P. putida* KTpS12 was able to transform indole into indigo, whereas wild-type KT2440 was not, indicating activity of styrene monooxygenase (*styA*) and styrene oxide isomerase (*styB*) in these strains (Figure 5A). In addition, *P. putida* KTpS12 was able to grow on minimal media supplemented with styrene as carbon source or minimal media agar plates incubated in styrene atmosphere (data not shown).

Potassium tellurite (K_2_TeO_3_) exhibits antimicrobial activity in bacteria. Tellurite resistance in bacteria is achieved through reduction of tellurite (TeO_3_^2–^) into a less toxic metallic tellurium (Te^0^), causing the formation of black colonies in the presence of tellurite (Taylor, 1999). The resistance genes on pTTS12 are encoded as an operon *terZABCDE* (RPPX_26360-26385). To investigate whether this gene cluster is able to increase resistance toward tellurite in *P. putida* KT2440, the minimum inhibitory concentration (MIC) was determined for the three different stains (Figure 5B). The MIC of potassium tellurite for *P. putida* KT2440 was 16-fold less compared to *P. putida* S12, 12.5 and 200 g L^–1^ respectively. *P. putida* KTpS12 showed an 8-fold increase in tellurite resistance (MIC 100 g L^–1^) compared to its parental strain *P. putida* KT2440.

The solvent efflux pump *srpABC* encoded on the megaplasmid pTTS12, enables survival and growth of *P. putida* S12 in non-utilized organic solvents (Kieboom and de Bont, 2001). In order to investigate the effect of induced tolerance toward organic solvents due to the introduction of pTTS12 in *P. putida* KTpS12, a growth assay was performed in the presence of different toluene concentrations (Figure 5C). *P. putida* KT2440 was able to grow in LB liquid media supplemented with toluene up to a maximum of 0.15% v/v concentration, although demonstrating a significant growth reduction compared to *P. putida* S12. With the introduction of pTTS12, *P. putida* KTpS12 tolerance toward toluene increased to 0.30% v/v. Similar concentration was obtained for *P. putida* S12, while *P. putida* KTpS12 exhibited slightly slower growth in the presence of toluene. These observations indicated that the introduction of megaplasmid pTTS12 provided a full set of characteristic features to *P. putida* KT2440.

## Discussion

### Megaplasmid pTTS12 defines an environment-adapted member of IncP-2 family plasmids, carrying distinct accessory gene clusters

Megaplasmid pTTS12 of *P. putida* S12 is closely related to other plasmids in proteobacteria, especially within the *Pseudomonas* genus. In this study we demonstrated high similarity of the pTTS12 ‘backbone’ with pOZ176 from *P. aeruginosa* PA96 (Xiong et al., 2013) and several other IncP-2 plasmids, such as pJB37 of *P. aeruginosa* FFUP_PS_37 (Botelho et al., 2017). pOZ176 has been categorized within incompatibility group IncP-2 based on its replication, partitioning, and transfer machinery along with conferring tellurite resistance as a key feature of IncP-2 plasmid (Xiong et al., 2006, 2013). Indeed, pTTS12 encodes typical characteristics of IncP-2 plasmids, such as heavy metal resistance (tellurite, mercury, and chromate) and plasmid maintenance via the *parA*/*parB*/*repA* system (Jacoby et al., 1983; Shintani et al., 2015). Despite high similarity between pTTS12 and pOZ176, pTTS12 lacks the Tn*6016 bla*_*IMP–*9_-carrying class 1 integron cassette which is an important trait of pOZ176 conferring resistance to aminoglycosides and carbapenems (Xiong et al., 2006, 2013). On the other hand, pOZ176 lacks the solvent efflux pump, styrene degradation pathway, and phenylpropionic acid degradation pathway which are main characteristics of pTTS12. This demonstrates that very similar megaplasmid backbones may carry resistance to a diversity of xenobiotics, metabolic functions, or virulence gene clusters (Harrison and Brockhurst, 2012). Whereas the traits disseminated by pTTS12 and pOZ176 are highly divergent and distinctive, a similar observation of environment-adaptive traits conferred by flexible and diverse accessory gene clusters contained on IncP-2 plasmids was recently reported for *P. aeruginosa* plasmids pBT2436 and pBT2101, carrying multiple MDR cassettes (Cazares et al., 2020).

### Convergent distribution of plasmid-encoded solvent tolerance gene clusters

pTTS12 shares its unique features of styrene and phenyl-propanoate degradation pathway with pSTY from *P. taiwanensis* VLB120 and the solvent extrusion pump SrpABC with TtgGHI from pSTY and pGRT-1 *P. putida* DOT-T1E. Hence, these plasmids isolated from different environmental sources show convergent organization of highly similar gene clusters with similar features related to environmental stresses (e.g., organic solvents) on different plasmid backbones. In pSTY, the arrangement of gene clusters encoding the styrene degradation pathway and solvent efflux pump - phenylpropionic acid degradation pathway is highly similar to pTTS12, with 99 and 80% similarity, respectively. This suggests that exchange of the styrene degradation pathway occurred more recently than exchange of the efflux pump/phenylpropionic acid degradation cluster.

The styrene degradation gene cluster is encoded within a unique arrangement of Tn*3* family transposases shared by pTTS12 and pSTY (Figure 3). Due to the transposition of Tn*3-Ib*, Tn*3-IIa* lost its right flank inverted repeat (IR). Because of this arrangement, Tn*3-IIa* may only “jump” while carrying the entire Tn*3* and styrene-phenylacetate degradation clusters using the right flank of Tn*3-IIb* IR. This would explain the occurrence of the styrene-phenylacetate degradation cluster distinctive arrangement shared between pTTS12 and pSTY. Although we could not find evidence of mobile genetic elements carrying the efflux pump and the phenylpropionic acid degradation gene clusters on pSTY and pTTS12, it is well possible that exchange originally occurred via such route.

### Transferability of pTTS12

pTTS12 contains a P-Type type IV secretion system (T4SS) with synteny similar to the prototype *trb* operon of the *A. tumefaciens* pTiC58 system (Li et al., 1999; Christie et al., 2014) and it is self-transferable toward other *P. putida* strains. Interestingly, this locus contains a putative relaxase encoded by virD2 (RPPX_28750, Figure 2). This relaxase is predicted to be responsible for creating a nick prior to plasmid transfer via the conjugative bridge. However, complete deletion of *virD2* did not result in a significant reduction of transfer frequency (4.32 (± 1.53) × 10***^–^***^7^; Student’s t-test performed, *p*-value 0.9078), compared to the wild-type pTTS12. This may indicate the presence of other relaxase(s) which act in-trans with this secretion system. *P. putida* S12 contains other T4SS within its genome which shares homology with the I-type T4SS represented by Dot/Icm from *L. pneumophila* (Supplementary Figure 3), although it is unclear whether Dot/Icm can support the transfer of pTTS12 (Nagai and Kubori, 2011). Further research is required to establish putative crosstalk between these secretion systems.

### Stable and efficient accommodation of mega plasmid in *Pseudomonas putida* S12

We observed that in its original host *P. putida* S12 on rich media, pTTS12 did not impose an apparent metabolic burden whereas in another strain as *P. putida* KT2440, pTTS12 caused a 20% reduction of maximum growth rate. We previously reported on the occurrence of metabolic burden caused by pTTS12 in *P. putida* S12 in the presence of toluene (Kusumawardhani et al., 2020). In addition, pTTS12 was stably maintained in both *P. putida* S12 and *P. putida* KT2440 (Figure 4A). Apparently, *P. putida* S12 has found ways to accommodate reduced fitness cost, metabolic burden, and stability of this mega plasmid.

Conjugative transfer may impose substantial cost and burden due to the energy investment on pili formation during conjugation (Turner et al., 1998; Harrison and Brockhurst, 2012). Indeed, pTTS12 showed a substantially lower conjugation rate (4.20 × 10***^–^***^7^ transfer frequency after 24 h) in comparison to other IncP-2 plasmids (10***^–^***^1^ to 10***^–^***^4^ transfer frequency after 2 h) (Jacoby et al., 1983). Moreover, the expression of plasmid-encoded resistance genes, like the solvent extrusion pump, can typically be a source of metabolic burden imposed by plasmids (MacLean and San Millan, 2015). pTTS12 contains multiple types of mobile genetic elements, with ISS12 being the most abundant (Wery et al., 2001; Hosseini et al., 2017). Substantial duplication of the ISS12 mobile element has previously been reported to interrupt *srpA*, encoding the periplasmic subunit of the solvent efflux pump (Hosseini et al., 2017). In the prolonged absence of organic solvent, expression and maintenance of the solvent efflux pump may be costly for the bacterial cell, hence, interruption of the *srp* efflux pump gene cluster may further reduce the plasmid burden of pTTS12. In addition to these mechanisms, we recently described the contribution of a toxin-antitoxin module SlvT-SlvA to the stability of pTTS12 (Kusumawardhani et al., 2020).

### Future outlook

IncP-2 family plasmids are widely distributed among environmental and clinical *Pseudomonas* isolates. These plasmids contain variable regions encoding MDR, xenobiotics extrusion pumps, degradation pathways, and heavy metal resistance cassettes. In contrast to the dynamic variable regions, the core backbone of these plasmids shows a general conservation. It is interesting to discover the minimal backbone of IncP-2, which enables Pseudomonads to scavenge gene clusters important for its survival both in environmental and clinical set-up, as a model of horizontal gene transfer. Ultimately, the IncP-2 plasmid family appears to be promising for biotechnological and bioremediation applications due to its stability and relatively low metabolic burden. On the other hand, these plasmids may exchange their traits, thus creating hybrid plasmids when they occur within the same host (Jacoby et al., 1983). The mechanisms for such exchange are still poorly understood and may well involve transposition and conjugation as suggested by the shared and type-specific characteristics of pTTS12 and pOZ176 as described in this study. Further clarification of these mechanisms is important to shed light on the rapid dissemination of bacterial tolerance and resistance to antibiotics, and chemical stresses in the environment.

Successful attempts have been made in exploiting traits from environmental plasmids for standardized components in synthetic biology (Martínez-García et al., 2015). Environmental plasmids are exchangeable between different hosts and may express their genetic features in various genomic and metabolic backgrounds. Moreover, they are an excellent source of novel biological parts such as origin of replication, metabolic pathway, resistance marker, and regulated promoters. New sequencing technologies and comparative genomics analyses support the identification of the genes enabling these features. Here, we demonstrated that pTTS12 contains promising exchangeable gene clusters and building blocks to construct robust microbial hosts for high-value biotechnology applications.

## Data availability statement

The original contributions presented in this study are included in the article/Supplementary material, further inquiries can be directed to the corresponding author.

## Author contributions

HK, RH, and J-AV performed the data acquisition. HK and RH contributed to the data analysis and wrote the main text of the manuscript. HK, RH, and JHdW did the conceptualization, reviewing, and editing of the manuscript. All authors contributed to the article and approved the submitted version.

## Footnotes

1
https://www.ncbi.nlm.nih.gov/refseq/

2
https://www.ncbi.nlm.nih.gov/nuccore/
