# Erratum: Comparative analysis reveals the modular functional structure of conjugative megaplasmid pTTS12 of *Pseudomonas putida* S12: A paradigm for transferable traits, plasmid stability, and inheritance?

**DOI:** 10.3389/fmicb.2023.1176489

**Published:** 2023-03-13

**Authors:** Frontiers Production Office

**Affiliations:** Frontiers Media SA, Lausanne, Switzerland

**Keywords:** *Pseudomonas putida*, genome sequence, solvent tolerance, megaplasmid, mobile genetic elements, comparative analysis

An omission to the funding section of the original article was made in error. The following sentence has been added: “Open access funding was provided by the University of Lausanne.”

The publisher apologizes for this mistake. The original article has been updated.

