# Supplementary material for: Comparative analysis reveals the modular functional structure of conjugative megaplasmid pTTS12 of Pseudomonas putida S12: A paradigm for transferable traits, plasmid stability, and inheritance?
Source: Front Microbiol. 2022 Sep 23;13:1001472. doi: 10.3389/fmicb.2022.1001472 (PMC9537497; doi:10.3389/fmicb.2022.1001472)
Supplement: Supplementary file 1 [file Data_Sheet_1.PDF]

Table S1. Extended annotations of pTTS12

locus_tag	lo.min	lo.max	Direction	X	df_s12.mp.Operor	n	f.cluster	clus.x		f.cluster_function	f.cluster_fmain	Gene_function	gene_name	Comments	cds
**RPPX_RS25895**	294	752	forward	1	operon_0001	2	fcluster_0013	1		hypothetical	Unknown				hypothetical protein CDS
**RPPX_RS25900**	763	1134	forward	2	operon_0001	5	fcluster_0013	1		hypothetical	Unknown				hypothetical protein CDS
**RPPX_RS25905**	1253	1765	reverse	3	operon_0002	6	fcluster_0013	1		hypothetical	Unknown				hypothetical protein CDS
**RPPX_RS25910**	1881	2744	reverse	4	operon_0002	182	fcluster_0013	1		hypothetical	Unknown				DNA polymerase III subunit epsilon CDS
**RPPX_RS25915**	2768	3256	reverse	5	operon_0002	3	fcluster_0013	1		hypothetical	Unknown				hypothetical protein CDS
**RPPX_RS25920**	3578	3811	reverse	6	operon_0003	1	fcluster_0013	1		hypothetical	Unknown				hypothetical protein CDS
**RPPX_RS25925**	4091	4450	reverse	7	operon_0004	6	fcluster_0013	1		hypothetical	Unknown				hypothetical protein CDS
**RPPX_RS25930**	4742	6124	reverse	8	operon_0005	5	fcluster_0013	1		hypothetical	Unknown				hypothetical protein CDS
**RPPX_RS25935**	6284	6760	forward	9	operon_0006	6	fcluster_0013	1		hypothetical	Unknown				hypothetical protein CDS
**RPPX_RS25940**	6894	7511	reverse	10	operon_0007	6	fcluster_0013	1		hypothetical	Unknown				hypothetical protein CDS
**RPPX_RS25945**	7649	7873	reverse	11	operon_0007	6	fcluster_0013	1		hypothetical	Unknown				hypothetical protein CDS
**RPPX_RS25950**	8058	9035	forward	12	operon_0008	5	fcluster_0013	1		hypothetical	Unknown				hypothetical protein CDS
**RPPX_RS25955**	9063	9770	reverse	13	operon_0009	6	fcluster_0013	1		hypothetical	Unknown				hypothetical protein CDS
**RPPX_RS25960**	9942	10802	forward	14	operon_0010	7	fcluster_0013	1		hypothetical	Unknown				prepilin-type N-terminal cleavage/methylation domain-containing protein CDS
**RPPX_RS25965**	10948	11772	forward	15	operon_0010	5	fcluster_0013	1		hypothetical	Unknown				hypothetical protein CDS
**RPPX_RS25970**	11801	12364	reverse	16	operon_0011	6	fcluster_0013	1		hypothetical	Unknown				hypothetical protein CDS
**RPPX_RS25975**	12607	13035	forward	17	operon_0012	4	fcluster_0013	1		hypothetical	Unknown				hypothetical protein CDS
**RPPX_RS25980**	13259	14416	forward	18	operon_0013	20	fcluster_0013	1		hypothetical	Unknown				hypothetical protein CDS
**RPPX_RS25985**	14427	16034	forward	19	operon_0013	28	fcluster_0013	1		hypothetical	Unknown				hypothetical protein CDS
**RPPX_RS25990**	16790	17872	forward	20	operon_0014	8	fcluster_0015	2		Stress response	General stress response				site-specific recombinase CDS
**RPPX_RS25995**	17898	18281	forward	21	operon_0014	36	fcluster_0015	2		Stress response	General stress response				TetR family transcriptional regulator CDS
**RPPX_RS26000**	18372	19334	reverse	22	operon_0015	52	fcluster_0015	2		Stress response	General stress response				DNA-binding protein CDS
**RPPX_RS26005**	19381	19734	reverse	23	operon_0015	48	fcluster_0015	2		Stress response	General stress response				hypothetical protein CDS
**RPPX_RS26010**	19740	20591	reverse	24	operon_0015	128	fcluster_0015	2		Stress response	General stress response				universal stress protein A CDS
**RPPX_RS26015**	20606	22093	reverse	25	operon_0015	235	fcluster_0015	2		Stress response	General stress response				sulfate transporter CDS
**RPPX_RS26020**	22582	23700	forward	26	operon_0016	11	fcluster_0020	3		DNA repair	Replication/partitioning	DNA polymerase V subunit	umuC		hypothetical protein CDS
**RPPX_RS26025**	24029	24406	forward	27	operon_0017	4	fcluster_0020	3		DNA repair	Replication/partitioning				hypothetical protein CDS
**RPPX_RS26030**	24431	27367	reverse	28	operon_0018	6	fcluster_0020	3		DNA repair	Replication/partitioning				hypothetical protein CDS
**RPPX_RS26035**	27908	30496	forward	29	operon_0019	10	fcluster_0020	3		DNA repair	Replication/partitioning				outer membrane autotransporter barrel domain-containing protein CDS
**RPPX_RS26040**	30575	31420	forward	30	operon_0019	17	fcluster_0020	3		DNA repair	Replication/partitioning				hypothetical protein CDS
**RPPX_RS26045**	31447	31743	forward	31	operon_0019	5	fcluster_0020	3		DNA repair	Replication/partitioning				hypothetical protein CDS
**RPPX_RS26050**	31775	32302	forward	32	operon_0019	4	fcluster_0020	3		DNA repair	Replication/partitioning				hypothetical protein CDS
**RPPX_RS26055**	32546	33601	reverse	33	operon_0020	NA	fcluster_0020	3		DNA repair	Replication/partitioning	Site-specific tyrosine recombinase	xerD	http://www.uniprot.org/uniprot/P0A8P8	recombinase CDS
**RPPX_RS26060**	33700	34083	reverse	34	operon_0020	6	fcluster_0020	3		DNA repair	Replication/partitioning				hypothetical protein CDS
**RPPX_RS26065**	34281	34706	forward	35	operon_0021	5	fcluster_0024	4		hypothetical	Unknown				hypothetical protein CDS
**RPPX_RS26070**	34708	35004	forward	36	operon_0021	5	fcluster_0024	4		hypothetical	Unknown				hypothetical protein CDS
**RPPX_RS26075**	35011	36591	reverse	37	operon_0022	15	fcluster_0024	4		hypothetical	Unknown				hypothetical protein CDS
**RPPX_RS26080**	36732	38000	reverse	38	operon_0022	9	fcluster_0024	4		hypothetical	Unknown				hypothetical protein CDS
**RPPX_RS26085**	38056	38529	reverse	39	operon_0022	21	fcluster_0024	4		hypothetical	Unknown				hypothetical protein CDS
**RPPX_RS26090**	38849	39556	reverse	40	operon_0023	5	fcluster_0024	4		hypothetical	Unknown	secretion	tolA	http://pfam.xfam.org/family/PF06519	hypothetical protein CDS
**RPPX_RS26095**	39699	40481	reverse	41	operon_0023	NA	fcluster_0024	4		hypothetical	Unknown				hypothetical protein CDS
**RPPX_RS26100**	40564	41166	reverse	42	operon_0023	4	fcluster_0024	4		hypothetical	Unknown				hypothetical protein CDS
**RPPX_RS26105**	41163	41402	reverse	43	operon_0023	3	fcluster_0024	4		hypothetical	Unknown				hypothetical protein CDS
**RPPX_RS29120**	41466	42413	reverse	44	operon_0023	NA	fcluster_0024	4		hypothetical	Unknown				hypothetical protein CDS
**RPPX_RS26115**	42569	43849	reverse	45	operon_0024	15	fcluster_0024	4		hypothetical	Unknown	dna metylation	pvuIIM		hypothetical protein CDS
**RPPX_RS29125**	43975	44331	reverse	46	operon_0024	NA	fcluster_0024	4		hypothetical	Unknown				hypothetical protein CDS
**RPPX_RS26120**	44803	45927	forward	47	operon_0025	165	fcluster_0025	5		MGE	MGE	Shufflon-specific DNA recombinase			integrase CDS
**RPPX_RS26125**	47920	48681	reverse	48	operon_0026	NA	fcluster_0031	6		Pilus assembly	Conjugation				pili assembly chaperone CDS
**RPPX_RS26130**	48839	49273	reverse	49	operon_0027	16	fcluster_0031	6		Pilus assembly	Conjugation		relA	http://www.uniprot.org/uniprot/P0AG20	GTP diphosphokinase CDS
**RPPX_RS26135**	49348	49692	reverse	50	operon_0027	4	fcluster_0031	6		Pilus assembly	Conjugation				hypothetical protein CDS
**RPPX_RS26140**	49863	50285	forward	51	operon_0028	2	fcluster_0031	6		Pilus assembly	Conjugation				hypothetical protein CDS
**RPPX_RS26145**	50340	50975	reverse	52	operon_0029	153	fcluster_0031	6		Pilus assembly	Conjugation				hypothetical protein CDS
**RPPX_RS26150**	51302	52615	reverse	53	operon_0030	4	fcluster_0031	6		Pilus assembly	Conjugation				hypothetical protein CDS
**RPPX_RS26155**	52775	53656	reverse	54	operon_0031	79	fcluster_0031	6		Pilus assembly	Conjugation				hypothetical protein CDS
**RPPX_RS26160**	53900	54655	reverse	55	operon_0032	373	fcluster_0032	7		ISS12_C_ATCC	MGE			ISS12_C_ATCC	ATPase AAA CDS
**RPPX_RS26165**	54676	56190	reverse	56	operon_0032	172	fcluster_0032	7		ISS12_C_ATCC	MGE			ISS12_C_ATCC	integrase CDS
**RPPX_RS26170**	56623	57873	reverse	57	operon_0033	53	fcluster_0035	8		Tn3	MGE				transposase CDS
**RPPX_RS26175**	58041	59699	reverse	58	operon_0034	16	fcluster_0035	8		two-component system	Signal transduction				chemotaxis protein CDS
**RPPX_RS26180**	60041	60601	forward	59	operon_0035	627	fcluster_0035	8		Tn3	MGE		tnpR		Tn4656/Tn4658 resolvase CDS
**RPPX_RS26185**	60605	63571	forward	60	operon_0035	225	fcluster_0035	8		Tn3	MGE		tnpA		transposase CDS
**RPPX_RS26190**	63674	64036	forward	61	operon_0035	5	fcluster_0038	9		hypothetical	Unknown				hypothetical protein CDS
**RPPX_RS26195**	64033	64953	reverse	62	operon_0036	4	fcluster_0038	9		hypothetical	Unknown				hypothetical protein CDS
**RPPX_RS26200**	65010	65192	reverse	63	operon_0036	1	fcluster_0038	9		hypothetical	Unknown				hypothetical protein CDS
**RPPX_RS26205**	65374	66408	forward	64	operon_0037	5	fcluster_0038	9		hypothetical	Unknown				hypothetical protein CDS
**RPPX_RS26210**	66542	67747	forward	65	operon_0037	12	fcluster_0038	9		hypothetical	Unknown				hypothetical protein CDS
**RPPX_RS26215**	67779	68870	reverse	66	operon_0038	13	fcluster_0038	9		hypothetical	Unknown				hypothetical protein CDS
**RPPX_RS26220**	70990	72285	reverse	67	operon_0039	14	fcluster_0039	10		two-component system	Signal transduction				hypothetical protein CDS
**RPPX_RS26225**	72416	73261	reverse	68	operon_0039	6	fcluster_0039	10		two-component system	Signal transduction		pilD		hypothetical protein CDS
**RPPX_RS26230**	73258	73662	reverse	69	operon_0039	4	fcluster_0039	10		two-component system	Signal transduction		kdpE	regulator of ATP-driven potassium transport (or Kdp) system/	hypothetical protein CDS
**RPPX_RS26235**	74340	74864	reverse	70	operon_0040	4	fcluster_0041	11		hypothetical	Unknown				hypothetical protein CDS
**RPPX_RS26240**	75405	75665	reverse	71	operon_0041	NA	fcluster_0041	11		hypothetical	Unknown				hypothetical protein CDS
**RPPX_RS26245**	75731	76012	reverse	72	operon_0041	5	fcluster_0041	11		hypothetical	Unknown				hypothetical protein CDS
**RPPX_RS26250**	76161	78095	forward	73	operon_0042	6	fcluster_0042	12		Replication	Replication/partitioning	helicase			hypothetical protein CDS
**RPPX_RS26255**	78680	79075	forward	74	operon_0043	6	fcluster_0043	13		toluene stress response	Solvent stress	unknown		DUF2384 domain-containing protein	hypothetical protein CDS
**RPPX_RS26260**	79072	79596	forward	75	operon_0043	3	fcluster_0043	13		toluene stress response	Solvent stress	unknown		res domain containg protein; https://www.ncbi.nlm.nih.gov/Structure/cdd/cddsrv.cgi?ascbin=8&maxaln=10&seltype=2&uid=pfam08808	hypothetical protein CDS
**RPPX_RS26265**	79642	80394	reverse	76	operon_0044	NA	fcluster_0047	14		Stress response	General stress response	truncated	terZ		stress response protein TerZ CDS
**RPPX_RS26270**	80588	82102	forward	77	operon_0045	175	fcluster_0047	14		Stress response	General stress response			ISS12_Aachen	integrase CDS
**RPPX_RS26275**	82120	82875	forward	78	operon_0045	373	fcluster_0047	14		Stress response	General stress response			ISS12_Aachen	ATPase AAA CDS
**RPPX_RS26280**	82974	83495	reverse	79	operon_0046	NA	fcluster_0047	14		Stress response	General stress response		terZ		stress response protein TerZ CDS
**RPPX_RS26285**	85122	85313	forward	80	operon_0047	7	fcluster_0047	14		Stress response	General stress response				hypothetical protein CDS
**RPPX_RS26290**	85382	85636	forward	81	operon_0047	5	fcluster_0047	14		Stress response	General stress response				hypothetical protein CDS
**RPPX_RS26295**	85733	86878	reverse	82	operon_0048	414	fcluster_0048	15		glycolate degradation	Degradation	glycolate degradation	glxK	http://www.uniprot.org/uniprot/P77364	glycerate kinase CDS
**RPPX_RS26300**	87003	88088	reverse	83	operon_0048	6	fcluster_0048	15		glycolate degradation	Degradation	glycolate degradation	cdaR		CdaR family transcriptional regulator CDS
**RPPX_RS26305**	88301	88549	reverse	84	operon_0049	5	fcluster_0050	16		hypothetical	Unknown				hypothetical protein CDS
**RPPX_RS26310**	88562	89677	reverse	85	operon_0049	6	fcluster_0050	16		hypothetical	Unknown				hypothetical protein CDS
**RPPX_RS26315**	89865	91076	reverse	86	operon_0050	25	fcluster_0050	16		hypothetical	Unknown				hypothetical protein CDS
**RPPX_RS26320**	91086	92960	reverse	87	operon_0050	7	fcluster_0050	16		hypothetical	Unknown				hypothetical protein CDS

locus_tag	lo.min	lo.max	Direction	X	df_s12.mp.Operon	ID	f.cluster	clus.x	f.cluster_function	f.cluster_fmain	Gene_function	gene_name	Comments	cds
**RPPX_RS26325**	92969	93820	reverse	88	operon_0050	94	fcluster_0050	16	hypothetical	Unknown				hypothetical protein CDS
**RPPX_RS26330**	93847	95115	reverse	89	operon_0050	5	fcluster_0050	16	hypothetical	Unknown				hypothetical protein CDS
**RPPX_RS26335**	95357	95842	forward	90	operon_0051	1	fcluster_0051	17	heavy metal resistance	Heavy metal resistance				hypothetical protein CDS
**RPPX_RS26340**	95992	97251	forward	91	operon_0051	11	fcluster_0051	17	heavy metal resistance	Heavy metal resistance	tellerium resistance			hypothetical protein CDS
**RPPX_RS26345**	97361	98446	reverse	92	operon_0052	23	fcluster_0052	18	hypothetical	Unknown				hypothetical protein CDS
**RPPX_RS26350**	98761	99321	reverse	93	operon_0053	5	fcluster_0052	18	hypothetical	Unknown				phage protein CDS
**RPPX_RS26355**	99426	100451	reverse	94	operon_0053	9	fcluster_0052	18	hypothetical	Unknown				hypothetical protein CDS
**RPPX_RS26360**	100586	101164	reverse	95	operon_0053	587	fcluster_0053	19	heavy metal resistance	Heavy metal resistance		terE		tellurium resistance protein TerE CDS
**RPPX_RS26365**	101198	101776	reverse	96	operon_0053	688	fcluster_0053	19	heavy metal resistance	Heavy metal resistance		terD		tellurium resistance protein TerD CDS
**RPPX_RS26370**	101805	102839	reverse	97	operon_0053	247	fcluster_0053	19	heavy metal resistance	Heavy metal resistance		terC		tellurium resistance protein terC CDS
**RPPX_RS26375**	102851	103300	reverse	98	operon_0053	57	fcluster_0053	19	heavy metal resistance	Heavy metal resistance		terB		hypothetical protein CDS
**RPPX_RS26380**	103348	104529	reverse	99	operon_0053	41	fcluster_0053	19	heavy metal resistance	Heavy metal resistance		terA		tellurium resistance protein TerA CDS
**RPPX_RS26385**	104526	105119	reverse	100	operon_0053	303	fcluster_0053	19	heavy metal resistance	Heavy metal resistance		terZ		tellurium resistance protein TerZ CDS
**RPPX_RS26390**	105122	105850	reverse	101	operon_0053	414	fcluster_0053	19	heavy metal resistance	Heavy metal resistance		cicA		hypothetical protein CDS
**RPPX_RS26395**	105850	106797	reverse	102	operon_0053	363	fcluster_0053	19	heavy metal resistance	Heavy metal resistance				citrate lyase CDS
**RPPX_RS26400**	106797	107891	reverse	103	operon_0053	22	fcluster_0053	19	heavy metal resistance	Heavy metal resistance		stiP		ATP-binding protein CDS
**RPPX_RS26405**	107884	108642	reverse	104	operon_0053	171	fcluster_0053	19	heavy metal resistance	Heavy metal resistance				Trehalose-6-phosphatase CDS
**RPPX_RS26410**	108632	109759	reverse	105	operon_0053	321	fcluster_0053	19	heavy metal resistance	Heavy metal resistance				hypothetical protein CDS
**RPPX_RS26415**	109830	110969	reverse	106	operon_0053	162	fcluster_0053	19	heavy metal resistance	Heavy metal resistance				trypsin CDS
**RPPX_RS26420**	111045	112043	reverse	107	operon_0053	161	fcluster_0053	19	heavy metal resistance	Heavy metal resistance				carboxylate--amine ligase CDS
**RPPX_RS26425**	112275	112658	forward	108	operon_0054	4	fcluster_0060	20	DNA repair	Replication/partitioning				hypothetical protein CDS
**RPPX_RS26430**	112655	113227	reverse	109	operon_0055	6	fcluster_0060	20	DNA repair	Replication/partitioning				hypothetical protein CDS
**RPPX_RS26435**	113331	113645	reverse	110	operon_0055	7	fcluster_0060	20	DNA repair	Replication/partitioning				hypothetical protein CDS
**RPPX_RS26440**	113718	113942	reverse	111	operon_0055	5	fcluster_0060	20	DNA repair	Replication/partitioning				hypothetical protein CDS
**RPPX_RS26445**	113964	114215	forward	112	operon_0056	2	fcluster_0060	20	DNA repair	Replication/partitioning				hypothetical protein CDS
**RPPX_RS26450**	114219	114785	reverse	113	operon_0057	NA	fcluster_0060	20	DNA repair	Replication/partitioning				hypothetical protein CDS
**RPPX_RS26455**	114934	115593	reverse	114	operon_0057	1	fcluster_0060	20	DNA repair	Replication/partitioning		UvrD/Rep helica	P. aeruginosa	hypothetical protein CDS
**RPPX_RS26460**	115756	117270	forward	115	operon_0058	175	fcluster_0060	20	DNA repair	Replication/partitioning			ISS12_Aachen	integrase CDS
**RPPX_RS26465**	117288	118043	forward	116	operon_0058	373	fcluster_0060	20	DNA repair	Replication/partitioning			ISS12_Aachen	ATPase AAA CDS
**RPPX_RS26470**	118149	119861	reverse	117	operon_0059	NA	fcluster_0060	20	DNA repair	Replication/partitioning		UvrD/Rep helica	P. aeruginosa	hypothetical protein CDS
**RPPX_RS26475**	120158	120784	forward	118	operon_0060	5	fcluster_0060	20	DNA repair	Replication/partitioning	transcriptional regulator			transcriptional regulator CDS
**RPPX_RS26480**	120829	121155	reverse	119	operon_0061	5	fcluster_0063	21	hypothetical	Unknown				hypothetical protein CDS
**RPPX_RS26485**	121155	122267	reverse	120	operon_0061	10	fcluster_0063	21	hypothetical	Unknown				hypothetical protein CDS
**RPPX_RS26490**	122292	122915	reverse	121	operon_0061	5	fcluster_0063	21	hypothetical	Unknown				hypothetical protein CDS
**RPPX_RS26495**	122902	124554	reverse	122	operon_0061	6	fcluster_0063	21	hypothetical	Unknown				hypothetical protein CDS
**RPPX_RS26500**	124698	124955	reverse	123	operon_0061	3	fcluster_0063	21	hypothetical	Unknown				hypothetical protein CDS
**RPPX_RS26505**	125029	125355	reverse	124	operon_0061	30	fcluster_0063	21	hypothetical	Unknown				RNA-binding protein CDS
**RPPX_RS26510**	125539	126510	forward	125	operon_0062	4	fcluster_0063	21	hypothetical	Unknown				hypothetical protein CDS
**RPPX_RS26515**	126507	127070	forward	126	operon_0062	11	fcluster_0063	21	hypothetical	Unknown				hypothetical protein CDS
**RPPX_RS26520**	127074	128207	forward	127	operon_0062	6	fcluster_0063	21	hypothetical	Unknown				hypothetical protein CDS
**RPPX_RS26525**	128656	129024	reverse	128	operon_0063	6	fcluster_0063	21	hypothetical	Unknown				hypothetical protein CDS
**RPPX_RS26530**	129149	130183	forward	129	operon_0064	5	fcluster_0064	22	Stress response	General stress response				hypothetical protein CDS
**RPPX_RS26535**	130180	130443	forward	130	operon_0064	6	fcluster_0064	22	Stress response	General stress response				hypothetical protein CDS
**RPPX_RS26540**	130519	130962	forward	131	operon_0064	7	fcluster_0064	22	Stress response	General stress response	RNA chaparon	30S ribosomal pr	http://www.uniprot.org/uniprot/P0AG67	hypothetical protein CDS
**RPPX_RS26545**	131043	131642	forward	132	operon_0064	6	fcluster_0064	22	Stress response	General stress response	transcription factor	crp	http://www.uniprot.org/uniprot/P0ACJ8	hypothetical protein CDS
**RPPX_RS26550**	131632	131853	forward	133	operon_0064	12	fcluster_0064	22	Stress response	General stress response				hypothetical protein CDS
**RPPX_RS26555**	131850	132374	forward	134	operon_0064	6	fcluster_0064	22	Stress response	General stress response				hypothetical protein CDS
**RPPX_RS26560**	132384	135554	reverse	135	operon_0065	21	fcluster_0067	23	hypothetical	Unknown				hypothetical protein CDS
**RPPX_RS26565**	135790	136335	reverse	136	operon_0066	4	fcluster_0067	23	hypothetical	Unknown				hypothetical protein CDS
**RPPX_RS26570**	136411	139101	reverse	137	operon_0066	14	fcluster_0067	23	hypothetical	Unknown				hypothetical protein CDS
**RPPX_RS26575**	139348	140007	forward	138	operon_0067	6	fcluster_0067	23	hypothetical	Unknown				hypothetical protein CDS
**RPPX_RS26580**	140387	141403	reverse	139	operon_0068	12	fcluster_0070	24	Pilus assembly	Conjugation				hypothetical protein CDS
**RPPX_RS26585**	141412	142572	reverse	140	operon_0068	18	fcluster_0070	24	Pilus assembly	Conjugation				hypothetical protein CDS
**RPPX_RS26590**	142550	142867	reverse	141	operon_0068	5	fcluster_0070	24	Pilus assembly	Conjugation				hypothetical protein CDS
**RPPX_RS26595**	142870	143982	reverse	142	operon_0068	16	fcluster_0070	24	Pilus assembly	Conjugation				hypothetical protein CDS
**RPPX_RS26600**	144117	144878	reverse	143	operon_0068	4	fcluster_0070	24	Pilus assembly	Conjugation				hypothetical protein CDS
**RPPX_RS26605**	144875	145510	reverse	144	operon_0068	17	fcluster_0070	24	Pilus assembly	Conjugation				hypothetical protein CDS
**RPPX_RS26610**	145524	146705	reverse	145	operon_0068	10	fcluster_0070	24	Pilus assembly	Conjugation				SPASM domain-containing protein CDS
**RPPX_RS26615**	146702	148345	reverse	146	operon_0068	17	fcluster_0070	24	Pilus assembly	Conjugation				hypothetical protein CDS
**RPPX_RS26620**	148338	151805	reverse	147	operon_0068	6	fcluster_0070	24	Pilus assembly	Conjugation				hypothetical protein CDS
**RPPX_RS26625**	151796	153631	reverse	148	operon_0068	11	fcluster_0070	24	Pilus assembly	Conjugation				hypothetical protein CDS
**RPPX_RS26630**	153678	154787	reverse	149	operon_0068	NA	fcluster_0070	24	Pilus assembly	Conjugation				hypothetical protein CDS
**RPPX_RS26635**	154973	156487	forward	150	operon_0069	175	fcluster_0069	25	ISS12_ATCC	MGE			ISS12_ATCC	integrase CDS
**RPPX_RS26640**	156505	157260	forward	151	operon_0069	373	fcluster_0069	25	ISS12_ATCC	MGE			ISS12_ATCC	ATPase AAA CDS
**RPPX_RS26645**	157367	158005	reverse	152	operon_0070	NA	fcluster_0070	24	Pilus assembly	Conjugation				hypothetical protein CDS
**RPPX_RS26650**	157998	159062	reverse	153	operon_0070	18	fcluster_0070	24	Pilus assembly	Conjugation				hypothetical protein CDS
**RPPX_RS26655**	159103	160329	reverse	154	operon_0070	6	fcluster_0070	24	Pilus assembly	Conjugation				hypothetical protein CDS
**RPPX_RS26660**	160359	160733	reverse	155	operon_0070	7	fcluster_0070	24	Pilus assembly	Conjugation				hypothetical protein CDS
**RPPX_RS26665**	160771	161889	reverse	156	operon_0070	15	fcluster_0070	24	Pilus assembly	Conjugation				radical SAM protein CDS
**RPPX_RS26670**	161886	163028	reverse	157	operon_0070	16	fcluster_0070	24	Pilus assembly	Conjugation				transcriptional regulator CDS
**RPPX_RS26675**	163028	164110	reverse	158	operon_0070	9	fcluster_0070	24	Pilus assembly	Conjugation				SPASM domain-containing protein CDS
**RPPX_RS26680**	164224	165210	reverse	159	operon_0070	10	fcluster_0070	24	Pilus assembly	Conjugation				prepilin-type N-terminal cleavage/methylation domain-containing protein CDS
**RPPX_RS26685**	165224	165739	reverse	160	operon_0070	10	fcluster_0070	24	Pilus assembly	Conjugation				hypothetical protein CDS
**RPPX_RS26690**	165748	166911	reverse	161	operon_0070	12	fcluster_0070	24	Pilus assembly	Conjugation				hypothetical protein CDS
**RPPX_RS26695**	166922	167383	reverse	162	operon_0070	10	fcluster_0070	24	Pilus assembly	Conjugation				hypothetical protein CDS
**RPPX_RS26700**	167473	167934	reverse	163	operon_0070	10	fcluster_0070	24	Pilus assembly	Conjugation				hypothetical protein CDS
**RPPX_RS26705**	167980	169737	reverse	164	operon_0070	11	fcluster_0070	24	Pilus assembly	Conjugation				hypothetical protein CDS
**RPPX_RS26710**	169740	170399	reverse	165	operon_0070	6	fcluster_0070	24	Pilus assembly	Conjugation				hypothetical protein CDS
**RPPX_RS26715**	170402	170878	reverse	166	operon_0070	4	fcluster_0070	24	Pilus assembly	Conjugation				hypothetical protein CDS
**RPPX_RS26720**	170875	171810	reverse	167	operon_0070	6	fcluster_0070	24	Pilus assembly	Conjugation				hypothetical protein CDS
**RPPX_RS26725**	171810	173465	reverse	168	operon_0070	18	fcluster_0070	24	Pilus assembly	Conjugation				hypothetical protein CDS
**RPPX_RS26730**	173479	174366	reverse	169	operon_0070	10	fcluster_0070	24	Pilus assembly	Conjugation				hypothetical protein CDS
**RPPX_RS26735**	174494	175987	forward	170	operon_0071	6	fcluster_0071	26	hypothetical	Unknown				hypothetical protein CDS
**RPPX_RS26740**	176030	176503	reverse	171	operon_0072	13	fcluster_0072	27	hypothetical	Unknown				hypothetical protein CDS
**RPPX_RS26745**	176509	177801	reverse	172	operon_0072	8	fcluster_0072	27	hypothetical	Unknown				hypothetical protein CDS
**RPPX_RS26750**	177870	178559	reverse	173	operon_0072	14	fcluster_0072	27	hypothetical	Unknown				hypothetical protein CDS
**RPPX_RS26755**	178607	179512	reverse	174	operon_0072	6	fcluster_0072	27	hypothetical	Unknown				hypothetical protein CDS
**RPPX_RS26760**	179506	180306	reverse	175	operon_0072	6	fcluster_0072	27	hypothetical	Unknown				hypothetical protein CDS
**RPPX_RS26765**	180462	180842	reverse	176	operon_0073	6	fcluster_0073	28	hypothetical	Unknown				hypothetical protein CDS
**RPPX_RS26770**	180956	181549	reverse	177	operon_0073	6	fcluster_0073	28	hypothetical	Unknown				hypothetical protein CDS
**RPPX_RS26775**	181648	182418	reverse	178	operon_0073	6	fcluster_0073	28	hypothetical	Unknown				hypothetical protein CDS
**RPPX_RS26780**	182492	183757	reverse	179	operon_0073	6	fcluster_0073	28	hypothetical	Unknown				hypothetical protein CDS
**RPPX_RS26785**	183916	184230	reverse	180	operon_0074	6	fcluster_0074	29	hypothetical	Unknown				hypothetical protein CDS
**RPPX_RS26790**	184311	186365	reverse	181	operon_0074	6	fcluster_0074	29	hypothetical	Unknown				hypothetical protein CDS
**RPPX_RS26795**	186368	186583	reverse	182	operon_0074	4	fcluster_0074	29	hypothetical	Unknown				hypothetical protein CDS

locus_tag	lo.min	lo.max	Direction	X	df_s12.mp.Operon	ID	f.cluster	clus.x		f.cluster_function	f.cluster_fmain	Gene_function	gene_name	Comments	cds
**RPPX_RS26800**	186751	187008	reverse	183	operon_0075	8	fcluster_0075	30		hypothetical	Unknown				hypothetical protein CDS
**RPPX_RS26805**	187140	187961	reverse	184	operon_0075	6	fcluster_0075	30		hypothetical	Unknown				hypothetical protein CDS
**RPPX_RS26810**	187973	189094	reverse	185	operon_0075	6	fcluster_0075	30		hypothetical	Unknown				hypothetical protein CDS
**RPPX_RS26815**	189254	189691	reverse	186	operon_0076	6	fcluster_0076	31		hypothetical	Unknown				hypothetical protein CDS
**RPPX_RS26820**	189992	190288	reverse	187	operon_0077	5	fcluster_0077	32		hypothetical	Unknown				hypothetical protein CDS
**RPPX_RS26825**	190908	191405	reverse	188	operon_0078	6	fcluster_0078	33		hypothetical	Unknown				hypothetical protein CDS
**RPPX_RS26830**	191468	192418	reverse	189	operon_0078	8	fcluster_0078	33		hypothetical	Unknown				hypothetical protein CDS
**RPPX_RS26835**	193100	194338	reverse	190	operon_0079	6	fcluster_0079	34		hypothetical	Unknown				hypothetical protein CDS
**RPPX_RS26840**	195430	196629	reverse	191	operon_0080	6	fcluster_0081	35		partitioning	Replication/partitioning		parB		chromosome partitioning protein ParB CDS
**RPPX_RS26845**	196629	197528	reverse	192	operon_0080	4	fcluster_0081	35		partitioning	Replication/partitioning		parA		hypothetical protein CDS
**RPPX_RS26850**	197703	199145	reverse	193	operon_0081	4	fcluster_0081	35		partitioning	Replication/partitioning				hypothetical protein CDS
**RPPX_RS26855**	199265	200125	reverse	194	operon_0081	6	fcluster_0081	35		partitioning	Replication/partitioning		parB2		chromosome partitioning protein ParB CDS
**RPPX_RS26860**	200187	200825	reverse	195	operon_0081	10	fcluster_0081	35		partitioning	Replication/partitioning				hypothetical protein CDS
**RPPX_RS26865**	200980	202494	forward	196	operon_0082	175	fcluster_0081	35		ISS12_Aachen	MGE			ISS12_Aachen	integrase CDS
**RPPX_RS26870**	202512	203267	forward	197	operon_0082	373	fcluster_0081	35		ISS12_Aachen	MGE			ISS12_Aachen	ATPase AAA CDS
**RPPX_RS26875**	203571	203957	forward	198	operon_0083	21	fcluster_0083	36		chemotaxis cluster I	Signal transduction		cheZ		hypothetical protein CDS
**RPPX_RS26880**	203947	204435	forward	199	operon_0083	5	fcluster_0083	36		chemotaxis cluster I	Signal transduction		cheW		hypothetical protein CDS
**RPPX_RS26885**	204432	206456	forward	200	operon_0083	6	fcluster_0083	36		chemotaxis cluster I	Signal transduction		cheZ		hypothetical protein CDS
**RPPX_RS26890**	206453	207244	forward	201	operon_0083	14	fcluster_0083	36		chemotaxis cluster I	Signal transduction		cheR		hypothetical protein CDS
**RPPX_RS26895**	207247	213012	forward	202	operon_0083	8	fcluster_0083	36		chemotaxis cluster I	Signal transduction		cheA		hypothetical protein CDS
**RPPX_RS26900**	213009	213941	forward	203	operon_0083	7	fcluster_0083	36		chemotaxis cluster I	Signal transduction		cheB		hypothetical protein CDS
**RPPX_RS26905**	214151	214702	reverse	204	operon_0084	NA	fcluster_0087	37		MGE	MGE		RecA		recombinase RecA CDS
**RPPX_RS26910**	214843	216591	forward	205	operon_0085	7	fcluster_0087	37		MGE	MGE				hypothetical protein CDS
**RPPX_RS26915**	216920	218083	forward	206	operon_0086	3	fcluster_0087	37		MGE	MGE				hypothetical protein CDS
**RPPX_RS26920**	218214	219194	reverse	207	operon_0087	1751	fcluster_0087	37		ISP-pu21	MGE				transposase CDS
**RPPX_RS26925**	219298	219819	reverse	208	operon_0087	NA	fcluster_0096	38		DNA replication	Replication/partitioning		RecA		recombinase RecA CDS
**RPPX_RS26930**	219812	220159	reverse	209	operon_0087	NA	fcluster_0096	38		DNA replication	Replication/partitioning				hypothetical protein CDS
**RPPX_RS26935**	220222	221118	reverse	210	operon_0087	22	fcluster_0096	38		DNA replication	Replication/partitioning				hypothetical protein CDS
**RPPX_RS26940**	221268	221813	reverse	211	operon_0087	6	fcluster_0096	38		DNA replication	Replication/partitioning				hypothetical protein CDS
**RPPX_RS26945**	221932	222393	forward	212	operon_0088	3	fcluster_0096	38		DNA replication	Replication/partitioning				hypothetical protein CDS
**RPPX_RS26950**	222360	222713	reverse	213	operon_0089	3	fcluster_0096	38		DNA replication	Replication/partitioning				hypothetical protein CDS
**RPPX_RS26955**	222710	224278	reverse	214	operon_0089	6	fcluster_0096	38		DNA replication	Replication/partitioning				DNA-directed RNA polymerase sigma-70 factor CDS
**RPPX_RS26960**	224303	225409	reverse	215	operon_0089	6	fcluster_0096	38		DNA replication	Replication/partitioning		dnaN		DNA polymerase III subunit beta CDS
**RPPX_RS26965**	225580	226380	reverse	216	operon_0090	1	fcluster_0096	38		DNA replication	Replication/partitioning				hypothetical protein CDS
**RPPX_RS26970**	226566	227318	reverse	217	operon_0091	5	fcluster_0096	38		DNA replication	Replication/partitioning				hypothetical protein CDS
**RPPX_RS26975**	227305	228717	reverse	218	operon_0091	5	fcluster_0096	38		DNA replication	Replication/partitioning				hypothetical protein CDS
**RPPX_RS26980**	228874	229200	forward	219	operon_0092	2	fcluster_0096	38		DNA replication	Replication/partitioning				hypothetical protein CDS
**RPPX_RS26985**	229187	230008	reverse	220	operon_0093	21	fcluster_0096	38		DNA replication	Replication/partitioning			PRTRC system ThiF	hypothetical protein CDS
**RPPX_RS26990**	230012	230704	reverse	221	operon_0093	15	fcluster_0096	38		DNA replication	Replication/partitioning			PRTRC system protein B	hypothetical protein CDS
**RPPX_RS26995**	230714	232090	reverse	222	operon_0093	6	fcluster_0096	38		DNA replication	Replication/partitioning			PRTRC system protein F	hypothetical protein CDS
**RPPX_RS27000**	232196	232408	reverse	223	operon_0093	6	fcluster_0096	38		DNA replication	Replication/partitioning			PRTRC system protein C	PRTRC system protein C CDS
**RPPX_RS27005**	232420	232914	reverse	224	operon_0093	6	fcluster_0096	38		DNA replication	Replication/partitioning			PRTRC system protein E	hypothetical protein CDS
**RPPX_RS27010**	232948	234780	reverse	225	operon_0093	23	fcluster_0096	38		DNA replication	Replication/partitioning			PRTRC system ParB	hypothetical protein CDS
**RPPX_RS27015**	234928	235341	reverse	226	operon_0093	2	fcluster_0096	38		DNA replication	Replication/partitioning				hypothetical protein CDS
**RPPX_RS27020**	235726	236190	reverse	227	operon_0094	6	fcluster_0096	38		DNA replication	Replication/partitioning				hypothetical protein CDS
**RPPX_RS27025**	236199	236561	reverse	228	operon_0094	6	fcluster_0096	38		DNA replication	Replication/partitioning				hypothetical protein CDS
**RPPX_RS27030**	236558	237193	reverse	229	operon_0094	6	fcluster_0096	38		DNA replication	Replication/partitioning				hypothetical protein CDS
**RPPX_RS27035**	237382	237660	forward	230	operon_0095	5	fcluster_0096	38		DNA replication	Replication/partitioning				hypothetical protein CDS
**RPPX_RS27040**	237657	237989	reverse	231	operon_0096	2	fcluster_0096	38		DNA replication	Replication/partitioning				hypothetical protein CDS
**RPPX_RS27045**	237986	238384	reverse	232	operon_0096	6	fcluster_0096	38		DNA replication	Replication/partitioning				hypothetical protein CDS
**RPPX_RS27050**	240308	241822	forward	233	operon_0097	175	fcluster_0097	39		ISS12_ATCC	MGE				integrase CDS
**RPPX_RS27055**	241840	242595	forward	234	operon_0097	373	fcluster_0097	39		ISS12_ATCC	MGE				ATPase AAA CDS
**RPPX_RS27060**	242952	244139	reverse	235	operon_0098	9	fcluster_0101	40		DNA replication	Replication/partitioning		repA		hypothetical protein CDS
**RPPX_RS27065**	244178	244537	forward	236	operon_0099	6	fcluster_0101	40		DNA replication	Replication/partitioning				hypothetical protein CDS
**RPPX_RS27070**	244585	245019	reverse	237	operon_0100	5	fcluster_0101	40		DNA replication	Replication/partitioning				hypothetical protein CDS
**RPPX_RS27075**	245177	245434	forward	238	operon_0101	NA	fcluster_0101	40		DNA replication	Replication/partitioning		truncated csrA		carbon storage regulator CsrA CDS
**RPPX_RS27080**	245426	246181	reverse	239	operon_0102	373	fcluster_0102	41		ISS12_ATCC	MGE				ATPase AAA CDS
**RPPX_RS27085**	246202	247716	reverse	240	operon_0102	172	fcluster_0102	41		ISS12_ATCC	MGE				integrase CDS
**RPPX_RS27090**	247921	248256	forward	241	operon_0103	5	fcluster_0105	42		signal transduction	Signal transduction				hypothetical protein CDS
**RPPX_RS27095**	248281	248493	reverse	242	operon_0104	2	fcluster_0105	42		signal transduction	Signal transduction				hypothetical protein CDS
**RPPX_RS27100**	248875	249321	reverse	243	operon_0105	6	fcluster_0105	42		signal transduction	Signal transduction				hypothetical protein CDS
**RPPX_RS27105**	249318	250022	reverse	244	operon_0105	6	fcluster_0105	42		signal transduction	Signal transduction				hypothetical protein CDS
**RPPX_RS27110**	250029	250274	reverse	245	operon_0105	6	fcluster_0105	42		signal transduction	Signal transduction				hypothetical protein CDS
**RPPX_RS27115**	250320	251240	reverse	246	operon_0105	350	fcluster_0105	42		signal transduction	Signal transduction		rdgC		recombination-associated protein RdgC CDS
**RPPX_RS27120**	251353	251580	reverse	247	operon_0105	4	fcluster_0105	42		signal transduction	Signal transduction				hypothetical protein CDS
**RPPX_RS27125**	251593	252279	reverse	248	operon_0105	6	fcluster_0105	42		signal transduction	Signal transduction				hypothetical protein CDS
**RPPX_RS27130**	252291	252500	reverse	249	operon_0105	6	fcluster_0105	42		signal transduction	Signal transduction				hypothetical protein CDS
**RPPX_RS27135**	252553	252963	reverse	250	operon_0105	6	fcluster_0105	42		signal transduction	Signal transduction				hypothetical protein CDS
**RPPX_RS27140**	252960	253415	reverse	251	operon_0105	2	fcluster_0105	42		signal transduction	Signal transduction				hypothetical protein CDS
**RPPX_RS27145**	253412	254128	reverse	252	operon_0105	6	fcluster_0105	42		signal transduction	Signal transduction				hypothetical protein CDS
**RPPX_RS27150**	254244	254567	reverse	253	operon_0105	8	fcluster_0105	42		signal transduction	Signal transduction				hypothetical protein CDS
**RPPX_RS27155**	254641	255042	reverse	254	operon_0105	6	fcluster_0105	42		signal transduction	Signal transduction				hypothetical protein CDS
**RPPX_RS27160**	255134	255514	reverse	255	operon_0105	3	fcluster_0105	42		signal transduction	Signal transduction				hypothetical protein CDS
**RPPX_RS27165**	255645	255887	reverse	256	operon_0105	6	fcluster_0105	42		signal transduction	Signal transduction				hypothetical protein CDS
**RPPX_RS27170**	256035	256490	reverse	257	operon_0105	6	fcluster_0105	42		signal transduction	Signal transduction				hypothetical protein CDS
**RPPX_RS27175**	256566	256961	reverse	258	operon_0105	216	fcluster_0105	42		signal transduction	Signal transduction	Is involved in pilus biosynthesis and twitc	pilG	http://www.uniprot.org/uniprot/P46384	two-component system response regulator CDS
**RPPX_RS27180**	257281	257607	reverse	259	operon_0106	4	fcluster_0122	43		hypothetical	Unknown				hypothetical protein CDS
**RPPX_RS27185**	257758	258090	forward	260	operon_0107	4	fcluster_0122	43		hypothetical	Unknown				hypothetical protein CDS
**RPPX_RS27190**	258135	258707	reverse	261	operon_0108	7	fcluster_0122	43		hypothetical	Unknown				hypothetical protein CDS
**RPPX_RS27195**	258742	259860	reverse	262	operon_0108	6	fcluster_0122	43		hypothetical	Unknown				hypothetical protein CDS
**RPPX_RS27200**	259990	260517	reverse	263	operon_0108	5	fcluster_0122	43		hypothetical	Unknown				hypothetical protein CDS
**RPPX_RS27205**	260619	260855	reverse	264	operon_0108	3	fcluster_0122	43		hypothetical	Unknown				hypothetical protein CDS
**RPPX_RS27210**	260932	261996	reverse	265	operon_0108	12	fcluster_0122	43		hypothetical	Unknown				hypothetical protein CDS
**RPPX_RS27215**	261996	262319	reverse	266	operon_0108	5	fcluster_0122	43		hypothetical	Unknown				hypothetical protein CDS
**RPPX_RS27220**	262409	263050	reverse	267	operon_0108	4	fcluster_0122	43		hypothetical	Unknown				hypothetical protein CDS
**RPPX_RS27225**	263233	263637	reverse	268	operon_0109	4	fcluster_0122	43		hypothetical	Unknown				hypothetical protein CDS
**RPPX_RS27230**	263655	264215	reverse	269	operon_0109	3	fcluster_0122	43		hypothetical	Unknown				hypothetical protein CDS
**RPPX_RS27235**	264212	264553	reverse	270	operon_0109	5	fcluster_0122	43		hypothetical	Unknown				hypothetical protein CDS
**RPPX_RS27240**	264706	264921	forward	271	operon_0110	2	fcluster_0122	43		hypothetical	Unknown				hypothetical protein CDS
**RPPX_RS27245**	265298	265753	reverse	272	operon_0111	6	fcluster_0122	43		hypothetical	Unknown				hypothetical protein CDS
**RPPX_RS27250**	265937	266179	forward	273	operon_0112	2	fcluster_0122	43		hypothetical	Unknown				hypothetical protein CDS
**RPPX_RS27255**	266287	266814	reverse	274	operon_0113	6	fcluster_0122	43		hypothetical	Unknown				hypothetical protein CDS
**RPPX_RS27260**	268039	268680	reverse	275	operon_0114	4	fcluster_0122	43		hypothetical	Unknown				hypothetical protein CDS
**RPPX_RS27265**	269038	269457	forward	276	operon_0115	2	fcluster_0122	43		hypothetical	Unknown		ycbK		hypothetical protein CDS
**RPPX_RS27270**	269473	270135	reverse	277	operon_0116	5	fcluster_0122	43		hypothetical	Unknown		yiaD		hypothetical protein CDS
**RPPX_RS27275**	270211	270492	reverse	278	operon_0116	6	fcluster_0122	43		hypothetical	Unknown				hypothetical protein CDS

locus_tag	lo.min	lo.max	Direction	X	df_s12.mp.OperonID		f.cluster	clus.x		f.cluster_function	f.cluster_fmain	Gene_function	gene_name	Comments	cds
**RPPX_RS27280**	270754	271020	reverse	279	operon_0117	NA	fcluster_0122	43		hypothetical	Unknown				hypothetical protein CDS
**RPPX_RS27285**	271107	271970	reverse	280	operon_0117	44	fcluster_0122	43		hypothetical	Unknown				hypothetical protein CDS
**RPPX_RS27290**	272058	272366	forward	281	operon_0118	6	fcluster_0122	43		hypothetical	Unknown				hypothetical protein CDS
**RPPX_RS27295**	272376	272762	reverse	282	operon_0119	8	fcluster_0122	43		hypothetical	Unknown				hypothetical protein CDS
**RPPX_RS27300**	272755	273054	reverse	283	operon_0119	6	fcluster_0122	43		hypothetical	Unknown				hypothetical protein CDS
**RPPX_RS27305**	273047	273334	reverse	284	operon_0119	1	fcluster_0122	43		hypothetical	Unknown				hypothetical protein CDS
**RPPX_RS27310**	273770	274324	forward	285	operon_0120	4	fcluster_0122	43		hypothetical	Unknown				hypothetical protein CDS
**RPPX_RS27315**	274371	274568	reverse	286	operon_0121	6	fcluster_0122	43		hypothetical	Unknown				hypothetical protein CDS
**RPPX_RS27320**	274602	275267	reverse	287	operon_0121	6	fcluster_0122	43		hypothetical	Unknown				hypothetical protein CDS
**RPPX_RS27325**	275326	275826	reverse	288	operon_0121	5	fcluster_0122	43		hypothetical	Unknown				hypothetical protein CDS
**RPPX_RS27330**	275933	276211	reverse	289	operon_0121	1	fcluster_0122	43		hypothetical	Unknown				hypothetical protein CDS
**RPPX_RS27335**	276272	276841	reverse	290	operon_0121	4	fcluster_0122	43		hypothetical	Unknown				hypothetical protein CDS
**RPPX_RS27340**	276865	277329	reverse	291	operon_0121	4	fcluster_0122	43		hypothetical	Unknown				hypothetical protein CDS
**RPPX_RS27345**	277350	277730	reverse	292	operon_0121	4	fcluster_0122	43		hypothetical	Unknown				hypothetical protein CDS
**RPPX_RS27350**	277813	278094	reverse	293	operon_0121	6	fcluster_0122	43		hypothetical	Unknown				hypothetical protein CDS
**RPPX_RS27355**	278328	278918	reverse	294	operon_0122	4	fcluster_0122	43		hypothetical	Unknown				hypothetical protein CDS
**RPPX_RS27360**	279245	279499	reverse	295	operon_0123	2	fcluster_0123	44		aminoacid starvation	General stress response				hypothetical protein CDS
**RPPX_RS27365**	279541	280137	reverse	296	operon_0123	6	fcluster_0123	44		aminoacid starvation	General stress response		sspA	Forms an equimolar complex with the RNA poly	stringent starvation protein A CDS
**RPPX_RS27370**	280221	281333	reverse	297	operon_0123	7	fcluster_0123	44		aminoacid starvation	General stress response				hypothetical protein CDS
**RPPX_RS27375**	282000	282233	forward	298	operon_0124	1	fcluster_0126	45		hypothetical	Unknown				hypothetical protein CDS
**RPPX_RS27380**	282315	282545	forward	299	operon_0124	1	fcluster_0126	45		hypothetical	Unknown				hypothetical protein CDS
**RPPX_RS27385**	282836	284011	reverse	300	operon_0125	16	fcluster_0126	45		hypothetical	Unknown				hypothetical protein CDS
**RPPX_RS27390**	284084	284695	reverse	301	operon_0125	6	fcluster_0126	45		hypothetical	Unknown				hypothetical protein CDS
**RPPX_RS27395**	285385	286134	reverse	302	operon_0126	6	fcluster_0126	45		hypothetical	Unknown				hypothetical protein CDS
**RPPX_RS27400**	286229	286687	reverse	303	operon_0126	6	fcluster_0126	45	3	hypothetical	Unknown				hypothetical protein CDS
**RPPX_RS27405**	286768	287523	reverse	304	operon_0126	373	fcluster_0127	46	3	ISS12_ATCC	MGE				ATPase AAA CDS
**RPPX_RS27410**	287541	289055	reverse	305	operon_0126	175	fcluster_0127	46	3	ISS12_ATCC	MGE				integrase CDS
**RPPX_RS27415**	289324	289542	forward	306	operon_0127	1	fcluster_0130	47	3	heavy metal resistance	Heavy metal resistance		hypothetical		hypothetical protein CDS
**RPPX_RS27420**	289546	290475	reverse	307	operon_0128	108	fcluster_0130	47	3	heavy metal resistance	Heavy metal resistance		tnpR	Tn3-resolvase	resolvase CDS
**RPPX_RS27425**	290677	290916	forward	308	operon_0129	152	fcluster_0130	47	3	heavy metal resistance	Heavy metal resistance		metJ	methionine repressor-like protein	hypothetical protein CDS
**RPPX_RS27430**	290916	291326	forward	309	operon_0129	182	fcluster_0130	47	3	heavy metal resistance	Heavy metal resistance		pilT/PIN		twitching motility protein PilT CDS
**RPPX_RS27435**	291330	294323	forward	310	operon_0129	93	fcluster_0130	47	3	heavy metal resistance	Heavy metal resistance		tnpA	Tn3-transposase	transposase CDS
**RPPX_RS27440**	294336	294548	reverse	311	operon_0130	184	fcluster_0130	47	3	heavy metal resistance	Heavy metal resistance		hypothetical		hypothetical protein CDS
**RPPX_RS27445**	294556	294831	reverse	312	operon_0130	79	fcluster_0130	47	3	heavy metal resistance	Heavy metal resistance		merP2		mercury transporter CDS
**RPPX_RS27450**	295090	295359	forward	313	operon_0131	NA	fcluster_0130	47	3	heavy metal resistance	Heavy metal resistance		hypothetical	truncated due to insertion MGE	hypothetical protein CDS
**RPPX_RS27455**	295359	297017	reverse	314	operon_0132	16	fcluster_0130	47	3	heavy metal resistance	Heavy metal resistance		mcpT-2		methyl-accepting chemotaxis protein, McpT-2 CDS
**RPPX_RS27460**	297366	299045	forward	315	operon_0133	298	fcluster_0133	48	3	tn3	MGE		tniA		transposase CDS
**RPPX_RS27465**	299048	299956	forward	316	operon_0133	308	fcluster_0133	48	3	tn3	MGE		tniB		transposition protein TniB CDS
**RPPX_RS27470**	299953	301170	forward	317	operon_0133	65	fcluster_0133	48	3	tn3	MGE		tniQ		hypothetical protein CDS
**RPPX_RS27475**	301231	301845	forward	318	operon_0133	340	fcluster_0133	48	3	tn3	MGE		tniR		TniR protein CDS
**RPPX_RS27480**	301898	302134	reverse	319	operon_0134	264	fcluster_0135	49	3	heavy metal resistance	Heavy metal resistance		merE		mercury resistance protein CDS
**RPPX_RS27485**	302131	302496	reverse	320	operon_0134	279	fcluster_0135	49	3	heavy metal resistance	Heavy metal resistance		merD		transcriptional regulator CDS
**RPPX_RS27490**	302513	304159	reverse	321	operon_0134	865	fcluster_0135	49	3	heavy metal resistance	Heavy metal resistance		merA		mercuric reductase CDS
**RPPX_RS27495**	304156	304401	reverse	322	operon_0134	55	fcluster_0135	49	3	heavy metal resistance	Heavy metal resistance		merF		membrane protein CDS
**RPPX_RS27500**	304404	304679	reverse	323	operon_0134	326	fcluster_0135	49	3	heavy metal resistance	Heavy metal resistance		merP		mercuric transporter periplasmic component CDS
**RPPX_RS27505**	304695	305045	reverse	324	operon_0134	436	fcluster_0135	49	3	heavy metal resistance	Heavy metal resistance		merT		mercury transporter CDS
**RPPX_RS27510**	305117	305551	forward	325	operon_0135	464	fcluster_0135	49	3	heavy metal resistance	Heavy metal resistance		merR		MerR family transcriptional regulator CDS
**RPPX_RS27515**	305828	306388	forward	326	operon_0136	627	fcluster_0136	50	3	tn3	MGE				resolvase CDS
**RPPX_RS27520**	306392	309358	forward	327	operon_0136	225	fcluster_0136	50	3	tn3	MGE				transposase CDS
**RPPX_RS27525**	310554	312143	forward	328	operon_0137	4	fcluster_0137	51	3	degradation	Degradation			carboxylesterase; presence in VLB-120	carboxylesterase CDS
**RPPX_RS27530**	312466	313446	reverse	329	operon_0138	1751	fcluster_0138	52	3	ISPpu21	MGE			ISPpu21, absent in pSTY	transposase CDS
**RPPX_RS27535**	314567	314953	reverse	330	operon_0139	85	fcluster_0146	53		phenylacetate degradation	Degradation				HxlR family transcriptional regulator CDS
**RPPX_RS27540**	315078	315917	forward	331	operon_0140	24	fcluster_0146	53		phenylacetate degradation	Degradation				NAD(P)-dependent oxidoreductase CDS
**RPPX_RS27545**	316106	318160	reverse	332	operon_0141	435	fcluster_0146	53		phenylacetate degradation	Degradation		paaN		enoyl-CoA hydratase CDS
**RPPX_RS27550**	318219	319865	reverse	333	operon_0141	939	fcluster_0146	53		phenylacetate degradation	Degradation		paaL		actP CDS
**RPPX_RS27555**	319862	320170	reverse	334	operon_0141	25	fcluster_0146	53		phenylacetate degradation	Degradation		hypothetical		hypothetical protein CDS
**RPPX_RS27560**	320322	321395	reverse	335	operon_0142	152	fcluster_0146	53		phenylacetate degradation	Degradation		paaK		phenylacetic acid degradation protein CDS
**RPPX_RS27565**	321420	321953	reverse	336	operon_0142	289	fcluster_0146	53		phenylacetate degradation	Degradation		paaJ		phenylacetate-CoA oxygenase subunit PaaJ CDS
**RPPX_RS27570**	321940	322701	reverse	337	operon_0142	793	fcluster_0146	53		phenylacetate degradation	Degradation		paaI		phenylacetic acid degradation protein CDS
**RPPX_RS27575**	322712	322993	reverse	338	operon_0142	569	fcluster_0146	53		phenylacetate degradation	Degradation		paaH		paaB CDS
**RPPX_RS27580**	323017	324009	reverse	339	operon_0142	1606	fcluster_0146	53		phenylacetate degradation	Degradation		paaG		phenylacetate-CoA oxygenase subunit PaaA CDS
**RPPX_RS27585**	324493	325509	forward	340	operon_0143	47	fcluster_0146	53		phenylacetate degradation	Degradation		Integrase		transposase CDS
**RPPX_RS27590**	326120	327433	reverse	341	operon_0144	969	fcluster_0146	53		phenylacetate degradation	Degradation		paaF2		phenylacetate-CoA ligase CDS
**RPPX_RS27595**	327859	328782	reverse	342	operon_0145	192	fcluster_0146	53		phenylacetate degradation	Degradation		paaX		phenylacetic acid degradation operon negative regulatory protein PaaX CDS
**RPPX_RS27600**	328851	329444	reverse	343	operon_0145	1085	fcluster_0146	53		phenylacetate degradation	Degradation		paaY		phenylacetic acid degradation protein PaaY CDS
**RPPX_RS27605**	329431	331143	reverse	344	operon_0145	NA	fcluster_0146	53		phenylacetate degradation	Degradation		paaN2		aldehyde dehydrogenase CDS
**RPPX_RS27610**	331527	332300	forward	345	operon_0146	53	fcluster_0146	53		phenylacetate degradation	Degradation		paaA		2,3-dehydroadipyl-CoA hydratase CDS
**RPPX_RS27615**	332345	333136	forward	346	operon_0146	305	fcluster_0146	53		phenylacetate degradation	Degradation		paaB		enoyl-CoA hydratase CDS
**RPPX_RS27620**	333140	334663	forward	347	operon_0146	451	fcluster_0146	53		phenylacetate degradation	Degradation		paaC		3-hydroxyacyl-CoA dehydrogenase CDS
**RPPX_RS27625**	334650	335099	forward	348	operon_0146	245	fcluster_0146	53		phenylacetate degradation	Degradation		paaD		phenylacetic acid degradation protein CDS
**RPPX_RS27630**	335092	336297	forward	349	operon_0146	3376	fcluster_0146	53		phenylacetate degradation	Degradation		paaE		acetyl-CoA acetyltransferase CDS
**RPPX_RS27635**	336376	337689	forward	350	operon_0146	969	fcluster_0146	53		phenylacetate degradation	Degradation		paaF		phenylacetate-CoA ligase CDS
**RPPX_RS27640**	337976	340903	forward	351	operon_0147	6	fcluster_0148	54		Styrene degradation	Degradation		styS		nodulation protein NodV CDS
**RPPX_RS27645**	340900	341523	forward	352	operon_0147	9	fcluster_0148	54		Styrene degradation	Degradation		styR		DNA-binding response regulator CDS
**RPPX_RS27650**	341704	342951	forward	353	operon_0148	59	fcluster_0148	54		Styrene degradation	Degradation		styA		styrene monooxygenase StyA CDS
**RPPX_RS27655**	343006	343518	forward	354	operon_0148	13	fcluster_0148	54		Styrene degradation	Degradation		styB		hypothetical protein CDS
**RPPX_RS27660**	343588	344097	forward	355	operon_0148	27	fcluster_0148	54		Styrene degradation	Degradation		styC		hypothetical protein CDS
**RPPX_RS27665**	344112	345602	forward	356	operon_0148	970	fcluster_0148	54		Styrene degradation	Degradation		styD		aldehyde dehydrogenase CDS
**RPPX_RS27670**	345693	347063	forward	357	operon_0148	24	fcluster_0148	54		Styrene degradation	Degradation		styE		hypothetical protein CDS
**RPPX_RS27675**	347076	347393	forward	358	operon_0148	NA	fcluster_0148	54		Styrene degradation	Degradation				hypothetical protein CDS
**RPPX_RS29130**	347593	348804	forward	359	operon_0149	NA	fcluster_0149	55		hypothetical	Unknown				hypothetical protein CDS
**RPPX_RS29135**	348901	349869	reverse	360	operon_0150	NA	fcluster_0150	56		hypothetical	Unknown				hypothetical protein CDS
**RPPX_RS27690**	350084	351193	forward	361	operon_0151	204	fcluster_0151	57		hypothetical	Unknown				hypothetical protein CDS
**RPPX_RS27695**	351236	352441	forward	362	operon_0151	5	fcluster_0151	57		hypothetical	Unknown				hypothetical protein CDS
**RPPX_RS27700**	352461	353436	forward	363	operon_0151	NA	fcluster_0151	57		hypothetical	Unknown				hypothetical protein CDS
**RPPX_RS27705**	353461	354933	forward	364	operon_0151	619	fcluster_0151	57		hypothetical	Unknown				aldehyde dehydrogenase CDS
**RPPX_RS27710**	355217	356851	forward	365	operon_0152	7	fcluster_0152	58		hypothetical	Unknown				methyl-accepting chemotaxis protein CDS
**RPPX_RS27715**	357018	357599	reverse	366	operon_0153	11	fcluster_0154	59		protocatechuate degradation	Degradation				TetR family transcriptional regulator CDS
**RPPX_RS27720**	357992	358288	forward	367	operon_0154	23	fcluster_0154	59		protocatechuate degradation	Degradation				extradiol ring-cleavage dioxygenase CDS
**RPPX_RS27725**	358293	359138	forward	368	operon_0154	29	fcluster_0154	59		protocatechuate degradation	Degradation				protocatechuate 4,5-dioxygenase subunit beta CDS
**RPPX_RS27730**	359194	359916	forward	369	operon_0154	NA	fcluster_0154	59		protocatechuate degradation	Degradation	Aromatic ring hydroxylating diox	Aromatic ring hydroxylating dioxygenase, alpha subu		ribosomal subunit interface protein CDS
**RPPX_RS27735**	360007	360405	reverse	370	operon_0155	464	fcluster_0155	60		heavy metal resistance	Heavy metal resistance		merR		MerR family transcriptional regulator CDS
**RPPX_RS27740**	360480	360830	forward	371	operon_0156	92	fcluster_0156	61		heavy metal resistance	Heavy metal resistance		merT		mercury transporter MerT CDS
**RPPX_RS27745**	360843	361118	forward	372	operon_0156	79	fcluster_0156	61		heavy metal resistance	Heavy metal resistance		merP		mercury transporter CDS
**RPPX_RS27750**	361126	361338	forward	373	operon_0156	184	fcluster_0156	61		heavy metal resistance	Heavy metal resistance		hypothetical		hypothetical protein CDS
**RPPX_RS27755**	361351	364344	reverse	374	operon_0157	93	fcluster_0157	62		tn3	MGE				transposase CDS

locus_tag	lo.min	lo.max	Direction	X	df_s12.mp.OperonID		f.cluster	clus.x		f.cluster_function	f.cluster_fmain	Gene_function	gene_name	Comments	cds
**RPPX_RS27760**	364348	364758	reverse	375	operon_0157	182	fcluster_0157	62		tn3	MGE				twitching motility protein PilT CDS
**RPPX_RS27765**	364758	364997	reverse	376	operon_0157	152	fcluster_0157	62		tn3	MGE				hypothetical protein CDS
**RPPX_RS27770**	365199	366128	forward	377	operon_0158	108	fcluster_0158	63		tn3	MGE				resolvase CDS
**RPPX_RS27775**	366271	366600	forward	378	operon_0158	2	fcluster_0158	63		tn3	MGE				hypothetical protein CDS
**RPPX_RS27780**	366597	367343	forward	379	operon_0158	25	fcluster_0158	63		tn3	MGE				transposase CDS
**RPPX_RS27785**	368101	369456	reverse	380	operon_0159	6	fcluster_0162	64		hypothetical	Unknown				hypothetical protein CDS
**RPPX_RS27790**	369587	370555	reverse	381	operon_0159	8	fcluster_0162	64		hypothetical	Unknown				methyltransferase CDS
**RPPX_RS27795**	370720	371433	reverse	382	operon_0160	9	fcluster_0162	64		hypothetical	Unknown				hypothetical protein CDS
**RPPX_RS27800**	371501	371941	reverse	383	operon_0160	20	fcluster_0162	64		hypothetical	Unknown				hypothetical protein CDS
**RPPX_RS27805**	371938	372117	reverse	384	operon_0160	2	fcluster_0162	64		hypothetical	Unknown				hypothetical protein CDS
**RPPX_RS27810**	372216	372644	reverse	385	operon_0160	6	fcluster_0162	64		hypothetical	Unknown				hypothetical protein CDS
**RPPX_RS27815**	372695	373711	reverse	386	operon_0160	447	fcluster_0162	64		hypothetical	Unknown		ndpA		nucleoid-associated protein CDS
**RPPX_RS27820**	373797	374525	reverse	387	operon_0160	6	fcluster_0162	64		hypothetical	Unknown				hypothetical protein CDS
**RPPX_RS27825**	374701	375150	reverse	388	operon_0161	6	fcluster_0162	64		hypothetical	Unknown				hypothetical protein CDS
**RPPX_RS27830**	375321	375623	forward	389	operon_0162	4	fcluster_0162	64		hypothetical	Unknown				hypothetical protein CDS
**RPPX_RS27835**	375678	376268	reverse	390	operon_0163	6	fcluster_0165	65		hypothetical	Unknown				hypothetical protein CDS
**RPPX_RS27840**	376412	377044	reverse	391	operon_0163	7	fcluster_0165	65		hypothetical	Unknown				hypothetical protein CDS
**RPPX_RS27845**	377409	378284	reverse	392	operon_0164	6	fcluster_0165	65		hypothetical	Unknown				hypothetical protein CDS
**RPPX_RS27850**	378804	379289	forward	393	operon_0165	1	fcluster_0165	65		hypothetical	Unknown				hypothetical protein CDS
**RPPX_RS27855**	379355	381244	reverse	394	operon_0166	6	fcluster_0167	66		hypothetical	Unknown		ftsH		ATP-dependent metalloprotease CDS
**RPPX_RS27860**	381351	381971	reverse	395	operon_0166	6	fcluster_0167	66		hypothetical	Unknown				hypothetical protein CDS
**RPPX_RS27865**	382133	382675	reverse	396	operon_0167	9	fcluster_0167	66		hypothetical	Unknown				hypothetical protein CDS
**RPPX_RS27870**	382778	383005	reverse	397	operon_0167	6	fcluster_0167	66		hypothetical	Unknown				hypothetical protein CDS
**RPPX_RS27875**	383015	383344	reverse	398	operon_0167	6	fcluster_0167	66		hypothetical	Unknown				hypothetical protein CDS
**RPPX_RS27880**	383449	384105	reverse	399	operon_0167	93	fcluster_0167	66		hypothetical	Unknown	Hisitidine kinase			hypothetical protein CDS
**RPPX_RS27885**	384201	384536	reverse	400	operon_0167	9	fcluster_0167	66		hypothetical	Unknown				hypothetical protein CDS
**RPPX_RS27890**	384546	384995	reverse	401	operon_0167	8	fcluster_0167	66		hypothetical	Unknown				hypothetical protein CDS
**RPPX_RS27895**	385098	385430	reverse	402	operon_0167	34	fcluster_0167	66		hypothetical	Unknown				DNA-binding protein CDS
**RPPX_RS27900**	385589	385978	reverse	403	operon_0168	13	fcluster_0170	67		hypothetical	Unknown				hypothetical protein CDS
**RPPX_RS27905**	386002	387816	reverse	404	operon_0168	19	fcluster_0170	67		hypothetical	Unknown				hypothetical protein CDS
**RPPX_RS27910**	388049	388474	reverse	405	operon_0169	10	fcluster_0170	67		hypothetical	Unknown				hypothetical protein CDS
**RPPX_RS27915**	388517	388750	reverse	406	operon_0169	5	fcluster_0170	67		hypothetical	Unknown				hypothetical protein CDS
**RPPX_RS27920**	388844	389335	reverse	407	operon_0169	5	fcluster_0170	67		hypothetical	Unknown				hypothetical protein CDS
**RPPX_RS27925**	389384	390043	reverse	408	operon_0169	6	fcluster_0170	67		hypothetical	Unknown				hypothetical protein CDS
**RPPX_RS27930**	390218	390769	forward	409	operon_0170	5	fcluster_0170	67		hypothetical	Unknown				hypothetical protein CDS
**RPPX_RS27935**	391025	391402	reverse	410	operon_0171	5	fcluster_0174	68		hypothetical	Unknown				hypothetical protein CDS
**RPPX_RS27940**	391435	391695	reverse	411	operon_0171	4	fcluster_0174	68		hypothetical	Unknown				hypothetical protein CDS
**RPPX_RS27945**	391822	392232	forward	412	operon_0172	1	fcluster_0174	68		hypothetical	Unknown				hypothetical protein CDS
**RPPX_RS27950**	392294	392617	reverse	413	operon_0173	4	fcluster_0174	68		hypothetical	Unknown				hypothetical protein CDS
**RPPX_RS27955**	392885	393175	forward	414	operon_0174	1	fcluster_0174	68		hypothetical	Unknown				hypothetical protein CDS
**RPPX_RS27960**	393893	394213	reverse	415	operon_0175	8	fcluster_0178	69		RND solvent efflux pump	Solvent stress				hypothetical protein CDS
**RPPX_RS27965**	394322	395734	reverse	416	operon_0175	1328	fcluster_0178	69		RND solvent efflux pump	Solvent stress		srpC		solvent efflux pump outer membrane protein SrpC CDS
**RPPX_RS27970**	395724	398873	reverse	417	operon_0175	4076	fcluster_0178	69		RND solvent efflux pump	Solvent stress		srpB		multidrug efflux RND transporter permease subunit CDS
**RPPX_RS27975**	398887	399186	reverse	418	operon_0175	1	fcluster_0178	69		RND solvent efflux pump	Solvent stress		SrpA		hypothetical protein CDS
**RPPX_RS27980**	399314	400828	forward	419	operon_0176	175	fcluster_0178	69		RND solvent efflux pump	Solvent stress				integrase CDS
**RPPX_RS27985**	400846	401601	forward	420	operon_0176	373	fcluster_0178	69		RND solvent efflux pump	Solvent stress				ATPase AAA CDS
**RPPX_RS27990**	401706	402665	reverse	421	operon_0177	NA	fcluster_0178	69		RND solvent efflux pump	Solvent stress		SrpA		antibiotic transporter CDS
**RPPX_RS27995**	402862	403641	forward	422	operon_0178	20	fcluster_0178	69		RND solvent efflux pump	Solvent stress		srpS		transcriptional regulator CDS
**RPPX_RS28000**	403647	404288	forward	423	operon_0178	14	fcluster_0178	69		RND solvent efflux pump	Solvent stress		srpR		transcriptional regulator CDS
**RPPX_RS28005**	404305	405948	reverse	424	operon_0179	110	fcluster_0183	70		3-phenylpropanoate degradation	Degradation		als		acetolactate synthase CDS
**RPPX_RS28010**	406147	407895	reverse	425	operon_0180	94	fcluster_0183	70		3-phenylpropanoate degradation	Degradation		tfdB	2,4-dichlorophenol 6-monooxygenase	hypothetical protein CDS
**RPPX_RS28015**	408309	409577	reverse	426	operon_0181	211	fcluster_0183	70		3-phenylpropanoate degradation	Degradation		nicP	Porin-like protein NicP precursor	porin CDS
**RPPX_RS28020**	409619	410836	reverse	427	operon_0181	4	fcluster_0183	70		3-phenylpropanoate degradation	Degradation		pcaK	4-hydroxybenzoate transporter PcaK	MFS transporter CDS
**RPPX_RS28025**	411102	412112	reverse	428	operon_0182	46	fcluster_0183	70		3-phenylpropanoate degradation	Degradation		mhpE	-336.6666667	4-hydroxy-2-oxovalerate aldolase CDS
**RPPX_RS28030**	412124	413062	reverse	429	operon_0182	2397	fcluster_0183	70		3-phenylpropanoate degradation	Degradation		mhpF	-312.6666667	acetaldehyde dehydrogenase CDS
**RPPX_RS28035**	413076	413870	reverse	430	operon_0182	293	fcluster_0183	70		3-phenylpropanoate degradation	Degradation		mhpD	-264.6666667	2-keto-4-pentenoate hydratase CDS
**RPPX_RS28040**	413882	414742	reverse	431	operon_0182	8	fcluster_0183	70		3-phenylpropanoate degradation	Degradation		mhpC	-286.6666667	2-hydroxy-6-oxo-6-phenylhexa-2,4-dienoate hydrolase CDS
**RPPX_RS28045**	414763	415707	reverse	432	operon_0182	5	fcluster_0183	70		3-phenylpropanoate degradation	Degradation		mhpB	-314.6666667	mhpB CDS
**RPPX_RS28050**	416033	416698	forward	433	operon_0183	5	fcluster_0183	70		3-phenylpropanoate degradation	Degradation		nanR	transcriptional regulator NanR	GntR family transcriptional regulator CDS
**RPPX_RS28055**	416693	417451	reverse	434	operon_0184	14	fcluster_0183	70		3-phenylpropanoate degradation	Degradation			hypothetical	hypothetical protein CDS
**RPPX_RS28060**	417704	417931	forward	435	operon_0185	3	fcluster_0183	70		3-phenylpropanoate degradation	Degradation	hypothetical		Cupin domain protein	hypothetical protein CDS
**RPPX_RS28065**	418217	419323	reverse	436	operon_0186	16	fcluster_0183	70		3-phenylpropanoate degradation	Degradation		adhT	adhT CDS	alcohol dehydrogenase CDS
**RPPX_RS28070**	419878	420234	forward	437	operon_0187	81	fcluster_0187	71		IS5	MGE				transposase CDS
**RPPX_RS28075**	420778	420990	reverse	438	operon_0188	2	fcluster_0188	72		hypothetical	Unknown				hypothetical protein CDS
**RPPX_RS28080**	421105	421428	reverse	439	operon_0188	5	fcluster_0188	72		hypothetical	Unknown				hypothetical protein CDS
**RPPX_RS28085**	421428	421844	reverse	440	operon_0188	7	fcluster_0188	72		hypothetical	Unknown				hypothetical protein CDS
**RPPX_RS28090**	422064	423008	reverse	441	operon_0189	5	fcluster_0189	73		hypothetical	Unknown				hypothetical protein CDS
**RPPX_RS28095**	423098	423535	reverse	442	operon_0189	5	fcluster_0189	73		hypothetical	Unknown				hypothetical protein CDS
**RPPX_RS28100**	423623	424252	reverse	443	operon_0189	5	fcluster_0189	73		hypothetical	Unknown				hypothetical protein CDS
**RPPX_RS28105**	424462	425967	forward	444	operon_0190	68	fcluster_0190	74		Cell division	Replication/partitioning	cell devision and folate metabolism	fic		cell division protein Fic CDS
**RPPX_RS28110**	425970	426518	reverse	445	operon_0191	5	fcluster_0193	75		hypothetical	Unknown				hypothetical protein CDS
**RPPX_RS28115**	426558	427505	reverse	446	operon_0191	7	fcluster_0193	75		hypothetical	Unknown				hypothetical protein CDS
**RPPX_RS28120**	427595	427843	reverse	447	operon_0191	6	fcluster_0193	75		hypothetical	Unknown				hypothetical protein CDS
**RPPX_RS28125**	427870	428091	reverse	448	operon_0191	6	fcluster_0193	75		hypothetical	Unknown				hypothetical protein CDS
**RPPX_RS28130**	428182	428523	reverse	449	operon_0191	5	fcluster_0193	75		hypothetical	Unknown				hypothetical protein CDS
**RPPX_RS28135**	428793	429161	reverse	450	operon_0192	4	fcluster_0193	75		hypothetical	Unknown				hypothetical protein CDS
**RPPX_RS28140**	429185	429679	reverse	451	operon_0192	5	fcluster_0193	75		hypothetical	Unknown				hypothetical protein CDS
**RPPX_RS28145**	429831	430322	reverse	452	operon_0193	4	fcluster_0193	75		hypothetical	Unknown				hypothetical protein CDS
**RPPX_RS28150**	430376	430714	reverse	453	operon_0193	3	fcluster_0193	75		hypothetical	Unknown				hypothetical protein CDS
**RPPX_RS28155**	430924	431625	forward	454	operon_0194	10	fcluster_0201	76		hypothetical	Unknown	ATP-dependent RNA helicase			hypothetical protein CDS
**RPPX_RS28160**	431705	432433	reverse	455	operon_0195	5	fcluster_0201	76		hypothetical	Unknown				hypothetical protein CDS
**RPPX_RS28165**	433044	433223	reverse	456	operon_0196	5	fcluster_0201	76		hypothetical	Unknown				hypothetical protein CDS
**RPPX_RS28170**	433469	433861	reverse	457	operon_0197	9	fcluster_0201	76		hypothetical	Unknown				hypothetical protein CDS
**RPPX_RS28175**	433867	434244	reverse	458	operon_0197	5	fcluster_0201	76		hypothetical	Unknown				hypothetical protein CDS
**RPPX_RS28180**	434282	434704	reverse	459	operon_0197	5	fcluster_0201	76		hypothetical	Unknown				hypothetical protein CDS
**RPPX_RS28185**	434850	435146	reverse	460	operon_0197	6	fcluster_0201	76		hypothetical	Unknown				hypothetical protein CDS
**RPPX_RS28190**	435173	435403	reverse	461	operon_0197	3	fcluster_0201	76		hypothetical	Unknown				hypothetical protein CDS
**RPPX_RS28195**	435460	435672	reverse	462	operon_0197	3	fcluster_0201	76		hypothetical	Unknown				hypothetical protein CDS
**RPPX_RS28200**	435960	436145	reverse	463	operon_0198	7	fcluster_0201	76		hypothetical	Unknown				hypothetical protein CDS
**RPPX_RS28205**	436156	436446	reverse	464	operon_0198	8	fcluster_0201	76		hypothetical	Unknown				hypothetical protein CDS
**RPPX_RS28210**	436920	437297	reverse	465	operon_0199	6	fcluster_0201	76		hypothetical	Unknown				hypothetical protein CDS
**RPPX_RS28215**	437463	437687	reverse	466	operon_0200	4	fcluster_0201	76		hypothetical	Unknown				hypothetical protein CDS
**RPPX_RS28220**	437991	438281	reverse	467	operon_0201	6	fcluster_0201	76		hypothetical	Unknown				hypothetical protein CDS
**RPPX_RS28225**	438274	438858	reverse	468	operon_0201	6	fcluster_0201	76		hypothetical	Unknown				hypothetical protein CDS
**RPPX_RS28230**	438851	439066	reverse	469	operon_0201	5	fcluster_0201	76		hypothetical	Unknown				hypothetical protein CDS
**RPPX_RS28235**	439063	441309	reverse	470	operon_0201	6	fcluster_0201	76		hypothetical	Unknown				hypothetical protein CDS

locus_tag	lo.min	lo.max	Direction	X	df_s12.mp.OperonID		f.cluster	clus.x		f.cluster_function	f.cluster_fmain	Gene_function	gene_name	Comments	cds
**RPPX_RS28240**	441354	441611	reverse	471	operon_0201	6	fcluster_0201	76		hypothetical	Unknown				hypothetical protein CDS
**RPPX_RS28245**	441744	442220	reverse	472	operon_0201	NA	fcluster_0201	76		hypothetical	Unknown				hypothetical protein CDS
**RPPX_RS28250**	442213	442923	reverse	473	operon_0201	6	fcluster_0201	76		hypothetical	Unknown				hypothetical protein CDS
**RPPX_RS28255**	443012	444109	reverse	474	operon_0201	3	fcluster_0201	76		hypothetical	Unknown				hypothetical protein CDS
**RPPX_RS28260**	444133	444888	reverse	475	operon_0201	3	fcluster_0201	76		hypothetical	Unknown				hypothetical protein CDS
**RPPX_RS28265**	445296	445535	reverse	476	operon_0202	3	fcluster_0209	77		hypothetical	Unknown				hypothetical protein CDS
**RPPX_RS28270**	445547	445963	reverse	477	operon_0202	3	fcluster_0209	77		hypothetical	Unknown				hypothetical protein CDS
**RPPX_RS28275**	446048	446275	reverse	478	operon_0202	3	fcluster_0209	77		hypothetical	Unknown				hypothetical protein CDS
**RPPX_RS28280**	446328	446804	reverse	479	operon_0202	4	fcluster_0209	77		hypothetical	Unknown				hypothetical protein CDS
**RPPX_RS28285**	446861	447292	reverse	480	operon_0202	5	fcluster_0209	77		hypothetical	Unknown				hypothetical protein CDS
**RPPX_RS28290**	447320	448141	reverse	481	operon_0202	16	fcluster_0209	77		hypothetical	Unknown				DNA methyltransferase CDS
**RPPX_RS28295**	448294	449133	forward	482	operon_0203	5	fcluster_0209	77		hypothetical	Unknown				hypothetical protein CDS
**RPPX_RS28300**	449247	449624	reverse	483	operon_0204	11	fcluster_0209	77		hypothetical	Unknown				hypothetical protein CDS
**RPPX_RS28305**	449638	449904	reverse	484	operon_0204	3	fcluster_0209	77		hypothetical	Unknown				hypothetical protein CDS
**RPPX_RS28310**	449901	450377	reverse	485	operon_0204	2	fcluster_0209	77		hypothetical	Unknown				hypothetical protein CDS
**RPPX_RS28315**	450521	450808	reverse	486	operon_0204	2	fcluster_0209	77		hypothetical	Unknown				hypothetical protein CDS
**RPPX_RS28320**	450846	451208	reverse	487	operon_0204	5	fcluster_0209	77		hypothetical	Unknown				hypothetical protein CDS
**RPPX_RS28325**	451243	451428	reverse	488	operon_0204	5	fcluster_0209	77		hypothetical	Unknown				hypothetical protein CDS
**RPPX_RS28330**	451480	451737	reverse	489	operon_0204	9	fcluster_0209	77		hypothetical	Unknown				hypothetical protein CDS
**RPPX_RS28335**	451750	451959	reverse	490	operon_0204	3	fcluster_0209	77		hypothetical	Unknown				hypothetical protein CDS
**RPPX_RS28340**	452036	453031	reverse	491	operon_0204	6	fcluster_0209	77		hypothetical	Unknown				hypothetical protein CDS
**RPPX_RS28345**	453221	453601	reverse	492	operon_0205	6	fcluster_0209	77		hypothetical	Unknown				hypothetical protein CDS
**RPPX_RS28350**	453747	454184	reverse	493	operon_0205	5	fcluster_0209	77		hypothetical	Unknown				hypothetical protein CDS
**RPPX_RS28355**	454221	454643	reverse	494	operon_0205	4	fcluster_0209	77		hypothetical	Unknown				hypothetical protein CDS
**RPPX_RS28360**	454855	455358	reverse	495	operon_0206	5	fcluster_0209	77		hypothetical	Unknown				hypothetical protein CDS
**RPPX_RS28365**	455423	455916	reverse	496	operon_0206	NA	fcluster_0209	77		hypothetical	Unknown				hypothetical protein CDS
**RPPX_RS28370**	455981	456550	reverse	497	operon_0206	5	fcluster_0209	77		hypothetical	Unknown	Oligoribonuclease	orn		hypothetical protein CDS
**RPPX_RS28375**	456631	457035	reverse	498	operon_0206	4	fcluster_0209	77		hypothetical	Unknown				hypothetical protein CDS
**RPPX_RS28380**	457356	457757	reverse	499	operon_0207	2	fcluster_0209	77		hypothetical	Unknown				hypothetical protein CDS
**RPPX_RS28385**	457758	458042	reverse	500	operon_0207	2	fcluster_0209	77		hypothetical	Unknown				hypothetical protein CDS
**RPPX_RS28390**	458039	458398	reverse	501	operon_0207	5	fcluster_0209	77		hypothetical	Unknown				hypothetical protein CDS
**RPPX_RS28395**	458426	458890	reverse	502	operon_0207	6	fcluster_0209	77		hypothetical	Unknown				hypothetical protein CDS
**RPPX_RS28400**	459112	459561	reverse	503	operon_0208	10	fcluster_0209	77		hypothetical	Unknown				hypothetical protein CDS
**RPPX_RS28405**	459651	460043	reverse	504	operon_0208	4	fcluster_0209	77		hypothetical	Unknown				hypothetical protein CDS
**RPPX_RS28410**	460095	460739	reverse	505	operon_0208	8	fcluster_0209	77		hypothetical	Unknown				hypothetical protein CDS
**RPPX_RS28415**	461029	461403	reverse	506	operon_0209	5	fcluster_0209	77		hypothetical	Unknown				hypothetical protein CDS
**RPPX_RS28420**	461552	461914	reverse	507	operon_0209	5	fcluster_0209	77		hypothetical	Unknown				hypothetical protein CDS
**RPPX_RS28425**	462030	462485	reverse	508	operon_0209	4	fcluster_0209	77		hypothetical	Unknown				hypothetical protein CDS
**RPPX_RS28430**	462494	462931	reverse	509	operon_0209	5	fcluster_0209	77		hypothetical	Unknown				hypothetical protein CDS
**RPPX_RS28435**	462963	463265	reverse	510	operon_0209	5	fcluster_0209	77		hypothetical	Unknown				hypothetical protein CDS
**RPPX_RS28440**	463367	463684	reverse	511	operon_0209	NA	fcluster_0209	77		hypothetical	Unknown		truncated protein		hypothetical protein CDS
**RPPX_RS28445**	463888	465402	forward	512	operon_0210	175	fcluster_0209	77		hypothetical	Unknown	ISS12_Aachen			integrase CDS
**RPPX_RS28450**	465420	466175	forward	513	operon_0210	373	fcluster_0209	77		hypothetical	Unknown	ISS12_Aachen			ATPase AAA CDS
**RPPX_RS28455**	466330	466533	forward	514	operon_0211	1	fcluster_0209	77		hypothetical	Unknown		truncated protein		hypothetical protein CDS
**RPPX_RS28460**	466866	467378	reverse	515	operon_0212	5	fcluster_0209	77		hypothetical	Unknown				hypothetical protein CDS
**RPPX_RS28465**	467491	468300	forward	516	operon_0213	4	fcluster_0209	77		hypothetical	Unknown				hypothetical protein CDS
**RPPX_RS28470**	468270	469385	reverse	517	operon_0214	5	fcluster_0209	77		hypothetical	Unknown				hypothetical protein CDS
**RPPX_RS28475**	469599	471128	forward	518	operon_0215	10	fcluster_0209	77		hypothetical	Unknown				two-component system diguanylate cyclase response regulator CDS
**RPPX_RS28480**	471182	472414	reverse	519	operon_0216	6	fcluster_0220	78		hypothetical	Unknown	transmembrane protein		plasmid specific, in putida and Aeuriginosa	hypothetical protein CDS
**RPPX_RS28485**	472428	473003	reverse	520	operon_0216	2	fcluster_0220	78		hypothetical	Unknown	plasmid specific, in putida and Aeuriginosa		plasmid specific, in putida and Aeuriginosa	hypothetical protein CDS
**RPPX_RS28490**	472996	473313	reverse	521	operon_0216	18	fcluster_0220	78		hypothetical	Unknown	plasmid specific, in putida and Aeuriginosa		plasmid specific, in putida and Aeuriginosa	hypothetical protein CDS
**RPPX_RS28495**	473306	474676	reverse	522	operon_0216	11	fcluster_0220	78		hypothetical	Unknown	plasmid specific, in putida and Aeuriginosa		plasmid specific, in putida and Aeuriginosa	hypothetical protein CDS
**RPPX_RS28500**	474658	475389	reverse	523	operon_0216	5	fcluster_0220	78		hypothetical	Unknown	plasmid specific, in putida and Aeuriginosa		plasmid specific, in putida and Aeuriginosa	hypothetical protein CDS
**RPPX_RS28505**	475838	476164	forward	524	operon_0217	4	fcluster_0220	78		hypothetical	Unknown	plasmid specific, in putida and Aeuriginosa		plasmid specific, in putida and Aeuriginosa	hypothetical protein CDS
**RPPX_RS28510**	476397	476843	reverse	525	operon_0218	3	fcluster_0220	78		hypothetical	Unknown	plasmid specific, in putida and Aeuriginosa		plasmid specific, in putida and Aeuriginosa	hypothetical protein CDS
**RPPX_RS28515**	477349	478362	forward	526	operon_0219	5	fcluster_0220	78		hypothetical	Unknown	plasmid specific, in putida and Aeuriginosa		plasmid specific, in putida and Aeuriginosa	hypothetical protein CDS
**RPPX_RS28520**	478378	479430	forward	527	operon_0219	18	fcluster_0220	78		hypothetical	Unknown	plasmid specific, in putida and Aeuriginosa		plasmid specific, in putida and Aeuriginosa	hypothetical protein CDS
**RPPX_RS28525**	479438	480013	forward	528	operon_0219	2	fcluster_0220	78		hypothetical	Unknown	plasmid specific, in putida and Aeuriginosa		plasmid specific, in putida and Aeuriginosa	hypothetical protein CDS
**RPPX_RS28530**	480069	480506	reverse	529	operon_0220	5	fcluster_0220	78		hypothetical	Unknown	plasmid specific, in putida and Aeuriginosa		plasmid specific, in putida and Aeuriginosa	hypothetical protein CDS
**RPPX_RS28535**	480564	481112	reverse	530	operon_0220	5	fcluster_0220	78		hypothetical	Unknown	plasmid specific, in putida and Aeuriginosa		plasmid specific, in putida and Aeuriginosa	hypothetical protein CDS
**RPPX_RS28540**	481109	482047	reverse	531	operon_0220	5	fcluster_0220	78		hypothetical	Unknown	plasmid specific, in putida and Aeuriginosa		plasmid specific, in putida and Aeuriginosa	hypothetical protein CDS
**RPPX_RS28545**	482150	484198	reverse	532	operon_0220	4	fcluster_0220	78		hypothetical	Unknown	plasmid specific, in putida and Aeuriginosa		plasmid specific, in putida and Aeuriginosa	hypothetical protein CDS
**RPPX_RS28550**	484191	485615	reverse	533	operon_0220	11	fcluster_0220	78		hypothetical	Unknown	plasmid specific, in putida and Aeuriginosa		plasmid specific, in putida and Aeuriginosa	hypothetical protein CDS
**RPPX_RS28555**	485612	486505	reverse	534	operon_0220	18	fcluster_0220	78		hypothetical	Unknown	plasmid specific, in putida and Aeuriginosa		plasmid specific, in putida and Aeuriginosa	hypothetical protein CDS
**RPPX_RS28560**	486515	487318	reverse	535	operon_0220	11	fcluster_0220	78		hypothetical	Unknown	plasmid specific, in putida and Aeuriginosa		plasmid specific, in putida and Aeuriginosa	hypothetical protein CDS
**RPPX_RS28565**	487290	487994	reverse	536	operon_0220	9	fcluster_0220	78		hypothetical	Unknown	plasmid specific, in putida and Aeuriginosa		plasmid specific, in putida and Aeuriginosa	hypothetical protein CDS
**RPPX_RS28570**	487991	488338	reverse	537	operon_0220	1	fcluster_0220	78		hypothetical	Unknown	plasmid specific, in putida and Aeuriginosa		plasmid specific, in putida and Aeuriginosa	hypothetical protein CDS
**RPPX_RS28575**	488501	489256	reverse	538	operon_0221	373	fcluster_0220	78		hypothetical	Unknown	ISS12_Aachen			ATPase AAA CDS
**RPPX_RS28580**	489274	490788	reverse	539	operon_0221	175	fcluster_0220	78		hypothetical	Unknown	ISS12_Aachen			integrase CDS
**RPPX_RS28585**	490951	491217	forward	540	operon_0222	1	fcluster_0223	79		Conjugal transfer	Conjugation			truncated by ISS12	hypothetical protein CDS
**RPPX_RS28590**	491234	492511	reverse	541	operon_0223	5	fcluster_0223	79		Conjugal transfer	Conjugation			plasmid specific, in putida and Aeuriginosa	hypothetical protein CDS
**RPPX_RS28595**	492515	492892	reverse	542	operon_0223	17	fcluster_0223	79		Conjugal transfer	Conjugation			plasmid specific, in putida and Aeuriginosa	hypothetical protein CDS
**RPPX_RS28600**	492873	495821	reverse	543	operon_0223	16	fcluster_0223	79		Conjugal transfer	Conjugation			plasmid specific, in putida and Aeuriginosa	hypothetical protein CDS
**RPPX_RS28605**	495821	496294	reverse	544	operon_0223	11	fcluster_0223	79		Conjugal transfer	Conjugation			plasmid specific, in putida and Aeuriginosa	hypothetical protein CDS
**RPPX_RS28610**	496282	496605	reverse	545	operon_0223	16	fcluster_0223	79		Conjugal transfer	Conjugation		traV	plasmid specific, in putida and Aeuriginosa; This entry includes TraV, which is a component of conjugative type IV secretion system. TraV is an outer membrane lipoprotein that is believed to interact with the secretin TraK. The alignment contains three conserved cysteines in the N-terminal half.	hypothetical protein CDS
**RPPX_RS28615**	496615	498138	reverse	546	operon_0223	7	fcluster_0223	79		Conjugal transfer	Conjugation		traB	plasmid specific, in putida and Aeuriginosa	hypothetical protein CDS
**RPPX_RS28620**	498135	498929	reverse	547	operon_0223	17	fcluster_0223	79		Conjugal transfer	Conjugation			plasmid specific, in putida and Aeuriginosa	hypothetical protein CDS
**RPPX_RS28625**	498932	499558	reverse	548	operon_0223	12	fcluster_0223	79		Conjugal transfer	Conjugation			plasmid specific, in putida and Aeuriginosa	hypothetical protein CDS
**RPPX_RS28630**	499560	499937	reverse	549	operon_0223	6	fcluster_0223	79		Conjugal transfer	Conjugation			plasmid specific, in putida and Aeuriginosa	hypothetical protein CDS
**RPPX_RS28635**	499940	500335	reverse	550	operon_0223	6	fcluster_0223	79		Conjugal transfer	Conjugation			plasmid specific, in putida and Aeuriginosa	hypothetical protein CDS
**RPPX_RS28640**	500339	500689	reverse	551	operon_0223	3	fcluster_0223	79		Conjugal transfer	Conjugation			plasmid specific, in putida and Aeuriginosa	hypothetical protein CDS
**RPPX_RS28645**	500794	501135	reverse	552	operon_0223	6	fcluster_0223	79		Conjugal transfer	Conjugation			plasmid specific, in putida and Aeuriginosa	hypothetical protein CDS
**RPPX_RS28650**	501172	502983	reverse	553	operon_0223	20	fcluster_0223	79		Conjugal transfer	Conjugation	Conjugal transfer protein TraG	traG	plasmid specific, in putida and Aeuriginosa	hypothetical protein CDS
**RPPX_RS28655**	502958	505174	reverse	554	operon_0223	11	fcluster_0223	79		Conjugal transfer	Conjugation	Exodeoxyribonuclease		plasmid specific, in putida and Aeuriginosa	hypothetical protein CDS
**RPPX_RS28660**	505171	505656	reverse	555	operon_0223	6	fcluster_0223	79		Conjugal transfer	Conjugation			plasmid specific, in putida and Aeuriginosa	hypothetical protein CDS

locus_tag	lo.min	lo.max	Direction	X	df_s12.mp.Operon	ID	f.cluster	clus.x		f.cluster_function	f.cluster_fmain	Gene_function	gene_name	Comments	cds
**RPPX_RS28665**	506025	506816	forward	556	operon_0224	8	fcluster_0223	79		Conjugal transfer	Conjugation			not present in combination of previous cluster in other plasmids	hypothetical protein CDS
**RPPX_RS28670**	507056	508348	reverse	557	operon_0225	216	fcluster_0225	80		Conjugal transfer	Conjugation		trbI		conjugative transfer protein TrbI CDS
**RPPX_RS28675**	508351	509355	reverse	558	operon_0225	833	fcluster_0225	80		Conjugal transfer	Conjugation		trbG		conjugative transfer protein TrbG CDS
**RPPX_RS28680**	509352	510056	reverse	559	operon_0225	202	fcluster_0225	80		Conjugal transfer	Conjugation		trbF		conjugal transfer protein TrbF CDS
**RPPX_RS28685**	510074	511453	reverse	560	operon_0225	269	fcluster_0225	80		Conjugal transfer	Conjugation		trbL		conjugal transfer protein TrbL CDS
**RPPX_RS28690**	511450	511764	reverse	561	operon_0225	194	fcluster_0225	80		Conjugal transfer	Conjugation		trbK		hypothetical protein CDS
**RPPX_RS28695**	511777	512505	reverse	562	operon_0225	387	fcluster_0225	80		Conjugal transfer	Conjugation		trbJ		conjugal transfer protein TrbJ CDS
**RPPX_RS28700**	512502	514955	reverse	563	operon_0225	292	fcluster_0225	80		Conjugal transfer	Conjugation		trbE		conjugal transfer protein TrbE CDS
**RPPX_RS28705**	514968	515237	reverse	564	operon_0225	962	fcluster_0225	80		Conjugal transfer	Conjugation		trbD		conjugal transfer protein TrbD CDS
**RPPX_RS28710**	515234	515620	reverse	565	operon_0225	311	fcluster_0225	80		Conjugal transfer	Conjugation		trbC		conjugal transfer protein TrbC CDS
**RPPX_RS28715**	515617	516687	reverse	566	operon_0225	1067	fcluster_0225	80		Conjugal transfer	Conjugation		trbB		conjugal transfer protein TrbB CDS
**RPPX_RS28720**	516684	517172	reverse	567	operon_0225	371	fcluster_0225	80		Conjugal transfer	Conjugation		gene		CopG family transcriptional regulator CDS
**RPPX_RS28725**	517169	519175	reverse	568	operon_0225	1077	fcluster_0225	80		Conjugal transfer	Conjugation		traG		conjugal transfer protein TraG CDS
**RPPX_RS28730**	519411	519677	reverse	569	operon_0226	220	fcluster_0226	81		typeIV associated	Conjugation		hypothetical	trbK?	hypothetical protein CDS
**RPPX_RS28735**	519680	520624	reverse	570	operon_0226	571	fcluster_0226	81		typeIV associated	Conjugation		lysR		D-alanyl-D-alanine endopeptidase CDS
**RPPX_RS28740**	520639	522093	reverse	571	operon_0226	118	fcluster_0226	81		typeIV associated	Conjugation				MFS transporter CDS
**RPPX_RS28745**	522192	522992	forward	572	operon_0227	139	fcluster_0226	81		typeIV associated	Conjugation				XRE family transcriptional regulator CDS
**RPPX_RS28750**	523006	525012	reverse	573	operon_0228	394	fcluster_0226	81		typeIV associated	Conjugation	virD2	virD2		type VI secretion protein CDS
**RPPX_RS28755**	525461	526060	reverse	574	operon_0229	350	fcluster_0229	82		plasmid partitioning	Replication/partitioning				peptidase CDS
**RPPX_RS28760**	526057	526602	reverse	575	operon_0229	360	fcluster_0229	82		plasmid partitioning	Replication/partitioning		tnp		transposase CDS
**RPPX_RS28765**	526599	526886	reverse	576	operon_0229	411	fcluster_0229	82		plasmid partitioning	Replication/partitioning		parB		hypothetical protein CDS
**RPPX_RS28770**	526883	527521	reverse	577	operon_0229	362	fcluster_0229	82		plasmid partitioning	Replication/partitioning		parA		chromosome partitioning protein ParA CDS
**RPPX_RS28775**	527811	528671	reverse	578	operon_0230	15	fcluster_0230	83		plasmid replication intiation	Replication/partitioning	single-stranded DNA binding proteins tha	repA		RepA replication protein CDS
**RPPX_RS28780**	528686	528967	reverse	579	operon_0230	86	fcluster_0230	83		plasmid replication intiation	Replication/partitioning			transcriptional regulator ; CDD:315411	hypothetical protein CDS
**RPPX_RS28785**	529043	529849	reverse	580	operon_0230	296	fcluster_0230	83		plasmid replication intiation	Replication/partitioning	DUF2285		hypothetical protein;pfam10074<br>Uncharacterized conserved protein (DUF2285);<br>This domain, found in various hypothetical	hypothetical protein CDS
**RPPX_RS28790**	530171	530518	reverse	581	operon_0231	41	fcluster_0231	84		plasmid replication intiation	Replication/partitioning	lipoprotein		hypothetical protein	hypothetical protein CDS
**RPPX_RS28795**	530790	531086	forward	582	operon_0232	2	fcluster_0234	85		IGP synthesis	Degradation		xreR	XRE family transcriptional regulator	XRE family transcriptional regulator CDS
**RPPX_RS28800**	531318	532358	forward	583	operon_0233	64	fcluster_0234	85		IGP synthesis	Degradation	trpA, indole and glyceraldehyde-3-phosp	trpA	same operon, no space in between	tryptophan synthase subunit alpha CDS
**RPPX_RS28805**	532607	532921	reverse	584	operon_0234	205	fcluster_0234	85		IGP synthesis	Degradation			hypothetical	hypothetical protein CDS
**RPPX_RS28810**	533697	533906	reverse	585	operon_0235	141	fcluster_0235	86		plasmid replication	Replication/partitioning			hypothetical	hypothetical protein CDS
**RPPX_RS28815**	533968	536052	reverse	586	operon_0235	24	fcluster_0235	86		plasmid replication	Replication/partitioning	Plasmid stablization protein ParB	parB	locus_tag: CSC26_6499<br>note: Chromosome segregation protein Spo0J, contains ParB-like nuclease domain [Cell cycle control, cell division,<br>chromosome partitioning]; Region: Spo0J; COG1475<br>db_xref: CDD:224392	chromosome partitioning protein ParB CDS
**RPPX_RS28820**	536134	536964	reverse	587	operon_0235	914	fcluster_0235	86		plasmid replication	Replication/partitioning			note: Domain of unknown function (DUF932);<br>Region: DUF932; pfam06067<br>db_xref: CDD:310569	hypothetical protein CDS
**RPPX_RS28825**	537685	538812	forward	588	operon_0236	3	fcluster_0236	87		plasmid replication	Replication/partitioning				hypothetical protein CDS
**RPPX_RS28830**	538866	540629	forward	589	operon_0236	4	fcluster_0236	87		plasmid replication	Replication/partitioning				hypothetical protein CDS
**RPPX_RS28835**	540859	542649	forward	590	operon_0237	14	fcluster_0236	87		plasmid replication	Replication/partitioning	Chromosome partitioning protein			chromosome partitioning protein CDS
**RPPX_RS28840**	542646	543908	forward	591	operon_0237	2	fcluster_0236	87		plasmid replication	Replication/partitioning	DNA helicase (EC 3.6.4.12)			hypothetical protein CDS
**RPPX_RS28845**	544036	544545	reverse	592	operon_0238	193	fcluster_0238	88		DNA repair	Replication/partitioning		radC	Dna binding Hu and Rad c	DNA repair protein RadC CDS
**RPPX_RS28850**	544880	546046	reverse	593	operon_0239	56	fcluster_0238	88		DNA repair	Replication/partitioning	truncated by integrase, MOST IDENTITY \	hupB	Dna binding Hu and Rad c	hypothetical protein CDS
**RPPX_RS28855**	546043	547242	reverse	594	operon_0239	241	fcluster_0238	88		DNA repair	Replication/partitioning			Dna binding Hu and Rad c	integrase CDS
**RPPX_RS28860**	547478	547735	reverse	595	operon_0240	NA	fcluster_0238	88		DNA repair	Replication/partitioning	truncated by integrase, MOST IDENTITY \	hupB	Dna binding Hu and Rad c	DNA-binding protein HU CDS
**RPPX_RS28865**	547886	548206	reverse	596	operon_0241	6	fcluster_0247	89		hypothetical	Unknown			plasmids specific cluster	hypothetical protein CDS
**RPPX_RS28870**	548256	548603	reverse	597	operon_0241	6	fcluster_0247	89		hypothetical	Unknown			plasmids specific cluster	hypothetical protein CDS
**RPPX_RS28875**	548756	549178	forward	598	operon_0242	6	fcluster_0247	89		hypothetical	Unknown			plasmids specific cluster	RNA polymerase-binding protein DksA CDS
**RPPX_RS28880**	549297	549875	forward	599	operon_0242	6	fcluster_0247	89		hypothetical	Unknown			plasmids specific cluster	hypothetical protein CDS
**RPPX_RS28885**	549893	550969	forward	600	operon_0242	8	fcluster_0247	89		hypothetical	Unknown			plasmids specific cluster	hypothetical protein CDS
**RPPX_RS28890**	551102	551761	reverse	601	operon_0243	6	fcluster_0247	89		hypothetical	Unknown			plasmids specific cluster	hypothetical protein CDS
**RPPX_RS28895**	551909	553099	forward	602	operon_0244	6	fcluster_0247	89		hypothetical	Unknown			plasmids specific cluster	hypothetical protein CDS
**RPPX_RS28900**	553130	553966	forward	603	operon_0244	NA	fcluster_0247	89		hypothetical	Unknown			plasmids specific cluster	hypothetical protein CDS
**RPPX_RS28905**	554171	555685	forward	604	operon_0245	175	fcluster_0247	89		hypothetical	Unknown	ISS12_Aachen		ISS12_Aachen	integrase CDS
**RPPX_RS28910**	555703	556458	forward	605	operon_0245	373	fcluster_0247	89		hypothetical	Unknown	ISS12_Aachen		ISS12_Aachen	ATPase AAA CDS
**RPPX_RS28915**	556547	556807	forward	606	operon_0245	2	fcluster_0247	89		hypothetical	Unknown	N-acetyltransferase ESCO, zinc-finger		plasmids specific cluster	hypothetical protein CDS
**RPPX_RS28920**	556866	557399	reverse	607	operon_0246	8	fcluster_0247	89		hypothetical	Unknown			plasmids specific cluster	hypothetical protein CDS
**RPPX_RS28925**	557946	558842	forward	608	operon_0247	5	fcluster_0247	89		hypothetical	Unknown			plasmids specific cluster	hypothetical protein CDS
**RPPX_RS28930**	559663	560508	forward	609	operon_0248	67	fcluster_0257	90		heavy metal resistance	Heavy metal resistance		tnsA	Tn7	TnsA endonuclease-like protein CDS
**RPPX_RS28935**	560505	562670	forward	610	operon_0248	60	fcluster_0257	90		heavy metal resistance	Heavy metal resistance		tnsB	Tn7	transposase CDS
**RPPX_RS28940**	562667	564130	forward	611	operon_0248	55	fcluster_0257	90		heavy metal resistance	Heavy metal resistance		tnsC	Tn7	transposase CDS
**RPPX_RS28945**	564146	565822	forward	612	operon_0248	23	fcluster_0257	90		heavy metal resistance	Heavy metal resistance		tnsD	Tn7	hypothetical protein CDS
**RPPX_RS28950**	566132	566863	reverse	613	operon_0249	41	fcluster_0257	90		heavy metal resistance	Heavy metal resistance			Tn7	hypothetical protein CDS
**RPPX_RS28955**	566860	567300	reverse	614	operon_0249	121	fcluster_0257	90		heavy metal resistance	Heavy metal resistance			Tn7	membrane protein CDS
**RPPX_RS28960**	567305	567622	reverse	615	operon_0249	297	fcluster_0257	90		heavy metal resistance	Heavy metal resistance			Tn7	metal resistance protein CDS
**RPPX_RS28965**	568135	568872	forward	616	operon_0250	611	fcluster_0257	90		heavy metal resistance	Heavy metal resistance			Tn7	membrane protein CDS
**RPPX_RS28970**	568883	569560	forward	617	operon_0250	68	fcluster_0257	90		heavy metal resistance	Heavy metal resistance			Tn7	hypothetical protein CDS
**RPPX_RS28975**	569853	571295	forward	618	operon_0251	250	fcluster_0257	90		heavy metal resistance	Heavy metal resistance			Tn7	two-component system sensor histidine kinase CDS
**RPPX_RS28980**	571298	572269	reverse	619	operon_0252	188	fcluster_0257	90		heavy metal resistance	Heavy metal resistance			Tn7	hypothetical protein CDS
**RPPX_RS28985**	572313	573617	reverse	620	operon_0252	81	fcluster_0257	90		heavy metal resistance	Heavy metal resistance			Tn7	MFS transporter CDS
**RPPX_RS28990**	573865	574749	forward	621	operon_0253	55	fcluster_0257	90		heavy metal resistance	Heavy metal resistance		chrB	Tn7	chromate resistance protein CDS
**RPPX_RS28995**	574742	576112	forward	622	operon_0253	934	fcluster_0257	90		heavy metal resistance	Heavy metal resistance		chrA	Tn7	chromate transporter CDS
**RPPX_RS29000**	576181	576633	forward	623	operon_0253	132	fcluster_0257	90		heavy metal resistance	Heavy metal resistance		chrF	Tn7	chromate resistance protein; regulator CDS
**RPPX_RS29005**	576818	577117	reverse	624	operon_0254	1	fcluster_0257	90		heavy metal resistance	Heavy metal resistance			Tn7	hypothetical protein CDS
**RPPX_RS29010**	577722	578537	forward	625	operon_0255	5	fcluster_0257	90		heavy metal resistance	Heavy metal resistance			Tn7	hypothetical protein CDS
**RPPX_RS29015**	579630	580535	forward	626	operon_0256	9	fcluster_0257	90		heavy metal resistance	Heavy metal resistance			Tn7	transposase CDS
**RPPX_RS29020**	580625	581026	forward	627	operon_0256	25	fcluster_0257	90		heavy metal resistance	Heavy metal resistance			Tn7	hypothetical protein CDS
**RPPX_RS29025**	581057	581485	reverse	628	operon_0257	14	fcluster_0257	90		heavy metal resistance	Heavy metal resistance			Tn7	hypothetical protein CDS
**RPPX_RS29030**	581482	583335	reverse	629	operon_0257	45	fcluster_0257	90		heavy metal resistance	Heavy metal resistance			Tn7	hypothetical protein CDS
