# Supplementary material for: Comparative analysis reveals the modular functional structure of conjugative megaplasmid pTTS12 of Pseudomonas putida S12: A paradigm for transferable traits, plasmid stability, and inheritance?
Source: Front Microbiol. 2022 Sep 23;13:1001472. doi: 10.3389/fmicb.2022.1001472 (PMC9537497; doi:10.3389/fmicb.2022.1001472)
Supplement: Supplementary file 2 [file Data_Sheet_2.PDF]

Table S2. Top 500 plasmids with highest similarity score with pTTS12

Organism	Strain	Plasmid	taxid	slen	score	order	rank	fileName	Accession ID	Accession Version	score_c	relative_score	relative_precentage_similarity
Pseudomonas putida	S12	pTTS12	1215087	583900	15238212	6974	1	NZ_CP009975.1.gb	NZ_CP009975	NZ_CP009975.1	15238212	1.00	100.00%
Pseudomonas aeruginosa	PA96	pOZ176	1457392	500839	11053769	4156	2	NC_022344.1.gb	NC_022344	NC_022344.1	11053769	0.73	72.54%
Pseudomonas aeruginosa	FFUP_PS_37	pJB37	287	464804	10560643	61	3	KY494864.gb	KY494864	KY494864	10560643	0.69	69.30%
Pseudomonas aeruginosa	AR_0356	unnamed2	287	438531	10356784	13598	4	NZ_CP027170.1.gb	NZ_CP027170	NZ_CP027170.1	10356784	0.68	67.97%
Pseudomonas aeruginosa	AR441	unnamed3	287	438529	10091923	14271	5	NZ_CP029094.1.gb	NZ_CP029094	NZ_CP029094.1	10091923	0.66	66.23%
Pseudomonas putida	SY153	pSY153-MDR	303	468170	9859823	67	6	KY883660.gb	KY883660	KY883660	9859823	0.65	64.70%
Pseudomonas aeruginosa	T2436	pBT2436	287	422811	9857101	18227	7	NZ_CP039989.1.gb	NZ_CP039989	NZ_CP039989.1	9857101	0.65	64.69%
Pseudomonas koreensis	NA	p1	198620	467568	9819949	13729	8	NZ_CP027478.gb	NZ_CP027478	NZ_CP027478	9819949	0.64	64.44%
Pseudomonas putida	12969	p12969-DIM	303	409102	9818601	40	9	KU130294.gb	KU130294	KU130294	9818601	0.64	64.43%
Pseudomonas aeruginosa	T2101	pBT2101	287	439744	9817262	18228	10	NZ_CP039991.1.gb	NZ_CP039991	NZ_CP039991.1	9817262	0.64	64.43%
Pseudomonas aeruginosa	PA298	pBM908	287	395774	9750563	18264	11	NZ_CP040126.1.gb	NZ_CP040126	NZ_CP040126.1	9750563	0.64	63.99%
Pseudomonas aeruginosa		RW109	287	555265	9713654	21573	12	NZ_LT969519.1.gb	NZ_LT969519	NZ_LT969519.1	9713654	0.64	63.75%
Pseudomonas aeruginosa	PA121617	pBM413	287	423017	9630825	9346	13	NZ_CP016215.1.gb	NZ_CP016215	NZ_CP016215.1	9630825	0.63	63.20%
Pseudomonas aeruginosa	PABL048	pPABL048	287	414954	9572940	17989	14	NZ_CP039294.1.gb	NZ_CP039294	NZ_CP039294.1	9572940	0.63	62.82%
Pseudomonas aeruginosa		p727-IMP	287	430173	9562423	86	15	MF344568.gb	MF344568	MF344568	9562423	0.63	62.75%
Pseudomonas aeruginosa	AR439	unnamed2	287	437392	9447330	14273	16	NZ_CP029096.1.gb	NZ_CP029096	NZ_CP029096.1	9447330	0.62	62.00%
Pseudomonas citronellolis	SJTE-3	pRBL16	53408	370338	9313007	9278	17	NZ_CP015879.1.gb	NZ_CP015879	NZ_CP015879.1	9313007	0.61	61.12%
Pseudomonas aeruginosa		p12939-PER	287	496436	9274316	87	18	MF344569.gb	MF344569	MF344569	9274316	0.61	60.86%
Pseudomonas aeruginosa		pA681-IMP	287	397519	9067195	88	19	MF344570.gb	MF344570	MF344570	9067195	0.60	59.50%
Pseudomonas aeruginosa		pR31014-IMP	287	374000	8468991	89	20	MF344571.gb	MF344571	MF344571	8468991	0.56	55.58%
Pseudomonas taiwanensis	VLB120	pSTY	69328	321653	3299543	4211	21	NC_022739.1.gb	NC_022739	NC_022739.1	3299543	0.22	21.65%
Pseudomonas fluorescens	SBW25	pQBR103	216595	425094	3120653	1101	22	NC_009444.1.gb	NC_009444	NC_009444.1	3120653	0.20	20.48%
Pseudomonas syringae pv. maculicola	ES4326	pPma4326F	629265	387260	3072735	20349	23	NZ_CP047261.1.gb	NZ_CP047261	NZ_CP047261.1	3072735	0.20	20.16%
Pseudomonas putida	KF715	pKF715A	303	483376	2533747	5405	24	NZ_AP015030.1.gb	NZ_AP015030	NZ_AP015030.1	2533747	0.17	16.63%
Pseudomonas stutzeri	YC-YH1	pYCY1	316	225945	2447790	20654	25	NZ_JUDR01000010.gb	NZ_JUDR01000	NZ_JUDR01000010	2447790	0.16	16.06%
Pseudomonas fluorescens	SBW25	pQBR57	216595	307330	2201773	76	26	LN713926.gb	LN713926	LN713926	2201773	0.14	14.45%
Pseudomonas aeruginosa	PA83	unnamed1	287	398087	2197444	9755	27	NZ_CP017294.1.gb	NZ_CP017294	NZ_CP017294.1	2197444	0.14	14.42%
Pseudomonas aeruginosa	DN1	unnamed1	287	317349	2169823	10008	28	NZ_CP018048.1.gb	NZ_CP018048	NZ_CP018048.1	2169823	0.14	14.24%
Pseudomonas monteilii	FDAARGOS_171	???	76759	5836684	2110797	8472	29	NZ_CP014062.gb	NZ_CP014062	NZ_CP014062	2110797	0.14	13.85%
Salmonella enterica	8025	p8025	28901	311280	1826312	27	30	KP899803.gb	KP899803	KP899803	1826312	0.12	11.99%
Pseudomonas luteola	FDAARGOS_637	unnamed1	47886	585976	1664324	19371	31	NZ_CP044084.1.gb	NZ_CP044084	NZ_CP044084.1	1664324	0.11	10.92%
Enterobacter hormaechei subsp. steigerwaltii	34998	p34998-239.973kb	299766	239973	1583526	7751	32	NZ_CP012168.1.gb	NZ_CP012168	NZ_CP012168.1	1583526	0.10	10.39%
Enterobacter hormaechei	A1	pIncHI2-1502264	158836	309444	1580054	15037	33	NZ_CP031575.1.gb	NZ_CP031575	NZ_CP031575.1	1580054	0.10	10.37%
Leclercia adecarboxylata		pLec-476	83655	311758	1533549	60	34	KY320277.gb	KY320277	KY320277	1533549	0.10	10.06%
Enterobacter hormaechei subsp. hoffmannii	AR_0365	unnamed1	1812934	328871	1521394	13577	35	NZ_CP027144.1.gb	NZ_CP027144	NZ_CP027144.1	1521394	0.10	9.98%
Azospira sp. I09	I09	pAZI09	1765049	397391	1520888	5819	36	NZ_AP021845.1.gb	NZ_AP021845	NZ_AP021845.1	1520888	0.10	9.98%
Citrobacter freundii	SL151	unnamed1	546	229406	1507508	9655	37	NZ_CP017058.1.gb	NZ_CP017058	NZ_CP017058.1	1507508	0.10	9.89%
Cupriavidus metallidurans	FDAARGOS_675	unnamed3	119219	2586495	1454205	20196	38	NZ_CP046333.1.gb	NZ_CP046333	NZ_CP046333.1	1454205	0.10	9.54%
Cupriavidus metallidurans CH34	CH34	megaplasmid	266264	2580084	1452805	930	39	NC_007974.2.gb	NC_007974	NC_007974.2	1452805	0.10	9.53%
Enterobacter hormaechei subsp. hormaechei	34983	p34983-328.905kb	301105	328905	1434530	7150	40	NZ_CP010380.1.gb	NZ_CP010380	NZ_CP010380.1	1434530	0.09	9.41%
Escherichia coli	CFSAN064035	pGMI17-003_1	562	310064	1400984	14889	41	NZ_CP031135.1.gb	NZ_CP031135	NZ_CP031135.1	1400984	0.09	9.19%
Pseudomonas sp. XWY-1	XWY-1	pXWY	2069256	394537	1394999	13268	42	NZ_CP026333.1.gb	NZ_CP026333	NZ_CP026333.1	1394999	0.09	9.15%
Klebsiella oxytoca	CAV1374	pKPC_CAV1374	571	332956	1389107	7629	43	NZ_CP011635.1.gb	NZ_CP011635	NZ_CP011635.1	1389107	0.09	9.12%
Pseudomonas veronii 1YdBTEX2	1YdBTEX2	PVE_plasmid	1295141	373858	1381095	79	44	LT599585.gb	LT599585	LT599585	1381095	0.09	9.06%
Pantoea sp. PSNIH2	NA	pPSP-75c	1484157	378808	1377395	6935	45	NZ_CP009869.gb	NZ_CP009869	NZ_CP009869	1377395	0.09	9.04%
Klebsiella michiganensis	AR375	unnamed2	1134687	340462	1364714	14308	46	NZ_CP029142.1.gb	NZ_CP029142	NZ_CP029142.1	1364714	0.09	8.96%
Citrobacter freundii complex sp. CFNIH9	CFNIH9	pCFR-eb27	2077149	355789	1344375	13236	47	NZ_CP026242.1.gb	NZ_CP026242	NZ_CP026242.1	1344375	0.09	8.82%
Ralstonia solanacearum	HA4-1	HA4-1MP	305	1947245	1344168	11754	48	NZ_CP022482.1.gb	NZ_CP022482	NZ_CP022482.1	1344168	0.09	8.82%
Enterobacteriaceae bacterium ENNIH1	ENNIH1	pENT-1f0b	2066051	302640	1338238	13201	49	NZ_CP026196.1.gb	NZ_CP026196	NZ_CP026196.1	1338238	0.09	8.78%
Leclercia sp. LSNIH1	LSNIH1	pLEC-1cb1	1920114	341250	1332411	13180	50	NZ_CP026171.1.gb	NZ_CP026171	NZ_CP026171.1	1332411	0.09	8.74%
Leclercia sp. LSNIH3	LSNIH3	pLEC-7c0d	1920116	330021	1332088	13284	51	NZ_CP026391.1.gb	NZ_CP026391	NZ_CP026391.1	1332088	0.09	8.74%
Pantoea sp. PSNIH1	PSNIH1	pPSP-a3e	1484158	331227	1319928	6944	52	NZ_CP009883.1.gb	NZ_CP009883	NZ_CP009883.1	1319928	0.09	8.66%
Pseudomonas resinovorans		pCAR1	53412	199035	1317771	441	53	NC_004444.1.gb	NC_004444	NC_004444.1	1317771	0.09	8.65%
Pseudomonas putida	CA10	pCAR1.2	303	200231	1317771	1691	54	NC_011838.1.gb	NC_011838	NC_011838.1	1317771	0.09	8.65%
Klebsiella michiganensis	FDAARGOS_647	unnamed2	1134687	490750	1278379	19383	55	NZ_CP044111.1.gb	NZ_CP044111	NZ_CP044111.1	1278379	0.08	8.39%
Klebsiella sp. LTGPAF-6F	LTGPAF-6F	unnamed1	1905288	526446	1275312	9830	56	NZ_CP017451.1.gb	NZ_CP017451	NZ_CP017451.1	1275312	0.08	8.37%
Salmonella enterica subsp. enterica serovar Quebec str. S-1267	S-1267	???	1243591	4626699	1274416	11569	57	NZ_CP022019.gb	NZ_CP022019	NZ_CP022019	1274416	0.08	8.36%
Pseudomonas mandelii JR-1	JR-1	unnamed	1147786	410512	1261463	6329	58	NZ_CP005961.1.gb	NZ_CP005961	NZ_CP005961.1	1261463	0.08	8.28%
Enterobacter cloacae	N1863	pN1863-HI2	550	349834	1259548	93	59	MF344583.gb	MF344583	MF344583	1259548	0.08	8.27%
Pseudomonas veronii	Pvy	unnamed	76761	194607	1255599	18103	60	NZ_CP039632.2.gb	NZ_CP039632	NZ_CP039632.2	1255599	0.08	8.24%
Klebsiella quasipneumoniae subsp. quasipneumoniae	M17277	p17277A_477	1667327	477340	1248813	19322	61	NZ_CP043927.1.gb	NZ_CP043927	NZ_CP043927.1	1248813	0.08	8.20%
Pseudomonas aeruginosa VRFPA03	VRFPA03	MUM024	1350465	54061	1244212	5	62	ATNK01000020.gb	ATNK01000020	ATNK01000020	1244212	0.08	8.17%
Polaromonas naphthalenivorans CJ2	CJ2	pPNAP01	365044	353291	1243539	1044	63	NC_008757.1.gb	NC_008757	NC_008757.1	1243539	0.08	8.16%
Escherichia coli	ECONIH5	pECO-109b	562	298633	1218860	13210	64	NZ_CP026208.1.gb	NZ_CP026208	NZ_CP026208.1	1218860	0.08	8.00%
Cupriavidus necator N-1	N-1	pBB1	1042878	1499175	1217109	2597	65	NC_015727.1.gb	NC_015727	NC_015727.1	1217109	0.08	7.99%
Klebsiella pneumoniae	AR_0158	tig00000727	573	354705	1201969	11397	66	NZ_CP021699.1.gb	NZ_CP021699	NZ_CP021699.1	1201969	0.08	7.89%
Klebsiella pneumoniae	Kp2964	2964TF	573	170244	1200536	39	67	KT935446.gb	KT935446	KT935446	1200536	0.08	7.88%
Enterobacter roggenkampii	35734	p35734-141.404kb	1812935	141402	1198260	7135	68	NZ_CP010360.2.gb	NZ_CP010360	NZ_CP010360.2	1198260	0.08	7.86%
Pseudomonas putida DOT-T1E	DOT-T1E	pGRT1	1196325	133451	1195707	2609	69	NC_015855.1.gb	NC_015855	NC_015855.1	1195707	0.08	7.85%
Ralstonia solanacearum	RSCM	p-unname2	305	2212170	1191862	13114	70	NZ_CP025986.1.gb	NZ_CP025986	NZ_CP025986.1	1191862	0.08	7.82%

Organism	Strain	Plasmid	taxid	slen	score	order	rank	fileName	Accession ID	Accession Version	score_c	relative_score	relative_precentage_similarity
Citrobacter freundii	AR_0116	unnamed1	546	260460	1189856	15285	71	NZ_CP032179.1.gb	NZ_CP032179	NZ_CP032179.1	1189856	0.08	7.81%
Burkholderia contaminans	CH-1	pBC453	488447	453462	1184879	5610	72	NZ_AP018360.1.gb	NZ_AP018360	NZ_AP018360.1	1184879	0.08	7.78%
Enterobacter hormaechei subsp. steigerwaltii	34998	p34998-210.894kb	299766	210894	1182114	7752	73	NZ_CP012169.1.gb	NZ_CP012169	NZ_CP012169.1	1182114	0.08	7.76%
Citrobacter freundii	MRSN 12115	unnamed	546	291028	1181913	20655	74	NZ_JYFZ02000002.gb	NZ_JYFZ020000	NZ_JYFZ02000002	1181913	0.08	7.76%
Cupriavidus taiwanensis		II	164546	2795658	1165938	21594	75	NZ_LT984804.1.gb	NZ_LT984804	NZ_LT984804.1	1165938	0.08	7.65%
Pseudomonas resinovorans NBRC 106553	NBRC 106553	pCAR1.3	1245471	198965	1159891	4020	76	NC_021506.1.gb	NC_021506	NC_021506.1	1159891	0.08	7.61%
Ralstonia solanacearum	YC45	unnamed	305	2008422	1159179	15	77	CP011998.gb	CP011998	CP011998	1159179	0.08	7.61%
Novosphingobium resinovorum	SA1	pSA1	158500	1756808	1155219	9659	78	NZ_CP017076.1.gb	NZ_CP017076	NZ_CP017076.1	1155219	0.08	7.58%
Klebsiella pneumoniae subsp. pneumoniae KPNIH27	KPNIH27	pKEC-dc3	1328324	268334	1149416	6562	79	NZ_CP007732.1.gb	NZ_CP007732	NZ_CP007732.1	1149416	0.08	7.54%
Citrobacter freundii CFNIH1	CFNIH1	pKEC-a3c	1333848	272297	1146816	6517	80	NZ_CP007558.1.gb	NZ_CP007558	NZ_CP007558.1	1146816	0.08	7.53%
uncultured bacterium		pKAZ5	77133	222486	1144806	33	81	KR827394.gb	KR827394	KR827394	1144806	0.08	7.51%
Enterobacter cloacae ECNIH2	ECNIH2	pKEC-39c	1333850	319976	1138613	6608	82	NZ_CP008824.1.gb	NZ_CP008824	NZ_CP008824.1	1138613	0.07	7.47%
Sinorhizobium meliloti RU11	1	pSymA	1401243	1613292	1129985	11233	83	NZ_CP021217.1.gb	NZ_CP021217	NZ_CP021217.1	1129985	0.07	7.42%
Enterobacter cloacae	MRSN17626	pMRVIM0813	550	311662	1129267	29	84	KP975077.gb	KP975077	KP975077	1129267	0.07	7.41%
Sinorhizobium meliloti SM11	SM11	pSmeSM11c	707241	1633319	1128612	2932	85	NC_017327.1.gb	NC_017327	NC_017327.1	1128612	0.07	7.41%
Ralstonia solanacearum	SEPPX05	pSEPPX05	305	2066085	1128513	11298	86	NZ_CP021449.1.gb	NZ_CP021449	NZ_CP021449.1	1128513	0.07	7.41%
Cupriavidus taiwanensis		II	164546	2796980	1128111	21596	87	NZ_LT984807.1.gb	NZ_LT984807	NZ_LT984807.1	1128111	0.07	7.40%
Azospirillum sp. B510	B510	pAB510a	137722	1455109	1123750	2254	88	NC_013855.1.gb	NC_013855	NC_013855.1	1123750	0.07	7.37%
Hydrogenophaga sp. PBL-H3	PBL-H3	PBL-H3(B4)	434010	319548	1119567	19787	89	NZ_CP044973.1.gb	NZ_CP044973	NZ_CP044973.1	1119567	0.07	7.35%
Hydrogenophaga sp. PBL-H3	PBL-H3	PBL-H3(B2)	434010	319548	1118367	19789	90	NZ_CP044976.1.gb	NZ_CP044976	NZ_CP044976.1	1118367	0.07	7.34%
Cupriavidus oxalaticus	T2	unnamed1	96344	568815	1116165	15447	91	NZ_CP032520.1.gb	NZ_CP032520	NZ_CP032520.1	1116165	0.07	7.32%
Burkholderia sp. IDO3	IDO3	p1	1705310	420805	1113537	14227	92	NZ_CP028965.1.gb	NZ_CP028965	NZ_CP028965.1	1113537	0.07	7.31%
Ensifer adhaerens	Corn53	AB	106592	1583018	1112687	14716	93	NZ_CP030264.1.gb	NZ_CP030264	NZ_CP030264.1	1112687	0.07	7.30%
Escherichia coli	S38	pS38	562	247885	1108306	48	94	KX129782.gb	KX129782	KX129782	1108306	0.07	7.27%
Pseudomonas fluorescens	PC24	pPHE24	294	120754	1106849	62	95	KY503037.gb	KY503037	KY503037	1106849	0.07	7.26%
Cupriavidus taiwanensis		II	164546	2827766	1098620	21724	96	NZ_LT991977.1.gb	NZ_LT991977	NZ_LT991977.1	1098620	0.07	7.21%
Salmonella enterica subsp. enterica serovar Infantis		pRH-R27	595	299305	1092240	75	97	LN555650.gb	LN555650	LN555650	1092240	0.07	7.17%
Salmonella enterica subsp. enterica	15-SA01028	pSE15-SA01028	59201	310921	1085623	13384	98	NZ_CP026661.1.gb	NZ_CP026661	NZ_CP026661.1	1085623	0.07	7.12%
Sinorhizobium fredii HH103	HH103	pSfHH103e	1117943	2096125	1079323	2731	99	NC_016815.1.gb	NC_016815	NC_016815.1	1079323	0.07	7.08%
Klebsiella aerogenes	AR_0161	unnamed	548	451422	1076739	14222	100	NZ_CP028952.1.gb	NZ_CP028952	NZ_CP028952.1	1076739	0.07	7.07%
Rhizobium jaguaris	CCGE525	pRCCGE525c	1312183	2584926	1073949	15504	101	NZ_CP032695.1.gb	NZ_CP032695	NZ_CP032695.1	1073949	0.07	7.05%
Escherichia coli	ECONIH5	pECO-dc1b	562	202687	1072671	13209	102	NZ_CP026207.1.gb	NZ_CP026207	NZ_CP026207.1	1072671	0.07	7.04%
Cupriavidus taiwanensis		IV	164546	2595033	1071313	21593	103	NZ_LT984802.1.gb	NZ_LT984802	NZ_LT984802.1	1071313	0.07	7.03%
Klebsiella pneumoniae	NA	unnamed	573	457578	1070821	13787	104	NZ_CP027603.gb	NZ_CP027603	NZ_CP027603	1070821	0.07	7.03%
Enterobacter hormaechei	TUM11043	pMTY11043_IncHI2	158836	368940	1070543	4	105	AP018352.gb	AP018352	AP018352	1070543	0.07	7.03%
Enterobacter cloacae complex sp.		pC45-VIM4	2027919	299117	1070445	21721	106	NZ_LT991958.1.gb	NZ_LT991958	NZ_LT991958.1	1070445	0.07	7.02%
Escherichia coli	BK32533	pBK32533	562	241963	1065114	26	107	KP345882.gb	KP345882	KP345882	1065114	0.07	6.99%
Sinorhizobium fredii NGR234	NGR234	pNGR234b	394	2430033	1064989	1960	108	NC_012586.1.gb	NC_012586	NC_012586.1	1064989	0.07	6.99%
Paraburkholderia caribensis MBA4	MBA4	unnamed	1323664	2555069	1057417	7951	109	NZ_CP012748.1.gb	NZ_CP012748	NZ_CP012748.1	1057417	0.07	6.94%
Pseudomonas aeruginosa	HN39	pHN39-SIM	287	282042	1051313	41	110	KU254577.gb	KU254577	KU254577	1051313	0.07	6.90%
Bradyrhizobium guangxiense	CCBAU 53363	p53363	1325115	979173	1051087	11650	111	NZ_CP022220.1.gb	NZ_CP022220	NZ_CP022220.1	1051087	0.07	6.90%
Leclercia adecarboxylata	pP10164	pP10164-2	83655	315395	1050042	51	112	KX710093.gb	KX710093	KX710093	1050042	0.07	6.89%
Bradyrhizobium guangdongense	CCBAU 51649	unnamed	1325090	981946	1049008	14631	113	NZ_CP030052.1.gb	NZ_CP030052	NZ_CP030052.1	1049008	0.07	6.88%
Bradyrhizobium guangzhouense	CCBAU 51670	unnamed1	1325095	979291	1049008	14632	114	NZ_CP030054.1.gb	NZ_CP030054	NZ_CP030054.1	1049008	0.07	6.88%
Aeromonas hydrophila subsp. hydrophila	WCHAH045096	pMCR5_045096	196023	241090	1046533	14040	115	NZ_CP028567.2.gb	NZ_CP028567	NZ_CP028567.2	1046533	0.07	6.87%
Escherichia coli	SLK172	pSLK172-1	562	369298	1043900	9871	116	NZ_CP017632.1.gb	NZ_CP017632	NZ_CP017632.1	1043900	0.07	6.85%
Enterobacter hormaechei	C4	pC4_001	158836	155361	1043411	19060	117	NZ_CP042541.1.gb	NZ_CP042541	NZ_CP042541.1	1043411	0.07	6.85%
Enterobacter hormaechei	C44	pC44_001	158836	150626	1042938	19082	118	NZ_CP042567.1.gb	NZ_CP042567	NZ_CP042567.1	1042938	0.07	6.84%
Ensifer adhaerens	Corn53	AA	106592	1790007	1042539	14715	119	NZ_CP030263.1.gb	NZ_CP030263	NZ_CP030263.1	1042539	0.07	6.84%
Pseudomonas aeruginosa		pUM505	287	123322	1041209	2680	120	NC_016138.1.gb	NC_016138	NC_016138.1	1041209	0.07	6.83%
Ralstonia solanacearum	EP1	plasmid	305	2093441	1040587	8959	121	NZ_CP015116.1.gb	NZ_CP015116	NZ_CP015116.1	1040587	0.07	6.83%
Sinorhizobium meliloti	USDA1021	psymB	382	2011522	1040572	11458	122	NZ_CP021802.1.gb	NZ_CP021802	NZ_CP021802.1	1040572	0.07	6.83%
Niveispirillum cyanobacteriorum	TH16	unnamed1	1612173	1003851	1036369	12992	123	NZ_CP025613.1.gb	NZ_CP025613	NZ_CP025613.1	1036369	0.07	6.80%
Rhizobium sp. 11515TR	10195	p11515TR-A	2028343	1569179	1034893	11896	124	NZ_CP022999.1.gb	NZ_CP022999	NZ_CP022999.1	1034893	0.07	6.79%
Ralstonia solanacearum GMI1000	GMI1000	unnamed	267608	2094509	1031259	385	125	NC_003296.1.gb	NC_003296	NC_003296.1	1031259	0.07	6.77%
Ralstonia pseudosolanacearum	RS 476	unnamed	1310165	2094505	1031259	11441	126	NZ_CP021763.1.gb	NZ_CP021763	NZ_CP021763.1	1031259	0.07	6.77%
Ralstonia solanacearum FJAT-91	FJAT-91	unnamed1	1130829	2000873	1027923	9495	127	NZ_CP016613.1.gb	NZ_CP016613	NZ_CP016613.1	1027923	0.07	6.75%
Citrobacter freundii	680	p680_1	546	385971	1025587	17719	128	NZ_CP038659.1.gb	NZ_CP038659	NZ_CP038659.1	1025587	0.07	6.73%
Enterobacter hormaechei	WCHEH020038	pCTXM9_020038	158836	296580	1023374	15100	129	NZ_CP031724.1.gb	NZ_CP031724	NZ_CP031724.1	1023374	0.07	6.72%
Ralstonia pseudosolanacearum	CRMRs218	unnamed	1310165	2094523	1021548	11442	130	NZ_CP021765.1.gb	NZ_CP021765	NZ_CP021765.1	1021548	0.07	6.70%
Citrobacter freundii	C50	pC50_001	546	243455	1019031	19026	131	NZ_CP042479.1.gb	NZ_CP042479	NZ_CP042479.1	1019031	0.07	6.69%
Citrobacter freundii	E33	pE33_001	546	243455	1019031	19052	132	NZ_CP042518.1.gb	NZ_CP042518	NZ_CP042518.1	1019031	0.07	6.69%
Enterobacter cloacae	T5282	pT5282-mphA	550	282423	1018644	57	133	KY270852.gb	KY270852	KY270852	1018644	0.07	6.68%
Klebsiella pneumoniae	KLPN57	I	573	321455	1018150	21438	134	NZ_LT882698.1.gb	NZ_LT882698	NZ_LT882698.1	1018150	0.07	6.68%
Cronobacter sakazakii	GZcsf-1	pGW1	28141	340723	1017008	14235	135	NZ_CP028975.1.gb	NZ_CP028975	NZ_CP028975.1	1017008	0.07	6.67%
Massilia putida	6NM-7	unnamed2	1141883	457181	1015094	10465	136	NZ_CP019037.1.gb	NZ_CP019037	NZ_CP019037.1	1015094	0.07	6.66%
Sinorhizobium meliloti BL225C	BL225C	pSINMEB01	698936	1616209	1013098	2930	137	NC_017324.1.gb	NC_017324	NC_017324.1	1013098	0.07	6.65%
Sphingobium yanoikuyae	SJTF8	pF1	13690	505328	1012568	15689	138	NZ_CP033227.1.gb	NZ_CP033227	NZ_CP033227.1	1012568	0.07	6.64%
Citrobacter sp. CRE-46	AR_0157	unnamed2	1703250	136246	1011706	14497	139	NZ_CP029730.1.gb	NZ_CP029730	NZ_CP029730.1	1011706	0.07	6.64%
Klebsiella oxytoca	KONIH2	pKOR-01e8	571	198115	1010065	13250	140	NZ_CP026281.1.gb	NZ_CP026281	NZ_CP026281.1	1010065	0.07	6.63%
Pseudomonas monteilii	B5	pSH5-1	76759	130536	1008689	11781	141	NZ_CP022563.1.gb	NZ_CP022563	NZ_CP022563.1	1008689	0.07	6.62%
Rhizobium favelukesii	LPU83	pLPU83d	348824	1932030	1006726	20640	142	NZ_HG916855.1.gb	NZ_HG916855	NZ_HG916855.1	1006726	0.07	6.61%
Sinorhizobium meliloti	KH46	psymA	382	1597380	1004673	11475	143	NZ_CP021824.1.gb	NZ_CP021824	NZ_CP021824.1	1004673	0.07	6.59%
Sinorhizobium meliloti	Rm41	psymA	382	1549593	1003590	11463	144	NZ_CP021809.1.gb	NZ_CP021809	NZ_CP021809.1	1003590	0.07	6.59%

Organism	Strain	Plasmid	taxid	slen	score	order	rank	fileName	Accession ID	Accession Version	score_c	relative_score	relative_precentage_similarity
Escherichia coli R178		pRH-R178	1408252	223382	1003003	19	145	HG530658.gb	HG530658	HG530658	1003003	0.07	6.58%
Sinorhizobium meliloti Rm41	Rm41	pSYMA	1230587	1559666	1002892	3225	146	NC_018683.1.gb	NC_018683	NC_018683.1	1002892	0.07	6.58%
Escherichia coli	ECONIH4	pECO-c85f	562	186884	998799	13296	147	NZ_CP026405.1.gb	NZ_CP026405	NZ_CP026405.1	998799	0.07	6.55%
Aeromonas salmonicida	S44	pS44-1	645	216870	995388	11632	148	NZ_CP022176.1.gb	NZ_CP022176	NZ_CP022176.1	995388	0.07	6.53%
Escherichia coli	CFS3292	pCFS3292-1	562	192448	995142	13496	149	NZ_CP026936.1.gb	NZ_CP026936	NZ_CP026936.1	995142	0.07	6.53%
Klebsiella pneumoniae	A324	pA324-IMP	573	271153	994360	84	150	MF344566.gb	MF344566	MF344566	994360	0.07	6.53%
Enterobacter cloacae subsp. cloacae ATCC 13047	ATCC 13047	pECL_A	716541	199562	994124	2306	151	NC_014107.1.gb	NC_014107	NC_014107.1	994124	0.07	6.52%
Ralstonia solanacearum	T523	p10319MP	305	2069295	993437	11819	152	NZ_CP022703.1.gb	NZ_CP022703	NZ_CP022703.1	993437	0.07	6.52%
Sinorhizobium meliloti 1021	1021	pSymA	266834	1354226	992597	355	153	NC_003037.1.gb	NC_003037	NC_003037.1	992597	0.07	6.51%
Rhizobium tropici CIAT 899	CIAT 899	pRtrCIAT899c	698761	2083197	991874	3786	154	NC_020062.1.gb	NC_020062	NC_020062.1	991874	0.07	6.51%
Burkholderia vietnamiensis G4	G4	pBVIE02	269482	265616	991727	12	155	CP000618.gb	CP000618	CP000618	991727	0.07	6.51%
Escherichia coli	A1_181	p_unnamed1_KPC2	562	210031	990485	18244	156	NZ_CP040068.1.gb	NZ_CP040068	NZ_CP040068.1	990485	0.07	6.50%
Paraburkholderia aromaticivorans	BN5	pBN2	2026199	489416	989545	11891	157	NZ_CP022992.1.gb	NZ_CP022992	NZ_CP022992.1	989545	0.06	6.49%
Ralstonia solanacearum FQY_4	FQY_4	megaplasmid	1262456	2089828	989454	4074	158	NC_021745.1.gb	NC_021745	NC_021745.1	989454	0.06	6.49%
Klebsiella pneumoniae JM45	JM45	p1	1380908	317154	988765	4137	159	NC_022078.1.gb	NC_022078	NC_022078.1	988765	0.06	6.49%
Escherichia coli	CFS3273	pCFS3273-1	562	268664	988556	13494	160	NZ_CP026933.1.gb	NZ_CP026933	NZ_CP026933.1	988556	0.06	6.49%
Rhizobium grahamii	BG7	unnamed	1120045	1961522	987988	19253	161	NZ_CP043499.1.gb	NZ_CP043499	NZ_CP043499.1	987988	0.06	6.48%
Sinorhizobium fredii CCBAU 83666	CCBAU 83666	pSF83666b	1128334	2277056	986803	11932	162	NZ_CP023071.1.gb	NZ_CP023071	NZ_CP023071.1	986803	0.06	6.48%
Ralstonia solanacearum	SL3300	unnamed	305	2033766	983280	11857	163	NZ_CP022787.1.gb	NZ_CP022787	NZ_CP022787.1	983280	0.06	6.45%
Citrobacter freundii	525011	p525011-HI2	546	354045	982923	92	164	MF344582.gb	MF344582	MF344582	982923	0.06	6.45%
Mycolicibacterium arabiense	JCM 18538	pJCM18538	1286181	6017160	981882	5844	165	NZ_AP022593.1.gb	NZ_AP022593	NZ_AP022593.1	981882	0.06	6.44%
Escherichia coli	ATCC BAA-196	unnamed1	562	279992	978569	19159	166	NZ_CP042868.1.gb	NZ_CP042868	NZ_CP042868.1	978569	0.06	6.42%
Ralstonia solanacearum	KACC 10709	unnamed1	305	2145732	977393	9620	167	NZ_CP016905.1.gb	NZ_CP016905	NZ_CP016905.1	977393	0.06	6.41%
Ralstonia solanacearum	SL3103	unnamed	305	2134747	977259	11859	168	NZ_CP022791.1.gb	NZ_CP022791	NZ_CP022791.1	977259	0.06	6.41%
Burkholderia sp. YI23	NA	byi_2p	1097668	356263	977071	2692	169	NC_016591.gb	NC_016591	NC_016591	977071	0.06	6.41%
Agrobacterium tumefaciens	CFBP7129	pAtCFBP7129a	358	473557	976031	18199	170	NZ_CP039924.1.gb	NZ_CP039924	NZ_CP039924.1	976031	0.06	6.41%
Sphingobium sp. RAC03	RAC03	pBSY17_1	1843368	892708	974643	9411	171	NZ_CP016453.1.gb	NZ_CP016453	NZ_CP016453.1	974643	0.06	6.40%
Enterobacter hormaechei	EB_P9_L5_03.19	pIMPIncH12_331kb	158836	331049	974352	19298	172	NZ_CP043767.1.gb	NZ_CP043767	NZ_CP043767.1	974352	0.06	6.39%
Pseudomonas fluorescens	P69	pG69	294	144433	972363	109	173	MH061177.gb	MH061177	MH061177	972363	0.06	6.38%
Citrobacter freundii	154	p154_1	546	296117	971649	17716	174	NZ_CP038654.1.gb	NZ_CP038654	NZ_CP038654.1	971649	0.06	6.38%
Klebsiella quasipneumoniae	CAV2013	pKPC_CAV2013	1463165	447095	971334	14405	175	NZ_CP029436.1.gb	NZ_CP029436	NZ_CP029436.1	971334	0.06	6.37%
Klebsiella quasipneumoniae	CAV2018	pKPC_CAV2018-435	1463165	435125	970520	14401	176	NZ_CP029431.1.gb	NZ_CP029431	NZ_CP029431.1	970520	0.06	6.37%
Burkholderia sp. YI23	NA	byi_1p	1097668	1951047	970451	2709	177	NC_016626.gb	NC_016626	NC_016626	970451	0.06	6.37%
Ralstonia solanacearum	T60	unnamed	305	2071487	969316	11848	178	NZ_CP022769.1.gb	NZ_CP022769	NZ_CP022769.1	969316	0.06	6.36%
Ralstonia solanacearum	SL3882	unnamed	305	2082957	969316	11853	179	NZ_CP022779.1.gb	NZ_CP022779	NZ_CP022779.1	969316	0.06	6.36%
Ralstonia solanacearum	SL3822	unnamed	305	2134808	967467	11854	180	NZ_CP022781.1.gb	NZ_CP022781	NZ_CP022781.1	967467	0.06	6.35%
Ralstonia solanacearum	T78	unnamed	305	2063152	966191	11846	181	NZ_CP022766.1.gb	NZ_CP022766	NZ_CP022766.1	966191	0.06	6.34%
Ralstonia solanacearum	T117	unnamed	305	2061886	966028	11841	182	NZ_CP022756.1.gb	NZ_CP022756	NZ_CP022756.1	966028	0.06	6.34%
Enterobacter cloacae	174	unnamed1	550	400064	963599	10961	183	NZ_CP020529.1.gb	NZ_CP020529	NZ_CP020529.1	963599	0.06	6.32%
Ensifer sojae CCBAU 05684	CCBAU 05684	pSJ05684b	716928	2011513	963334	11930	184	NZ_CP023068.1.gb	NZ_CP023068	NZ_CP023068.1	963334	0.06	6.32%
Raoultella ornithinolytica	Ro24724	pRo24724	54291	446611	962645	11251	185	NZ_CP021328.1.gb	NZ_CP021328	NZ_CP021328.1	962645	0.06	6.32%
Enterobacter hormaechei	C15117	pSPRC-Echo1	158836	339920	962634	15552	186	NZ_CP032842.1.gb	NZ_CP032842	NZ_CP032842.1	962634	0.06	6.32%
Sinorhizobium meliloti	RMO17	pSymA	382	1466845	962609	6724	187	NZ_CP009145.1.gb	NZ_CP009145	NZ_CP009145.1	962609	0.06	6.32%
Escherichia coli	ATCC BAA-196	unnamed1	562	266396	961903	19158	188	NZ_CP042866.1.gb	NZ_CP042866	NZ_CP042866.1	961903	0.06	6.31%
Citrobacter freundii complex sp. CFNIH4	CFNIH4	pCFR-0b27	2077148	206795	961571	13229	189	NZ_CP026233.1.gb	NZ_CP026233	NZ_CP026233.1	961571	0.06	6.31%
Klebsiella pneumoniae	K66-45	pK66-45-1	573	338512	961064	11080	190	NZ_CP020902.1.gb	NZ_CP020902	NZ_CP020902.1	961064	0.06	6.31%
Raoultella planticola	KpNDM1	pKpNDM1	575	277682	960489	4327	191	NC_023911.1.gb	NC_023911	NC_023911.1	960489	0.06	6.30%
Pseudomonas stutzeri	PM101005	p1_PM101005	316	265581	959759	20273	192	NZ_CP046903.1.gb	NZ_CP046903	NZ_CP046903.1	959759	0.06	6.30%
Ralstonia solanacearum FJAT-1458	FJAT-1458	plas1	1130828	2075659	956671	9468	193	NZ_CP016555.1.gb	NZ_CP016555	NZ_CP016555.1	956671	0.06	6.28%
Enterobacter hormaechei	SCEH020042	pQnrB4_020042	158836	328828	954633	14015	194	NZ_CP028537.1.gb	NZ_CP028537	NZ_CP028537.1	954633	0.06	6.26%
Cupriavidus taiwanensis		CBM2636_mp	164546	2848964	954265	21599	195	NZ_LT984814.1.gb	NZ_LT984814	NZ_LT984814.1	954265	0.06	6.26%
Sinorhizobium meliloti	USDA1157	psymA	382	1433621	951923	11452	196	NZ_CP021794.1.gb	NZ_CP021794	NZ_CP021794.1	951923	0.06	6.25%
Enterobacter cloacae		pEC-IMP	550	318782	949180	1955	197	NC_012555.1.gb	NC_012555	NC_012555.1	949180	0.06	6.23%
Pantoea sp. PSNIH2	NA	pPSP-100	1484157	165878	947234	6934	198	NZ_CP009868.gb	NZ_CP009868	NZ_CP009868	947234	0.06	6.22%
Ralstonia solanacearum CMR15	CMR15	CMR15_mp	859655	1959334	947039	3044	199	NC_017589.1.gb	NC_017589	NC_017589.1	947039	0.06	6.21%
Enterobacter cloacae		pEC-IMPQ	550	324503	946636	1956	200	NC_012556.1.gb	NC_012556	NC_012556.1	946636	0.06	6.21%
Aeromonas sp. ASNIH1	ASNIH1	pKPC-038c	1636606	77569	946540	13227	201	NZ_CP026230.1.gb	NZ_CP026230	NZ_CP026230.1	946540	0.06	6.21%
Ensifer adhaerens	Casida A	pCasidaAA	106592	1736943	946234	9279	202	NZ_CP015881.1.gb	NZ_CP015881	NZ_CP015881.1	946234	0.06	6.21%
Salmonella enterica subsp. enterica serovar Senftenberg	361154004	unnamed	749965	319930	945959	14253	203	NZ_CP029037.1.gb	NZ_CP029037	NZ_CP029037.1	945959	0.06	6.21%
Sinorhizobium meliloti	HM006	psymA	382	1484605	944854	11479	204	NZ_CP021830.1.gb	NZ_CP021830	NZ_CP021830.1	944854	0.06	6.20%
Aeromonas sp. ASNIH4	ASNIH4	pAER-f909	1636609	171236	943396	13221	205	NZ_CP026221.1.gb	NZ_CP026221	NZ_CP026221.1	943396	0.06	6.19%
Pseudomonas putida	KF715	pKF715B	303	276165	941855	5406	206	NZ_AP015031.1.gb	NZ_AP015031	NZ_AP015031.1	941855	0.06	6.18%
Sinorhizobium meliloti GR4	GR4	pRmeGR4c	1235461	1417907	939936	3732	207	NC_019848.2.gb	NC_019848	NC_019848.2	939936	0.06	6.17%
Aeromonas caviae	GSH8M-1	pGSH8M-1-1	648	153814	939672	5710	208	NZ_AP019196.1.gb	NZ_AP019196	NZ_AP019196.1	939672	0.06	6.17%
Ralstonia solanacearum	SL3755	unnamed	305	2006167	938488	11855	209	NZ_CP022783.1.gb	NZ_CP022783	NZ_CP022783.1	938488	0.06	6.16%
Escherichia coli	Ecol_316	pEC316_KPC	562	216226	936857	10403	210	NZ_CP018956.1.gb	NZ_CP018956	NZ_CP018956.1	936857	0.06	6.15%
Escherichia coli	Esco-36073cz	pEsco-36073cz	562	300958	935263	98	211	MG252895.gb	MG252895	MG252895	935263	0.06	6.14%
Klebsiella pneumoniae	D610	pD610-HI2	573	293643	935163	99	212	MG288680.gb	MG288680	MG288680	935163	0.06	6.14%
Sinorhizobium meliloti	CCMM B554 (FSM-MA)	pSymA	382	1422736	934885	10656	213	NZ_CP019585.1.gb	NZ_CP019585	NZ_CP019585.1	934885	0.06	6.14%
Sinorhizobium meliloti	USDA1157	psymB	382	1700327	934439	11453	214	NZ_CP021795.1.gb	NZ_CP021795	NZ_CP021795.1	934439	0.06	6.13%
Paraburkholderia sp. 7MH5	7MH5	unnamed1	2547399	682038	934296	17618	215	NZ_CP038152.1.gb	NZ_CP038152	NZ_CP038152.1	934296	0.06	6.13%
Ralstonia solanacearum	SL2729	unnamed	305	1989056	934243	11860	216	NZ_CP022793.1.gb	NZ_CP022793	NZ_CP022793.1	934243	0.06	6.13%
Ralstonia solanacearum	SL2330	unnamed	305	2001855	932491	11861	217	NZ_CP022795.1.gb	NZ_CP022795	NZ_CP022795.1	932491	0.06	6.12%
Methylobacterium aquaticum	MA-22A	pMaq22A_1p	270351	1571989	931477	5352	218	NZ_AP014705.1.gb	NZ_AP014705	NZ_AP014705.1	931477	0.06	6.11%

Organism	Strain	Plasmid	taxid	slen	score	order	rank	fileName	Accession ID	Accession Version	score_c	relative_score	relative_precentage_similarity
Sinorhizobium meliloti	USDA1021	psymA	382	1412628	930323	11457	219	NZ_CP021801.1.gb	NZ_CP021801	NZ_CP021801.1	930323	0.06	6.11%
Azospirillum brasilense	MTCC4035	p1	192	1930550	929531	15368	220	NZ_CP032322.1.gb	NZ_CP032322	NZ_CP032322.1	929531	0.06	6.10%
Ralstonia solanacearum CFBP2957	CFBP2957	RCFBPv3_mp	859656	2163376	929480	2361	221	NC_014309.1.gb	NC_014309	NC_014309.1	929480	0.06	6.10%
Salmonella enterica subsp. enterica serovar Concord	CFSAN018747	pGMI14-002_1	483687	444417	928038	13909	222	NZ_CP028197.1.gb	NZ_CP028197	NZ_CP028197.1	928038	0.06	6.09%
Sinorhizobium medicae WSM419	WSM419	pSMED02	366394	1245408	926238	1128	223	NC_009621.1.gb	NC_009621	NC_009621.1	926238	0.06	6.08%
Agrobacterium tumefaciens LBA4213 (Ach5)	LBA4213 (Ach5)	pAt	1435057	556485	926152	6488	224	NZ_CP007227.1.gb	NZ_CP007227	NZ_CP007227.1	926152	0.06	6.08%
Salmonella enterica subsp. enterica serovar Typhimurium	A54560	pSTm-A54650	90371	309406	924472	4364	225	NC_024983.1.gb	NC_024983	NC_024983.1	924472	0.06	6.07%
Agrobacterium tumefaciens	CFBP6626	pAtCFBP6626a	358	372686	924394	18195	226	NZ_CP039918.1.gb	NZ_CP039918	NZ_CP039918.1	924394	0.06	6.07%
Cupriavidus metallidurans CH34	CH34	pMOL28	266264	171459	922411	929	227	NC_007972.2.gb	NC_007972	NC_007972.2	922411	0.06	6.05%
Cupriavidus metallidurans	FDAARGOS_675	unnamed1	119219	171459	922411	20194	228	NZ_CP046330.1.gb	NZ_CP046330	NZ_CP046330.1	922411	0.06	6.05%
Pseudomonas putida UWC1	UWC1	pQBR44	1407054	91834	921599	9	229	CDLQ010000001.gb	CDLQ01000000	CDLQ010000001	921599	0.06	6.05%
Pseudomonas aeruginosa		pBS228	287	89147	921280	975	230	NC_008357.1.gb	NC_008357	NC_008357.1	921280	0.06	6.05%
Klebsiella pneumoniae	AR_0120	tig00000516_pilon	573	210045	920297	11483	231	NZ_CP021835.1.gb	NZ_CP021835	NZ_CP021835.1	920297	0.06	6.04%
Escherichia coli	170	RCS38_p	562	211452	920146	21643	232	NZ_LT985250.1.gb	NZ_LT985250	NZ_LT985250.1	920146	0.06	6.04%
Escherichia coli	AR435	unnamed5	562	187909	918271	14290	233	NZ_CP029118.1.gb	NZ_CP029118	NZ_CP029118.1	918271	0.06	6.03%
Vibrio cholerae		pNDM-116-14	666	354308	917886	20703	234	NZ_LN831184.1.gb	NZ_LN831184	NZ_LN831184.1	917886	0.06	6.02%
Citrobacter freundii		pNDM-CIT	546	288920	916956	3606	235	NC_019360.1.gb	NC_019360	NC_019360.1	916956	0.06	6.02%
Novosphingobium resinovorum	SA1	pSA2	158500	960805	916290	9660	236	NZ_CP017077.1.gb	NZ_CP017077	NZ_CP017077.1	916290	0.06	6.01%
Escherichia coli	100R	p100R	562	256260	916052	64	237	KY689633.gb	KY689633	KY689633	916052	0.06	6.01%
Salmonella enterica	SA20094620	pSA20094620.1	28901	298919	915420	14674	238	NZ_CP030186.1.gb	NZ_CP030186	NZ_CP030186.1	915420	0.06	6.01%
Klebsiella pneumoniae	C2343	pC2343-2-NDM	573	318848	914802	18157	239	NZ_CP039824.1.gb	NZ_CP039824	NZ_CP039824.1	914802	0.06	6.00%
Klebsiella pneumoniae	C2315	pC2315-2-NDM	573	317231	914802	18161	240	NZ_CP039829.1.gb	NZ_CP039829	NZ_CP039829.1	914802	0.06	6.00%
Azospirillum brasilense	MTCC4036	p1	192	2058394	914285	15375	241	NZ_CP032331.1.gb	NZ_CP032331	NZ_CP032331.1	914285	0.06	6.00%
Sinorhizobium meliloti	KH35c	psymA	382	1279723	913391	11477	242	NZ_CP021827.1.gb	NZ_CP021827	NZ_CP021827.1	913391	0.06	5.99%
Vibrio parahaemolyticus	VPS92	pVPS92-VEB	670	338538	910090	44	243	KU356480.gb	KU356480	KU356480	910090	0.06	5.97%
Enterobacter cloacae	20ES	p20ES-184	550	178976	909148	10	244	CM008910.gb	CM008910	CM008910	909148	0.06	5.97%
Enterobacter cloacae	20ES	p20ES-184	550	178976	909148	21748	245	PEHU01000006.gb	PEHU01000006	PEHU01000006	909148	0.06	5.97%
Sphingomonas sp. MM-1	MM-1	pISP0	745310	275840	908365	3879	246	NC_020542.1.gb	NC_020542	NC_020542.1	908365	0.06	5.96%
Ralstonia solanacearum	IBSBF 2570	unnamed	305	1949545	907666	13144	247	NZ_CP026091.1.gb	NZ_CP026091	NZ_CP026091.1	907666	0.06	5.96%
Escherichia coli	Ecol_732	pEC732_IMP14	562	186826	907503	8970	248	NZ_CP015139.1.gb	NZ_CP015139	NZ_CP015139.1	907503	0.06	5.96%
Enterobacter hormaechei	C15	pC15_001	158836	340181	905906	19034	249	NZ_CP042489.1.gb	NZ_CP042489	NZ_CP042489.1	905906	0.06	5.94%
Klebsiella variicola	13450	p13450-1	244366	344479	905896	13119	250	NZ_CP026014.1.gb	NZ_CP026014	NZ_CP026014.1	905896	0.06	5.94%
Enterobacter hormaechei	MRSN 17626	pMRVIM0813	158836	101071	905769	20656	251	NZ_JYGA02000003.gb	NZ_JYGA02000	NZ_JYGA02000003	905769	0.06	5.94%
Citrobacter freundii	R47	pR47-309	546	309536	904021	18408	252	NZ_CP040696.1.gb	NZ_CP040696	NZ_CP040696.1	904021	0.06	5.93%
Escherichia coli	APECA2	pJMA2	562	224630	903759	112	253	MH208235.gb	MH208235	MH208235	903759	0.06	5.93%
Cronobacter sakazakii	505108	p505108-MDR	28141	312880	903085	73	254	KY978628.gb	KY978628	KY978628	903085	0.06	5.93%
Burkholderia sp. PAMC 26561	PAMC 26561	unnamed2	1795043	793876	902448	8579	255	NZ_CP014309.1.gb	NZ_CP014309	NZ_CP014309.1	902448	0.06	5.92%
Cupriavidus campinensis	MJ1	unnamed1	151783	2786414	902095	19248	256	NZ_CP043441.1.gb	NZ_CP043441	NZ_CP043441.1	902095	0.06	5.92%
Klebsiella pneumoniae	13450	p13450-IMP	573	344478	901633	82	257	MF344564.gb	MF344564	MF344564	901633	0.06	5.92%
Ralstonia solanacearum	IBSBF1503	unnamed	305	2069788	901144	8020	258	NZ_CP012944.1.gb	NZ_CP012944	NZ_CP012944.1	901144	0.06	5.91%
Ralstonia solanacearum Po82	Po82	megaplasmid	1031711	1949172	899085	3038	259	NC_017575.1.gb	NC_017575	NC_017575.1	899085	0.06	5.90%
Klebsiella pneumoniae subsp. pneumoniae KPNIH27	KPNIH27	pKPN-262	1328324	338850	897741	6564	260	NZ_CP007734.1.gb	NZ_CP007734	NZ_CP007734.1	897741	0.06	5.89%
Sinorhizobium meliloti	KH35c	psymB	382	1624370	895557	11478	261	NZ_CP021828.1.gb	NZ_CP021828	NZ_CP021828.1	895557	0.06	5.88%
Paraburkholderia phymatum STM815	STM815	pBPHY01	391038	1904893	895168	1356	262	NC_010625.1.gb	NC_010625	NC_010625.1	895168	0.06	5.87%
Ralstonia solanacearum	UW163	plasmid	305	1932001	894643	8017	263	NZ_CP012940.1.gb	NZ_CP012940	NZ_CP012940.1	894643	0.06	5.87%
Azospirillum sp. TSH58	TSH58	TSH58_p02	664962	1910929	894285	11718	264	NZ_CP022367.1.gb	NZ_CP022367	NZ_CP022367.1	894285	0.06	5.87%
Novosphingobium resinovorum	SA1	pSA3	158500	354886	893756	9661	265	NZ_CP017078.1.gb	NZ_CP017078	NZ_CP017078.1	893756	0.06	5.87%
Enterobacter hormaechei	BW	unnamed1	158836	291244	891317	13554	266	NZ_CP027112.1.gb	NZ_CP027112	NZ_CP027112.1	891317	0.06	5.85%
Escherichia coli	GDZ13	pGD0503Z13	562	351717	890084	31	267	KR653209.gb	KR653209	KR653209	890084	0.06	5.84%
Ralstonia solanacearum	RS 488	unnamed	305	1999547	889907	11363	268	NZ_CP021653.1.gb	NZ_CP021653	NZ_CP021653.1	889907	0.06	5.84%
Ralstonia solanacearum	UY031	plasmid	305	1999545	889670	7932	269	NZ_CP012688.1.gb	NZ_CP012688	NZ_CP012688.1	889670	0.06	5.84%
Burkholderia sp. PAMC 26561	PAMC 26561	unnamed1	1795043	793818	888685	8578	270	NZ_CP014308.1.gb	NZ_CP014308	NZ_CP014308.1	888685	0.06	5.83%
Enterobacter hormaechei	BW	unnamed2	158836	291254	888452	12090	271	NZ_CP023570.1.gb	NZ_CP023570	NZ_CP023570.1	888452	0.06	5.83%
Klebsiella michiganensis E718	E718	pKOX_R1	1191061	353865	888133	3164	272	NC_018107.1.gb	NC_018107	NC_018107.1	888133	0.06	5.83%
Ralstonia solanacearum	CQPS-1	plasmid	305	2061090	887803	9621	273	NZ_CP016915.1.gb	NZ_CP016915	NZ_CP016915.1	887803	0.06	5.83%
Cupriavidus metallidurans CH34	CH34	pMOL28	266264	171461	887458	730	274	NC_006525.1.gb	NC_006525	NC_006525.1	887458	0.06	5.82%
Sinorhizobium meliloti	M270	plasmid	382	1454983	887396	11466	275	NZ_CP021813.1.gb	NZ_CP021813	NZ_CP021813.1	887396	0.06	5.82%
Pseudomonas fluorescens HK44	HK44	pUTK21	1042209	116775	886990	5324	276	NZ_AFOY02000029.gb	NZ_AFOY02000	NZ_AFOY02000029	886990	0.06	5.82%
Ralstonia solanacearum	RS 489	unnamed	305	1999547	886921	11443	277	NZ_CP021767.1.gb	NZ_CP021767	NZ_CP021767.1	886921	0.06	5.82%
Enterobacter cloacae	AR_0154	unnamed4	550	315949	886118	14487	278	NZ_CP029717.1.gb	NZ_CP029717	NZ_CP029717.1	886118	0.06	5.82%
Enterobacter hormaechei subsp. steigerwaltii		pC309-p2	299766	183339	885813	21720	279	NZ_LT991956.1.gb	NZ_LT991956	NZ_LT991956.1	885813	0.06	5.81%
Salmonella enterica subsp. enterica serovar Typhimurium	BL10	p220k	90371	220023	883919	12897	280	NZ_CP025340.1.gb	NZ_CP025340	NZ_CP025340.1	883919	0.06	5.80%
Ralstonia solanacearum OE1-1	OE1-1	unnamed	1429399	1936579	883679	6893	281	NZ_CP009763.1.gb	NZ_CP009763	NZ_CP009763.1	883679	0.06	5.80%
Klebsiella variicola	6TM	p6TM-742	244366	741861	883564	21745	282	NZ_PEHS01000013.gb	NZ_PEHS01000	NZ_PEHS01000013	883564	0.06	5.80%
Shewanella algae	KC-Na-R1	pKC-Na-R1	38313	167144	883394	15799	283	NZ_CP033574.1.gb	NZ_CP033574	NZ_CP033574.1	883394	0.06	5.80%
Salmonella enterica subsp. enterica serovar Dublin	CVM 22429	serovar	98360	329264	883275	15410	284	NZ_CP032397.1.gb	NZ_CP032397	NZ_CP032397.1	883275	0.06	5.80%
Phytobacter ursingii	CAV1151	pCAV1151-296	1972431	295619	880243	7599	285	NZ_CP011601.1.gb	NZ_CP011601	NZ_CP011601.1	880243	0.06	5.78%
Azospirillum sp. M2T2B2	M2T2B2	unnamed1	682998	919301	879329	14537	286	NZ_CP029830.1.gb	NZ_CP029830	NZ_CP029830.1	879329	0.06	5.77%
Enterobacter asburiae	AMA 497	pOXA436	61645	314137	878962	65	287	KY863418.gb	KY863418	KY863418	878962	0.06	5.77%
Sinorhizobium meliloti	USDA1106	psymA	382	1363628	878552	11455	288	NZ_CP021798.1.gb	NZ_CP021798	NZ_CP021798.1	878552	0.06	5.77%
Klebsiella pneumoniae	WCHKP34	pIMP4_LL34	573	260970	877777	13109	289	NZ_CP025964.2.gb	NZ_CP025964	NZ_CP025964.2	877777	0.06	5.76%
Klebsiella aerogenes	G7	pGPN1	548	174066	877481	7570	290	NZ_CP011540.1.gb	NZ_CP011540	NZ_CP011540.1	877481	0.06	5.76%
Salmonella enterica subsp. enterica serovar Heidelberg	111	Heidelberg	611	165791	877442	3392	291	NC_019121.1.gb	NC_019121	NC_019121.1	877442	0.06	5.76%
Serratia marcescens	CAV1761	pCAV1761-205	615	204825	876341	14415	292	NZ_CP029448.1.gb	NZ_CP029448	NZ_CP029448.1	876341	0.06	5.75%

Organism	Strain	Plasmid	taxid	slen	score	order	rank	fileName	Accession ID	Accession Version	score_c	relative_score	relative_precentage_similarity
Salmonella enterica subsp. enterica	USDA-ARS-USMARC-60984	pSJO-60984	59201	266480	876095	12887	293	NZ_CP025277.1.gb	NZ_CP025277	NZ_CP025277.1	876095	0.06	5.75%
Enterobacter hormaechei subsp. xiangfangensis	OSUKPC4_L	pOSUKPC4	1296536	351806	875689	12793	294	NZ_CP024910.1.gb	NZ_CP024910	NZ_CP024910.1	875689	0.06	5.75%
Enterobacter hormaechei subsp. xiangfangensis	OSUVMCKPC4-2	pOSUEC_D	1296536	354256	875689	14357	295	NZ_CP029248.1.gb	NZ_CP029248	NZ_CP029248.1	875689	0.06	5.75%
Cupriavidus sp. USMAA2-4	USMAA2-4	unnamed1	876364	544877	875537	9899	296	NZ_CP017750.1.gb	NZ_CP017750	NZ_CP017750.1	875537	0.06	5.75%
Sinorhizobium meliloti SM11	SM11	pSmeSM11d	707241	1632395	875392	2931	297	NC_017326.1.gb	NC_017326	NC_017326.1	875392	0.06	5.74%
Salmonella sp.	Sa4	pSa4-CIP	599	237130	874946	108	298	MG874042.gb	MG874042	MG874042	874946	0.06	5.74%
Escherichia coli	KSC207	pMRGN207	562	277790	874895	10639	299	NZ_CP019559.1.gb	NZ_CP019559	NZ_CP019559.1	874895	0.06	5.74%
Aeromonas salmonicida subsp. salmonicida A449	A449	4	382245	166749	874712	1089	300	NC_009349.1.gb	NC_009349	NC_009349.1	874712	0.06	5.74%
Klebsiella quasipneumoniae	A708	pA708-1	1463165	238703	874679	13273	301	NZ_CP026369.1.gb	NZ_CP026369	NZ_CP026369.1	874679	0.06	5.74%
Ralstonia solanacearum	SL3730	unnamed	305	1969688	874119	11856	302	NZ_CP022785.1.gb	NZ_CP022785	NZ_CP022785.1	874119	0.06	5.74%
Sinorhizobium meliloti	AK21	pSymA	382	1506823	873316	13334	303	NZ_CP026526.1.gb	NZ_CP026526	NZ_CP026526.1	873316	0.06	5.73%
Klebsiella pneumoniae	18ES	p18ES-342	573	332674	873144	21746	304	PEGY01000003.gb	PEGY01000003	PEGY01000003	873144	0.06	5.73%
Aeromonas salmonicida subsp. salmonicida	01-B522	pAsa4b	29491	181933	872740	34	305	KT033469.gb	KT033469	KT033469	872740	0.06	5.73%
Nocardia farcinica	NCTC11134	2	37329	2657929	872651	20716	306	NZ_LN868939.1.gb	NZ_LN868939	NZ_LN868939.1	872651	0.06	5.73%
Serratia marcescens	CAV1492	pCAV1492-199	615	199444	872291	7634	307	NZ_CP011641.1.gb	NZ_CP011641	NZ_CP011641.1	872291	0.06	5.72%
Escherichia coli	83	RCS1TR83_p	562	174073	872067	21613	308	NZ_LT985220.1.gb	NZ_LT985220	NZ_LT985220.1	872067	0.06	5.72%
Escherichia coli	89	RCS2TR89_p	562	175317	872067	21618	309	NZ_LT985225.1.gb	NZ_LT985225	NZ_LT985225.1	872067	0.06	5.72%
Rhizobium leguminosarum	Vaf10	unnamed1	384	1254530	871602	9354	310	NZ_CP016287.1.gb	NZ_CP016287	NZ_CP016287.1	871602	0.06	5.72%
Klebsiella aerogenes	KA_P10_L5_03.19	pIMPIncH12_334kb	548	333880	871508	19430	311	NZ_CP044215.1.gb	NZ_CP044215	NZ_CP044215.1	871508	0.06	5.72%
Klebsiella pneumoniae	18ES	p18ES-342	573	332674	871345	5858	312	NZ_CM008881.gb	NZ_CM008881	NZ_CM008881	871345	0.06	5.72%
Ralstonia solanacearum	YC40-M	complete	305	1907366	871267	9268	313	NZ_CP015851.1.gb	NZ_CP015851	NZ_CP015851.1	871267	0.06	5.72%
Escherichia coli	548	RCS24TR548_p	562	175038	870667	21615	314	NZ_LT985222.1.gb	NZ_LT985222	NZ_LT985222.1	870667	0.06	5.71%
Klebsiella pneumoniae		CNR48	573	319209	870576	21732	315	NZ_LT994835.1.gb	NZ_LT994835	NZ_LT994835.1	870576	0.06	5.71%
Rhodococcus sp. WAY2	WAY2	pRWAY01	2663121	991117	870220	20227	316	NZ_CP046573.1.gb	NZ_CP046573	NZ_CP046573.1	870220	0.06	5.71%
Klebsiella pneumoniae	AP8555	pAP855	573	357837	868784	16523	317	NZ_CP035384.1.gb	NZ_CP035384	NZ_CP035384.1	868784	0.06	5.70%
Azospirillum brasilense	MTCC4039	p1	192	1845095	868585	15388	318	NZ_CP032346.1.gb	NZ_CP032346	NZ_CP032346.1	868585	0.06	5.70%
Klebsiella pneumoniae	Kp55	pKp55	573	215528	866688	70	319	KY887594.gb	KY887594	KY887594	866688	0.06	5.69%
Novosphingobium sp. THN1	THN1	pTHN	1016987	1161559	866086	13980	320	NZ_CP028348.1.gb	NZ_CP028348	NZ_CP028348.1	866086	0.06	5.68%
Enterobacter cloacae subsp. cloacae	NUH14_ECL028	pNUH14_ECL028_1	336306	251286	865932	5720	321	NZ_AP019384.1.gb	NZ_AP019384	NZ_AP019384.1	865932	0.06	5.68%
Paraburkholderia hospita	mHSR1	pmHSR1_P	169430	1235162	864982	12803	322	NZ_CP024940.1.gb	NZ_CP024940	NZ_CP024940.1	864982	0.06	5.68%
Salmonella enterica subsp. enterica serovar Typhimurium	ST313	incHI2	90371	300375	864531	77	323	LN794248.gb	LN794248	LN794248	864531	0.06	5.67%
Ralstonia solanacearum Rs-09-161	Rs-09-161	unnamed1	1394180	1957122	861224	5855	324	NZ_CM002758.gb	NZ_CM002758	NZ_CM002758	861224	0.06	5.65%
Methylomonas sp. DH-1	DH-1	plasmid	1727196	277875	861140	8601	325	NZ_CP014361.1.gb	NZ_CP014361	NZ_CP014361.1	861140	0.06	5.65%
Klebsiella quasipneumoniae	CAV1947	pKPC_CAV1947-412	1463165	412382	861121	14410	326	NZ_CP029442.1.gb	NZ_CP029442	NZ_CP029442.1	861121	0.06	5.65%
Sinorhizobium meliloti	T073	psymA	382	1423499	859959	11460	327	NZ_CP021805.1.gb	NZ_CP021805	NZ_CP021805.1	859959	0.06	5.64%
Halomonas sp. PA16-9	PA16-9	p_unnamed1	2576841	1833961	859805	18345	328	NZ_CP040452.1.gb	NZ_CP040452	NZ_CP040452.1	859805	0.06	5.64%
Klebsiella pneumoniae subsp. pneumoniae	234-12	pKpn23412-362	72407	361964	859727	7510	329	NZ_CP011314.1.gb	NZ_CP011314	NZ_CP011314.1	859727	0.06	5.64%
Leclercia sp. LSNIH3	LSNIH3	pLEC-5e18	1920116	195394	859042	13283	330	NZ_CP026390.1.gb	NZ_CP026390	NZ_CP026390.1	859042	0.06	5.64%
Pseudomonas putida	HS1	pDK1	303	128921	858699	2315	331	NC_014124.1.gb	NC_014124	NC_014124.1	858699	0.06	5.64%
Ensifer adhaerens	Casida A	pCasidaAB	106592	1459374	857541	9280	332	NZ_CP015882.1.gb	NZ_CP015882	NZ_CP015882.1	857541	0.06	5.63%
Klebsiella pneumoniae	C1398	pVir-CR-hvKP-C1398	573	274540	857389	16154	333	NZ_CP034421.1.gb	NZ_CP034421	NZ_CP034421.1	857389	0.06	5.63%
Sinorhizobium meliloti RU11	1	pSymB	1401243	1686813	857363	11234	334	NZ_CP021218.1.gb	NZ_CP021218	NZ_CP021218.1	857363	0.06	5.63%
Pseudomonas syringae pv. avii		PP4	663959	77492	856251	21488	335	NZ_LT963406.1.gb	NZ_LT963406	NZ_LT963406.1	856251	0.06	5.62%
Sphingobium herbicidovorans	MH	pMSHV	76947	959196	856164	10965	336	NZ_CP020539.1.gb	NZ_CP020539	NZ_CP020539.1	856164	0.06	5.62%
Klebsiella pneumoniae		unnamed1	573	221606	855739	12373	337	NZ_CP024192.1.gb	NZ_CP024192	NZ_CP024192.1	855739	0.06	5.62%
Klebsiella pneumoniae	INF158	unnamed1	573	221606	855739	12630	338	NZ_CP024522.1.gb	NZ_CP024522	NZ_CP024522.1	855739	0.06	5.62%
Klebsiella pneumoniae	INF157	unnamed1	573	221606	855739	12636	339	NZ_CP024529.1.gb	NZ_CP024529	NZ_CP024529.1	855739	0.06	5.62%
Klebsiella pneumoniae	19051	p19051-IMP	573	316843	855729	83	340	MF344565.gb	MF344565	MF344565	855729	0.06	5.62%
Paraburkholderia caribensis	MWAP64	1	75105	2011268	854040	8080	341	NZ_CP013104.1.gb	NZ_CP013104	NZ_CP013104.1	854040	0.06	5.60%
Klebsiella pneumoniae subsp. pneumoniae	H11	H11	72407	284628	853592	16	342	CP013215.gb	CP013215	CP013215	853592	0.06	5.60%
Enterobacter kobei	C16	pC16_001	208224	275807	852977	19092	343	NZ_CP042579.1.gb	NZ_CP042579	NZ_CP042579.1	852977	0.06	5.60%
Salmonella enterica subsp. enterica serovar Lomita	SL131	pSL131_IncHI2	1160757	273923	852707	111	344	MH105051.gb	MH105051	MH105051	852707	0.06	5.60%
Escherichia coli	CFS3313	pCFS3313-1	562	155172	852491	13499	345	NZ_CP026940.1.gb	NZ_CP026940	NZ_CP026940.1	852491	0.06	5.59%
Sinorhizobium meliloti 2011	2011	pSymA	1286640	1352561	852473	3872	346	NC_020527.1.gb	NC_020527	NC_020527.1	852473	0.06	5.59%
Azospirillum brasilense	Az39	AbAZ39_p1	192	1901707	851805	6576	347	NZ_CP007794.1.gb	NZ_CP007794	NZ_CP007794.1	851805	0.06	5.59%
Enterobacter hormaechei	MS7884A	pMS7884A	158836	330060	851766	11771	348	NZ_CP022533.1.gb	NZ_CP022533	NZ_CP022533.1	851766	0.06	5.59%
Escherichia coli	P2-3	pP2-3T	562	392275	851759	96	349	MG014722.gb	MG014722	MG014722	851759	0.06	5.59%
Sinorhizobium americanum CCGM7	CCGM7	C	1408224	2249899	851738	8068	350	NZ_CP013054.1.gb	NZ_CP013054	NZ_CP013054.1	851738	0.06	5.59%
Enterobacter hormaechei subsp. steigerwaltii	ME-1	pME-1a	299766	276520	851179	18800	351	NZ_CP041734.1.gb	NZ_CP041734	NZ_CP041734.1	851179	0.06	5.59%
Escherichia coli	WCHEC050613	pMCR_WCHEC050613	562	289260	850608	10534	352	NZ_CP019214.3.gb	NZ_CP019214	NZ_CP019214.3	850608	0.06	5.58%
Salmonella enterica subsp. enterica serovar Lomita	SL131	pSL131_IncA/C-IncX3	1160757	216895	850524	110	353	MH105050.gb	MH105050	MH105050	850524	0.06	5.58%
Klebsiella pneumoniae	12208	p12208-IMP	573	323333	850259	80	354	MF344562.gb	MF344562	MF344562	850259	0.06	5.58%
Klebsiella pneumoniae	KP1766	KP1766_p1	573	205953	850198	12854	355	NZ_CP025147.1.gb	NZ_CP025147	NZ_CP025147.1	850198	0.06	5.58%
Klebsiella pneumoniae	Kp15	pENVA	573	253984	850056	20	356	HG918041.gb	HG918041	HG918041	850056	0.06	5.58%
Agrobacterium tumefaciens	CFBP6625	pAtCFBP6625b	358	386925	850049	18192	357	NZ_CP039913.1.gb	NZ_CP039913	NZ_CP039913.1	850049	0.06	5.58%
Klebsiella pneumoniae	A708	pA708-IMP	573	238703	850010	85	358	MF344567.gb	MF344567	MF344567	850010	0.06	5.58%
Enterobacter cloacae	30860	p30860-HI2	550	249241	849099	90	359	MF344580.gb	MF344580	MF344580	849099	0.06	5.57%
Klebsiella pneumoniae	NR5632	NR5632_p1	573	204123	848921	12852	360	NZ_CP025144.1.gb	NZ_CP025144	NZ_CP025144.1	848921	0.06	5.57%
Klebsiella pneumoniae	KP1768	KP1768_p1	573	204734	848131	12850	361	NZ_CP025141.1.gb	NZ_CP025141	NZ_CP025141.1	848131	0.06	5.57%
Salmonella enterica subsp. enterica serovar Typhimurium	MU1	pIMP4-SEM1	90371	339962	847723	52	362	KX810825.gb	KX810825	KX810825	847723	0.06	5.56%
Shewanella bicestrii	JAB-1	pSHE-CTX-M	2018305	193338	847155	11713	363	NZ_CP022359.1.gb	NZ_CP022359	NZ_CP022359.1	847155	0.06	5.56%
Kluyvera cryocrescens	BO64W	pKC-BO-N1-VIM	580	170352	846547	97	364	MG228427.gb	MG228427	MG228427	846547	0.06	5.56%
Escherichia coli	A1_180	unnamed1	562	317051	846460	18332	365	NZ_CP040382.1.gb	NZ_CP040382	NZ_CP040382.1	846460	0.06	5.55%
Mesorhizobium loti NZP2037	NZP2037	pML2037	935546	474413	846228	9325	366	NZ_CP016080.1.gb	NZ_CP016080	NZ_CP016080.1	846228	0.06	5.55%

Organism	Strain	Plasmid	taxid	slen	score	order	rank	fileName	Accession ID	Accession Version	score_c	relative_score	relative_precentage_similarity
Escherichia coli	SF-088	pSF-088-1	562	149683	844940	7909	367	NZ_CP012636.1.gb	NZ_CP012636	NZ_CP012636.1	844940	0.06	5.54%
Ralstonia solanacearum	KACC 10722	plasmid	305	1883432	844879	8778	368	NZ_CP014703.1.gb	NZ_CP014703	NZ_CP014703.1	844879	0.06	5.54%
Ralstonia solanacearum	T95	unnamed	305	1884412	844879	11844	369	NZ_CP022762.1.gb	NZ_CP022762	NZ_CP022762.1	844879	0.06	5.54%
Ralstonia solanacearum	SL2064	unnamed	305	1884392	844879	11863	370	NZ_CP022799.1.gb	NZ_CP022799	NZ_CP022799.1	844879	0.06	5.54%
Ralstonia solanacearum	T11	unnamed	305	1883805	844873	11852	371	NZ_CP022777.1.gb	NZ_CP022777	NZ_CP022777.1	844873	0.06	5.54%
Ralstonia solanacearum	T51	unnamed	305	1883756	844801	11849	372	NZ_CP022771.1.gb	NZ_CP022771	NZ_CP022771.1	844801	0.06	5.54%
Klebsiella pneumoniae	WCGKP294	pWCGKP294-2	573	230910	842977	20234	373	NZ_CP046614.1.gb	NZ_CP046614	NZ_CP046614.1	842977	0.06	5.53%
Escherichia coli	EC-129	pEC129_1	562	211718	842497	17652	374	NZ_CP038454.1.gb	NZ_CP038454	NZ_CP038454.1	842497	0.06	5.53%
Pseudomonas amygdali pv. lachrymans	M301315	pMPPla107	629260	971889	842130	14939	375	NZ_CP031226.1.gb	NZ_CP031226	NZ_CP031226.1	842130	0.06	5.53%
Enterobacter sp. CRENT-193	CRENT-193	pCRENT-193_1	2051905	298989	841515	12743	376	NZ_CP024813.1.gb	NZ_CP024813	NZ_CP024813.1	841515	0.06	5.52%
Enterobacter hormaechei	L51	pEHZJ1	158836	343918	841471	15648	377	NZ_CP033103.1.gb	NZ_CP033103	NZ_CP033103.1	841471	0.06	5.52%
Ralstonia solanacearum	T101	unnamed	305	1988531	841467	11842	378	NZ_CP022758.1.gb	NZ_CP022758	NZ_CP022758.1	841467	0.06	5.52%
Klebsiella pneumoniae		pR55	573	170810	841465	2781	379	NC_016976.1.gb	NC_016976	NC_016976.1	841465	0.06	5.52%
Ralstonia solanacearum Rs-10-244	Rs-10-244	unnamed1	1457195	1996201	840937	5854	380	NZ_CM002756.gb	NZ_CM002756	NZ_CM002756	840937	0.06	5.52%
Ralstonia solanacearum PSI07	PSI07	mpPSI07	859657	2085000	840708	2362	381	NC_014310.1.gb	NC_014310	NC_014310.1	840708	0.06	5.52%
Salmonella enterica subsp. enterica serovar Heidelberg str. N418	N418	pCFSAN000405_01	1030005	190923	840620	6791	382	NZ_CP009409.2.gb	NZ_CP009409	NZ_CP009409.2	840620	0.06	5.52%
Rhodobacter sphaeroides	ATCC 17025	pRSPA01	349102	877879	840124	1099	383	NC_009429.gb	NC_009429	NC_009429	840124	0.06	5.51%
Enterobacter hormaechei subsp. steigerwaltii		pC309-VIM4	299766	254277	838494	21719	384	NZ_LT991955.1.gb	NZ_LT991955	NZ_LT991955.1	838494	0.06	5.50%
Aeromonas salmonicida subsp. salmonicida	SHY16-3432	pAsa5-3432	29491	180043	837862	17611	385	NZ_CP038103.1.gb	NZ_CP038103	NZ_CP038103.1	837862	0.05	5.50%
Burkholderia sp. KK1	KK1	pkk1	1855726	1563050	836882	9311	386	NZ_CP016002.1.gb	NZ_CP016002	NZ_CP016002.1	836882	0.05	5.49%
Ralstonia solanacearum	T82	unnamed	305	1988569	836828	11845	387	NZ_CP022764.1.gb	NZ_CP022764	NZ_CP022764.1	836828	0.05	5.49%
Salmonella enterica subsp. enterica	08-00436	pSE08-00436-1	59201	264914	836764	10937	388	NZ_CP020493.1.gb	NZ_CP020493	NZ_CP020493.1	836764	0.05	5.49%
Ralstonia solanacearum		unnamed	305	2001669	836385	11902	389	NZ_CP023015.1.gb	NZ_CP023015	NZ_CP023015.1	836385	0.05	5.49%
Agrobacterium tumefaciens	CFBP6625	pAtCFBP6625a	358	385084	836051	18191	390	NZ_CP039912.1.gb	NZ_CP039912	NZ_CP039912.1	836051	0.05	5.49%
Klebsiella pneumoniae subsp. pneumoniae		pKPM502	72407	250351	836048	15103	391	NZ_CP031736.1.gb	NZ_CP031736	NZ_CP031736.1	836048	0.05	5.49%
Burkholderia sp. THE68	THE68	BTHE68_p1	758782	1527330	835234	5827	392	NZ_AP022319.1.gb	NZ_AP022319	NZ_AP022319.1	835234	0.05	5.48%
Azospirillum brasilense Sp245	Sp245	Sp245_p02	1064539	1776336	834922	11673	393	NZ_CP022254.1.gb	NZ_CP022254	NZ_CP022254.1	834922	0.05	5.48%
Ralstonia solanacearum	T42	unnamed	305	1781111	834850	11850	394	NZ_CP022773.1.gb	NZ_CP022773	NZ_CP022773.1	834850	0.05	5.48%
Pseudomonas aeruginosa	AZPAE15042	pIHMA87	287	185168	832585	18671	395	NZ_CP041355.1.gb	NZ_CP041355	NZ_CP041355.1	832585	0.05	5.46%
Pseudomonas aeruginosa VRFPA03	VRFPA03	MUM024	1350465	39344	832483	7	396	ATNK01000027.gb	ATNK01000027	ATNK01000027	832483	0.05	5.46%
Ralstonia solanacearum	SL2312	unnamed	305	1988567	832303	11862	397	NZ_CP022797.1.gb	NZ_CP022797	NZ_CP022797.1	832303	0.05	5.46%
Klebsiella sp. LY	LY	unnamed3	2015795	316557	831592	11737	398	NZ_CP022441.1.gb	NZ_CP022441	NZ_CP022441.1	831592	0.05	5.46%
Ralstonia solanacearum	UW386	pUW386	305	1830097	831272	18018	399	NZ_CP039340.1.gb	NZ_CP039340	NZ_CP039340.1	831272	0.05	5.46%
Ralstonia solanacearum	SL3175	unnamed	305	1986284	830794	11858	400	NZ_CP022789.1.gb	NZ_CP022789	NZ_CP022789.1	830794	0.05	5.45%
Ralstonia solanacearum	T98	unnamed	305	1986282	830747	11843	401	NZ_CP022760.1.gb	NZ_CP022760	NZ_CP022760.1	830747	0.05	5.45%
Salmonella enterica subsp. enterica serovar Senftenberg	775W	pSSE-ATCC-43845	28150	341373	830627	9588	402	NZ_CP016838.1.gb	NZ_CP016838	NZ_CP016838.1	830627	0.05	5.45%
Sinorhizobium fredii	CCBAU 45436	pSF45436b	1128331	1961287	830494	14349	403	NZ_CP029232.1.gb	NZ_CP029232	NZ_CP029232.1	830494	0.05	5.45%
Klebsiella pneumoniae subsp. pneumoniae	KP4898	pIncAC-KP4898	72407	156252	830412	66	404	KY882285.gb	KY882285	KY882285	830412	0.05	5.45%
Enterobacter cloacae		I	550	177037	829547	21439	405	NZ_LT882699.1.gb	NZ_LT882699	NZ_LT882699.1	829547	0.05	5.44%
Pseudomonas amygdali pv. lachrymans	M301315	pMPPla107	629260	967397	829495	5851	406	NZ_CM000959.gb	NZ_CM000959	NZ_CM000959	829495	0.05	5.44%
Paracoccus pantotrophus	DSM 2944	pPAN2	82367	535332	829207	19515	407	NZ_CP044425.1.gb	NZ_CP044425	NZ_CP044425.1	829207	0.05	5.44%
Klebsiella pneumoniae subsp. pneumoniae Kp13	Kp13	pKP13f	1123862	295493	828170	5886	408	NZ_CP004000.1.gb	NZ_CP004000	NZ_CP004000.1	828170	0.05	5.43%
Proteus mirabilis	AR_0155	tig00000123	584	214441	828028	11394	409	NZ_CP021695.1.gb	NZ_CP021695	NZ_CP021695.1	828028	0.05	5.43%
Klebsiella pneumoniae	WCHKP020030	pOXA1_020030	573	288222	827890	14147	410	NZ_CP028791.1.gb	NZ_CP028791	NZ_CP028791.1	827890	0.05	5.43%
Pseudomonas putida	12969	p12969-2	303	109708	827657	59	411	KY270855.gb	KY270855	KY270855	827657	0.05	5.43%
Klebsiella pneumoniae	KSB1_9D	unnamed1	573	187611	825631	12642	412	NZ_CP024536.1.gb	NZ_CP024536	NZ_CP024536.1	825631	0.05	5.42%
Klebsiella pneumoniae	INF163	unnamed1	573	187611	825631	12652	413	NZ_CP024550.1.gb	NZ_CP024550	NZ_CP024550.1	825631	0.05	5.42%
Klebsiella pneumoniae	INF278	unnamed1	573	187611	825631	12664	414	NZ_CP024564.1.gb	NZ_CP024564	NZ_CP024564.1	825631	0.05	5.42%
Klebsiella pneumoniae	INF274	unnamed1	573	187611	825631	12670	415	NZ_CP024571.1.gb	NZ_CP024571	NZ_CP024571.1	825631	0.05	5.42%
Microvirga ossetica	V5	unnamed1	1882682	1343367	825057	9496	416	NZ_CP016617.1.gb	NZ_CP016617	NZ_CP016617.1	825057	0.05	5.41%
Pseudomonas putida	MT53	pWW53	303	107929	825007	963	417	NC_008275.1.gb	NC_008275	NC_008275.1	825007	0.05	5.41%
Klebsiella pneumoniae subsp. pneumoniae	SC-7	pSC7-vir	72407	236809	823606	14719	418	NZ_CP030270.1.gb	NZ_CP030270	NZ_CP030270.1	823606	0.05	5.40%
Proteus mirabilis	AR_0156	unitig_1	584	180262	823535	11493	419	NZ_CP021853.1.gb	NZ_CP021853	NZ_CP021853.1	823535	0.05	5.40%
Enterobacter hormaechei	20710	pIMP-20710	158836	286652	822021	14642	420	NZ_CP030080.1.gb	NZ_CP030080	NZ_CP030080.1	822021	0.05	5.39%
Burkholderia sp.	RPE67	p1	758796	1438033	821760	5338	421	NZ_AP014579.gb	NZ_AP014579	NZ_AP014579	821760	0.05	5.39%
Salmonella enterica subsp. enterica serovar 1,4,[5],12:i:-	PNCS014876	p15-0756.1	2583588	249601	821317	18168	422	NZ_CP039857.1.gb	NZ_CP039857	NZ_CP039857.1	821317	0.05	5.39%
Klebsiella pneumoniae	13ARS-GMH0099	p13ARS_GMH0099	573	273158	820066	21260	423	NZ_LR697099.1.gb	NZ_LR697099	NZ_LR697099.1	820066	0.05	5.38%
Klebsiella pneumoniae	INF164	unnamed1	573	217685	820003	12658	424	NZ_CP024557.1.gb	NZ_CP024557	NZ_CP024557.1	820003	0.05	5.38%
Klebsiella pneumoniae	PMK1	pPMK1-NDM	573	304526	819606	6642	425	NZ_CP008933.1.gb	NZ_CP008933	NZ_CP008933.1	819606	0.05	5.38%
Rhizorhabdus dicambivorans	Ndbn-20	p2	1850238	256400	819598	12046	426	NZ_CP023451.1.gb	NZ_CP023451	NZ_CP023451.1	819598	0.05	5.38%
Klebsiella pneumoniae	CDC 0106	unnamed1	573	334957	818070	11795	427	NZ_CP022612.1.gb	NZ_CP022612	NZ_CP022612.1	818070	0.05	5.37%
Klebsiella pneumoniae	C51	pC51_002	573	208956	817604	19029	428	NZ_CP042483.1.gb	NZ_CP042483	NZ_CP042483.1	817604	0.05	5.37%
Klebsiella pneumoniae	KPN528	pKPN528-1	573	292471	817160	11056	429	NZ_CP020854.1.gb	NZ_CP020854	NZ_CP020854.1	817160	0.05	5.36%
Enterobacteriaceae bacterium ENNIH2	ENNIH2	pENT-812c	1920109	171306	817133	13196	430	NZ_CP026189.1.gb	NZ_CP026189	NZ_CP026189.1	817133	0.05	5.36%
Ralstonia solanacearum	T12	unnamed	305	1988373	816105	11851	431	NZ_CP022775.1.gb	NZ_CP022775	NZ_CP022775.1	816105	0.05	5.36%
Acinetobacter johnsonii XBB1	XBB1	pXBB1-9	1242245	398857	815929	7127	432	NZ_CP010351.1.gb	NZ_CP010351	NZ_CP010351.1	815929	0.05	5.35%
Citrobacter sp. NMI7904_11	NMI7904_11	pCTEL-2	2653932	235057	815627	19880	433	NZ_CP045203.1.gb	NZ_CP045203	NZ_CP045203.1	815627	0.05	5.35%
Citrobacter freundii	AR_0116	unnamed2	546	137578	814047	15286	434	NZ_CP032180.1.gb	NZ_CP032180	NZ_CP032180.1	814047	0.05	5.34%
Klebsiella pneumoniae		pKP04VIM	573	274659	813405	42	435	KU318421.gb	KU318421	KU318421	813405	0.05	5.34%
Acidiphilium multivorum AIU301	AIU301	pACMV1	926570	271573	813387	2498	436	NC_015178.1.gb	NC_015178	NC_015178.1	813387	0.05	5.34%
Enterobacter hormaechei	CAV1668	pCAV1668-85	158836	85187	813311	7584	437	NZ_CP011583.1.gb	NZ_CP011583	NZ_CP011583.1	813311	0.05	5.34%
Leclercia adecarboxylata	E61	pE61_002	83655	178161	813142	19039	438	NZ_CP042495.1.gb	NZ_CP042495	NZ_CP042495.1	813142	0.05	5.34%
Rhizobium gallicum bv. gallicum R602	NA	pRgalR602c	1418105	2466951	811387	6413	439	NZ_CP006880.gb	NZ_CP006880	NZ_CP006880	811387	0.05	5.32%
Enterobacter hormaechei	CAV1311	pKPC_CAV1311	158836	90452	809673	7576	440	NZ_CP011571.1.gb	NZ_CP011571	NZ_CP011571.1	809673	0.05	5.31%

Organism	Strain	Plasmid	taxid	slen	score	order	rank	fileName	Accession ID	Accession Version	score_c	relative_score	relative_precentage_similarity
Enterobacter hormaechei	CAV1411	pKPC_CAV1411	158836	90452	809673	7582	441	NZ_CP011580.1.gb	NZ_CP011580	NZ_CP011580.1	809673	0.05	5.31%
Enterobacter hormaechei	CAV1669	pKPC_CAV1669	158836	90452	809673	7640	442	NZ_CP011649.1.gb	NZ_CP011649	NZ_CP011649.1	809673	0.05	5.31%
Enterobacter hormaechei	CAV1176	pKPC_CAV1176	158836	90452	809673	7650	443	NZ_CP011661.1.gb	NZ_CP011661	NZ_CP011661.1	809673	0.05	5.31%
Klebsiella pneumoniae	13190	p13190-VIM	573	288771	809320	81	444	MF344563.gb	MF344563	MF344563	809320	0.05	5.31%
Klebsiella pneumoniae	FDAARGOS_447	unnamed1	573	207802	808970	12265	445	NZ_CP023952.1.gb	NZ_CP023952	NZ_CP023952.1	808970	0.05	5.31%
Enterobacter hormaechei	C45	pC45_001	158836	288659	808876	19069	446	NZ_CP042552.1.gb	NZ_CP042552	NZ_CP042552.1	808876	0.05	5.31%
Rhodococcus sp. S2-17	S2-17	pRB98	1990687	984201	808823	11263	447	NZ_CP021355.1.gb	NZ_CP021355	NZ_CP021355.1	808823	0.05	5.31%
Novosphingobium sp. P6W	P6W	pP6W1	1609758	720525	808580	14751	448	NZ_CP030354.1.gb	NZ_CP030354	NZ_CP030354.1	808580	0.05	5.31%
Marinobacter hydrocarbonoclasticus VT8	VT8	pMAQU02	351348	213290	807394	1042	449	NC_008739.1.gb	NC_008739	NC_008739.1	807394	0.05	5.30%
Klebsiella aerogenes	EA409	pEA409TEM24	548	167895	806810	104	450	MG764534.gb	MG764534	MG764534	806810	0.05	5.29%
Klebsiella pneumoniae		pRMH760	573	170613	806785	4314	451	NC_023898.1.gb	NC_023898	NC_023898.1	806785	0.05	5.29%
Klebsiella pneumoniae	13190	p13190-1	573	305423	806363	13123	452	NZ_CP026019.1.gb	NZ_CP026019	NZ_CP026019.1	806363	0.05	5.29%
Enterobacter sp. E76	E76	pE76_001	2596949	148871	806284	19043	453	NZ_CP042500.1.gb	NZ_CP042500	NZ_CP042500.1	806284	0.05	5.29%
Pseudomonas moraviensis R28-S	R28-S	pR28	1395516	81846	805129	8	454	AYMZ01000013.gb	AYMZ01000013	AYMZ01000013	805129	0.05	5.28%
Klebsiella pneumoniae subsp. pneumoniae	JNM8C2	pKJNM8C2.1	72407	304592	804922	14810	455	NZ_CP030858.1.gb	NZ_CP030858	NZ_CP030858.1	804922	0.05	5.28%
Mesorhizobium ciceri	CC1192	pMc1192	39645	648231	803929	8935	456	NZ_CP015063.1.gb	NZ_CP015063	NZ_CP015063.1	803929	0.05	5.28%
Enterobacter cloacae	Ecl4873	pEcl-Gr4873	550	153958	803898	30	457	KR559890.gb	KR559890	KR559890	803898	0.05	5.28%
Azospirillum brasilense Sp245	Sp245	AZOBR_p1	1064539	1766028	803798	2693	458	NC_016594.1.gb	NC_016594	NC_016594.1	803798	0.05	5.27%
Escherichia coli APEC O1	APEC O1	pAPEC-O1-R	405955	241387	803697	1178	459	NC_009838.1.gb	NC_009838	NC_009838.1	803697	0.05	5.27%
Providencia stuartii	BML2537	pBML2537	588	152754	803650	5838	460	NZ_AP022377.1.gb	NZ_AP022377	NZ_AP022377.1	803650	0.05	5.27%
Rhizobium sp. NXC24	NXC24	pRspNXC24c	2048897	2379650	803358	12458	461	NZ_CP024314.1.gb	NZ_CP024314	NZ_CP024314.1	803358	0.05	5.27%
Yersinia pseudotuberculosis	Yps.F1	pYps.F1	633	200660	803321	78	462	LT221036.gb	LT221036	LT221036	803321	0.05	5.27%
Rhizobium etli bv. mimosae str. Mim1	Mim1	pRetMIM1f	1328306	1082659	801952	4103	463	NC_021911.1.gb	NC_021911	NC_021911.1	801952	0.05	5.26%
Sphingomonas sp. YZ-8	YZ-8	unnamed1	2319844	849778	801554	15545	464	NZ_CP032828.1.gb	NZ_CP032828	NZ_CP032828.1	801554	0.05	5.26%
Klebsiella oxytoca KONIH1	KONIH1	pKOX-86d	1333852	193725	800768	6594	465	NZ_CP008790.1.gb	NZ_CP008790	NZ_CP008790.1	800768	0.05	5.25%
Microvirga ossetica	V5	unnamed2	1882682	977332	800601	9498	466	NZ_CP016619.1.gb	NZ_CP016619	NZ_CP016619.1	800601	0.05	5.25%
Azospirillum brasilense	Az39	AbAZ39_p3	192	686487	799432	6578	467	NZ_CP007796.1.gb	NZ_CP007796	NZ_CP007796.1	799432	0.05	5.25%
Ensifer adhaerens OV14	OV14	pOV14b	1416753	1614950	799200	6492	468	NZ_CP007239.1.gb	NZ_CP007239	NZ_CP007239.1	799200	0.05	5.24%
Klebsiella pneumoniae	KP14003	unnamed1	573	287790	797640	18847	469	NZ_CP041935.1.gb	NZ_CP041935	NZ_CP041935.1	797640	0.05	5.23%
Klebsiella aerogenes EA1509E	EA1509E	unnamed	935296	162202	797565	3801	470	NC_020180.1.gb	NC_020180	NC_020180.1	797565	0.05	5.23%
Providencia stuartii	FDAARGOS_645	unnamed1	588	186363	797262	19366	471	NZ_CP044075.1.gb	NZ_CP044075	NZ_CP044075.1	797262	0.05	5.23%
Klebsiella pneumoniae	AR_0109	unnamed2	573	310872	796553	15307	472	NZ_CP032208.1.gb	NZ_CP032208	NZ_CP032208.1	796553	0.05	5.23%
Alicycliphilus denitrificans BC	BC	pALIDE02	596153	78982	795796	2456	473	NC_014911.1.gb	NC_014911	NC_014911.1	795796	0.05	5.22%
Klebsiella pneumoniae	KP33	pKP3301	573	296750	794855	5676	474	NZ_AP018748.1.gb	NZ_AP018748	NZ_AP018748.1	794855	0.05	5.22%
Variovorax sp. WDL1		2	207745	816468	794853	21236	475	NZ_LR594690.1.gb	NZ_LR594690	NZ_LR594690.1	794853	0.05	5.22%
Enterobacter cloacae		p13E573-HI2	550	289837	793726	91	476	MF344581.gb	MF344581	MF344581	793726	0.05	5.21%
Aeromonas sp. ASNIH3	ASNIH3	pKPC-8e09	1636608	178635	793677	13224	477	NZ_CP026225.1.gb	NZ_CP026225	NZ_CP026225.1	793677	0.05	5.21%
Escherichia coli	L41-1	pL41-1-2	562	201021	792852	16211	478	NZ_CP034728.1.gb	NZ_CP034728	NZ_CP034728.1	792852	0.05	5.20%
Escherichia coli	L100	pL100-2	562	202387	792852	16225	479	NZ_CP034746.1.gb	NZ_CP034746	NZ_CP034746.1	792852	0.05	5.20%
Escherichia coli	VA292	pDGO100	562	217306	792120	45	480	KU997026.gb	KU997026	KU997026	792120	0.05	5.20%
Rhizobium leguminosarum	Vaf-108	unnamed1	384	1331126	791626	10079	481	NZ_CP018229.1.gb	NZ_CP018229	NZ_CP018229.1	791626	0.05	5.20%
Methylorubrum extorquens AM1	AM1	megaplasmid	272630	1261460	791181	1999	482	NC_012811.1.gb	NC_012811	NC_012811.1	791181	0.05	5.19%
Methylobacterium brachiatum	TX0642	unnamed1	269660	286730	791007	15692	483	NZ_CP033232.1.gb	NZ_CP033232	NZ_CP033232.1	791007	0.05	5.19%
Rhizobium etli 8C-3	8C-3	pRsp8C3c	538025	2253963	790774	9733	484	NZ_CP017244.1.gb	NZ_CP017244	NZ_CP017244.1	790774	0.05	5.19%
Alcaligenes faecalis	GZAF1	pGZAF1_VIM	511	124415	789228	63	485	KY623659.gb	KY623659	KY623659	789228	0.05	5.18%
Klebsiella pneumoniae	KPN11	pKPN11-1	573	330476	789031	21743	486	NZ_NCTN01000003.gb	NZ_NCTN01000	NZ_NCTN01000003	789031	0.05	5.18%
Phyllobacterium zundukense	Tri-48; RCAM 03910	unnamed2	1867719	586378	788905	9977	487	NZ_CP017943.1.gb	NZ_CP017943	NZ_CP017943.1	788905	0.05	5.18%
Azospirillum brasilense	MTCC4036	p2	192	1167812	787853	15376	488	NZ_CP032332.1.gb	NZ_CP032332	NZ_CP032332.1	787853	0.05	5.17%
Salmonella enterica subsp. enterica serovar Dublin	853	pSD_174	98360	173673	787600	3379	489	NC_019107.1.gb	NC_019107	NC_019107.1	787600	0.05	5.17%
Burkholderia sp. KK1	KK1	pkk4	1855726	568203	787307	9314	490	NZ_CP016005.1.gb	NZ_CP016005	NZ_CP016005.1	787307	0.05	5.17%
Aeromonas veronii	AVNIH1	pASP-a58	654	198307	786996	8796	491	NZ_CP014775.1.gb	NZ_CP014775	NZ_CP014775.1	786996	0.05	5.16%
Enterobacter hormaechei subsp. hoffmannii ECNIH3	ECNIH3	pENT-8a4	1333851	255013	786925	6631	492	NZ_CP008899.1.gb	NZ_CP008899	NZ_CP008899.1	786925	0.05	5.16%
Azospirillum brasilense	Sp 7	ABSP7_p1	192	1754523	786255	7999	493	NZ_CP012915.1.gb	NZ_CP012915	NZ_CP012915.1	786255	0.05	5.16%
Azospirillum brasilense	MTCC4038	p1	192	1757525	784804	15383	494	NZ_CP032340.1.gb	NZ_CP032340	NZ_CP032340.1	784804	0.05	5.15%
Providencia rettgeri	16pre36	p16Pre36-NDM	587	244116	784745	53	495	KX832927.gb	KX832927	KX832927	784745	0.05	5.15%
Sinorhizobium meliloti	M270	psymB	382	1694441	784621	11467	496	NZ_CP021814.1.gb	NZ_CP021814	NZ_CP021814.1	784621	0.05	5.15%
Novosphingobium sp. KA1	KA1	pCAR3	164608	254797	784452	964	497	NC_008308.1.gb	NC_008308	NC_008308.1	784452	0.05	5.15%
Enterobacter cloacae		2	550	150302	784160	21335	498	NZ_LS999207.1.gb	NZ_LS999207	NZ_LS999207.1	784160	0.05	5.15%
Sphingobium sp. YBL2	YBL2	2pYBL2-2	484429	227503	784009	7429	499	NZ_CP010956.1.gb	NZ_CP010956	NZ_CP010956.1	784009	0.05	5.15%
