# Supplementary figures and images for: Comparative analysis reveals the modular functional structure of conjugative megaplasmid pTTS12 of Pseudomonas putida S12: A paradigm for transferable traits, plasmid stability, and inheritance?
Source: Front Microbiol. 2022 Sep 23;13:1001472. doi: 10.3389/fmicb.2022.1001472 (PMC9537497; doi:10.3389/fmicb.2022.1001472)

Figure S1. A. CDS and B. DNA alignment of the top 500 plasmids with highest similarity score to pTTS12

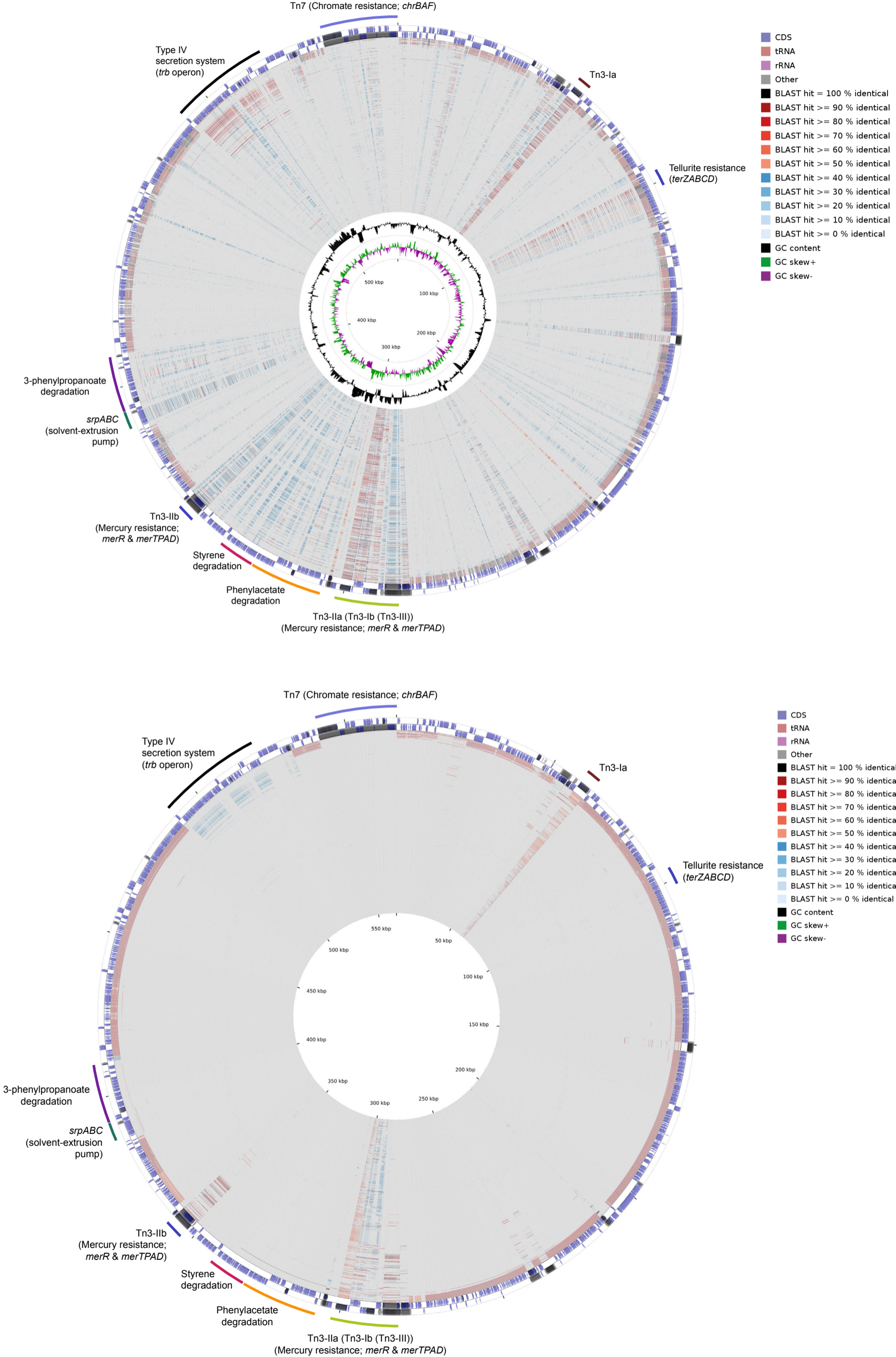

Supplement: Supplementary file 3 [file Image_1.PDF]
