# Supplementary material for: Comparative analysis reveals the modular functional structure of conjugative megaplasmid pTTS12 of Pseudomonas putida S12: A paradigm for transferable traits, plasmid stability, and inheritance?
Source: Front Microbiol. 2022 Sep 23;13:1001472. doi: 10.3389/fmicb.2022.1001472 (PMC9537497; doi:10.3389/fmicb.2022.1001472)
Supplement: Supplementary file 4 [file Image_2.PDF]

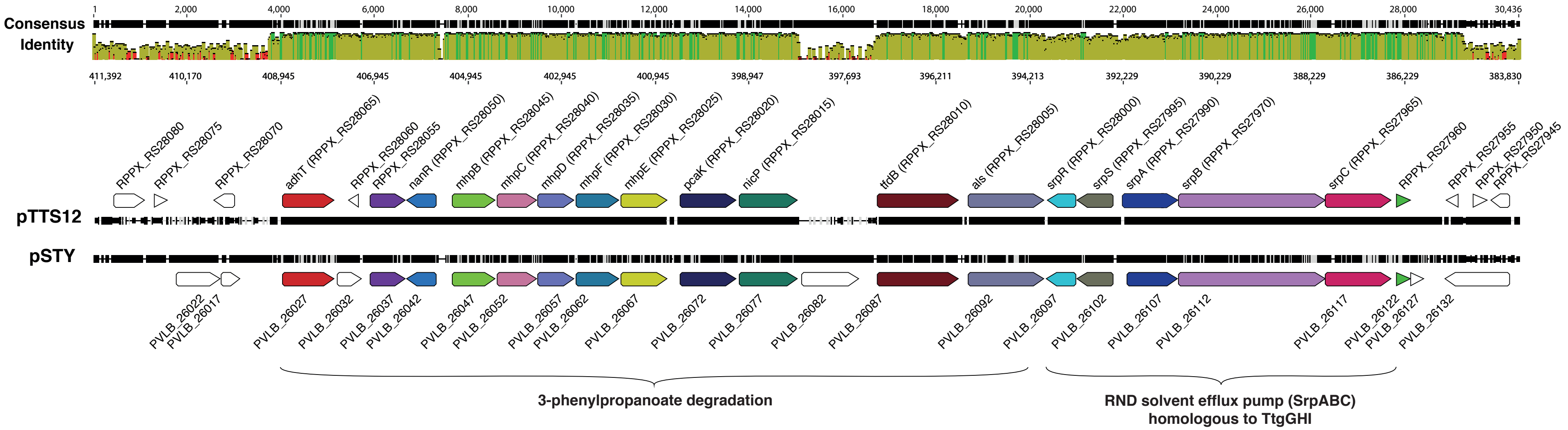

Figure S2. Comparison of the regions encoding 3-phenylpropionate degradation and solvent efflux pump between pTTS12 from *Pseudomonas putida* S12 and pSTY from *Pseudomonas taiwanensis* VLB120 reveals identical syntheny and high similarity.
