# Supplementary material for: Comparative analysis reveals the modular functional structure of conjugative megaplasmid pTTS12 of Pseudomonas putida S12: A paradigm for transferable traits, plasmid stability, and inheritance?
Source: Front Microbiol. 2022 Sep 23;13:1001472. doi: 10.3389/fmicb.2022.1001472 (PMC9537497; doi:10.3389/fmicb.2022.1001472)
Supplement: Supplementary file 5 [file Image_3.PDF]

Figure S3. Type IV secretion system encoded on *P. putida* S12 chromosome shares synteny with the I-type T4SS prototype of *L. pneumophila* Dot/Icm system and R64

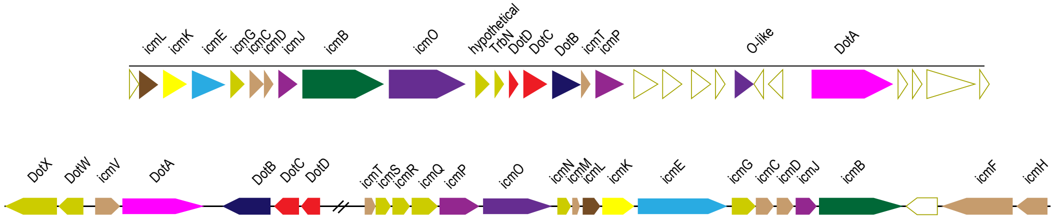

Protein	*C. burnetii* Ident./Sim.[a]	P. putida S12 Identity/similarity	P. putida S12 locustag	L. pneumophila Locustag	Length aminoacid	Length aminoacid 2	pTTS12 Identity (%)	pTTS12 similarity (%)
IcmT	47.1/63.2	15/33	RPPX_RS13055	lpl0483	73	87	15	33
IcmS	52.6/67.5	/	–	lpl0484	–			
IcmR	–	/	–	lpl0485	–			
IcmQb	22.6/34.0	/	–	lpl0486	–			
IcmP/DotM	36.5/50.3	18/38	RPPX_RS13060	lpl0487	377	437	18	38
IcmO/DotL	59.6/70.9	28/42	RPPX_RS13025	lpl0488	784	984	28	42
IcmN/DotK	24.8/32.7	/	–	lpl0489	–			
IcmM/DotJc	–	/	–	lpl0490	–			
IcmL/DotIc	35.8/48.5	18/41	RPPX_RS12985	lpl0491	213	255	18	41
IcmK/DotH	48.2/58.6	26/40	RPPX_RS12990	lpl0492	361	338	26	40
IcmE/DotG[d]	44.2/51.4	13/21	RPPX_RS12995	lpl0493	476	1049	13	21
IcmG/DotF	27.1/42.4	16/29	RPPX_RS13000	lpl0494	270	208	16	29
IcmC/DotE[e]	27.7/47.1	22/38	RPPX_RS13005	lpl0495	195	195	22	38
IcmD/DotP[e]	20.8/34.0	17/30	RPPX_RS13010	lpl0496	133	127	17	30
IcmJ/DotN	51.2/61.9	22/42	RPPX_RS13015	lpl0497	209	249	22	42
IcmB/DotO	62.2/73.7	34/54	RPPX_RS13020	lpl0498	1010	1021	34	54
IcmF[f]	23.1/34.4	/	–	lpl0500	–			
IcmH	27.7/37.6	/	–	lpl0501	–			
DotD	39.2/48.5	13/33	RPPX_RS13050	lpl2601	164	162	13	33
DotC	40.1/51.1	27/47	RPPX_RS13045	lpl2602	304	295	27	47
DotB	63.4/74.8	34/53	RPPX_RS13040	lpl2603	378	400	34	53
DotA	25.0/34.4	17/30	RPPX_RS13100	lpl2613	1048	995	17	30
IcmV	27.1/44.5	/	–	lpl2614	–			
IcmW	58.6/68.4	/	–	lpl2615	–			
IcmX	21.9/30.2	/	–	lpl2616	–			
