# Supplementary material for: Comparative analysis reveals the modular functional structure of conjugative megaplasmid pTTS12 of Pseudomonas putida S12: A paradigm for transferable traits, plasmid stability, and inheritance?
Source: Front Microbiol. 2022 Sep 23;13:1001472. doi: 10.3389/fmicb.2022.1001472 (PMC9537497; doi:10.3389/fmicb.2022.1001472)
Supplement: Supplementary file 6 [file Image_4.PDF]

Figure S4. The primary and auxiliary replication initiation proteins (RIP) of pTTS12 share synteny and homology with the RIPs of pOZ176

A. Primary RIP (RPPX_27060, annotated in purple)

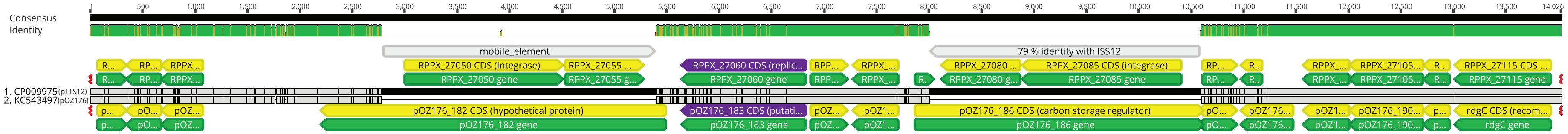

Name	Sequence Name	Minimum	Maximum	Length	Direction	Column1	description	% Pairwise	Seq.length
RPPX_27035 CDS (hypothetical protein)	CP009975_[170875-2752	70	348	279	forward	93	RPPX_27035 CDS (hypothetical protein) alignment	96.70%	92
RPPX_27040 CDS (hypothetical protein)	CP009975_[170875-2752	345	677	333	reverse	111	RPPX_27040 CDS (hypothetical protein) alignment	95.50%	110
RPPX_27045 CDS (hypothetical protein)	CP009975_[170875-2752	674	1072	399	reverse	133	RPPX_27045 CDS (hypothetical protein) alignment	93.90%	132
RPPX_27050 CDS (integrase)	CP009975_[170875-2752	2996	4510	1515	forward	505	disrupting (pOZ176_182 CDS (hypothetical protein))		
RPPX_27055 CDS (ATPase AAA)	CP009975_[170875-2752	4528	5283	756	forward	252	disrupting (pOZ176_182 CDS (hypothetical protein))		
RPPX_27060 CDS (replication protein)	CP009975_[170875-2752	5640	6827	1188	reverse	396	RPPX_27060 CDS (replication protein) alignment	99.70%	395
RPPX_27065 CDS (transcriptional regulator)	CP009975_[170875-2752	6866	7225	360	forward	120	RPPX_27065 CDS (transcriptional regulator) alignment	100.00%	119
RPPX_27070 CDS (hypothetical protein)	CP009975_[170875-2752	7273	7707	435	reverse	145	RPPX_27070 CDS (hypothetical protein) alignment	99.30%	144
RPPX_27080 CDS (ATPase AAA)	CP009975_[170875-2752	8114	8869	756	reverse	252	disrupting (pOZ176_186 CDS (carbon storage regulator))		
RPPX_27085 CDS (integrase)	CP009975_[170875-2752	8890	10404	1515	reverse	505	disrupting (pOZ176_186 CDS (carbon storage regulator))		
RPPX_27090 CDS (hypothetical protein)	CP009975_[170875-2752	10609	10944	336	forward	112	RPPX_27090 CDS (hypothetical protein) alignment	91.00%	111
RPPX_27095 CDS (hypothetical protein)	CP009975_[170875-2752	10969	11181	213	reverse	71	RPPX_27095 CDS (hypothetical protein) alignment	95.70%	168
RPPX_27100 CDS (hypothetical protein)	CP009975_[170875-2752	11563	12009	447	reverse	149	RPPX_27100 CDS (hypothetical protein) alignment	100.00%	148
RPPX_27105 CDS (hypothetical protein)	CP009975_[170875-2752	12006	12710	705	reverse	235	RPPX_27105 CDS (hypothetical protein) alignment	100.00%	234
RPPX_27110 CDS (hypothetical protein)	CP009975_[170875-2752	12717	12962	246	reverse	82	RPPX_27110 CDS (hypothetical protein) alignment	100.00%	81
RPPX_27115 CDS (recombinase RdgC)	CP009975_[170875-2752	13008	13928	921	reverse	307	RPPX_27115 CDS (recombinase RdgC) alignment	100.00%	306

B. Auxiliary RIP (RPPX_28775, annotated in purple)

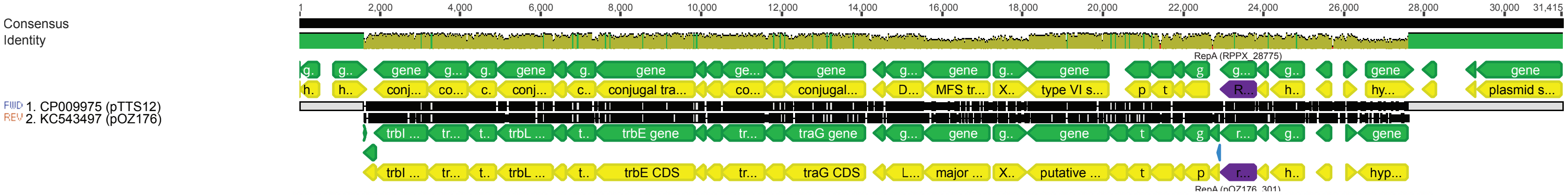

Name	Sequence Name	Minimum	Maximum	Length	Direction	locus_tag	Column1	% Pairwise identity	Seq.length
conjugal transfer protein TrbI CDS	CP009975 extraction - t4secretion region	1886	3178	1293	reverse	RPPX_28670	431	91.90%	430
conjugal transfer protein TrbG CDS	CP009975 extraction - t4secretion region	3181	4185	1005	reverse	RPPX_28675	335	92.80%	334
conjugal transfer protein TrbF CDS	CP009975 extraction - t4secretion region	4182	4886	705	reverse	RPPX_28680	235	90.20%	234
conjugal transfer protein TrbL CDS	CP009975 extraction - t4secretion region	4904	6283	1380	reverse	RPPX_28685	460	84.10%	459
lipoprotein CDS	CP009975 extraction - t4secretion region	6280	6594	315	reverse	RPPX_28690	105	66.70%	108
conjugal transfer protein TrbJ CDS	CP009975 extraction - t4secretion region	6607	7335	729	reverse	RPPX_28695	243	84.50%	245
conjugal transfer protein TrbE CDS	CP009975 extraction - t4secretion region	7332	9785	2454	reverse	RPPX_28700	818	89.50%	817
conjugal transfer protein TrbD CDS	CP009975 extraction - t4secretion region	9798	10067	270	reverse	RPPX_28705	90	88.90%	90
conjugal transfer protein TrbC CDS	CP009975 extraction - t4secretion region	10064	10450	387	reverse	RPPX_28710	129	89.80%	130
conjugal transfer protein TrbB CDS	CP009975 extraction - t4secretion region	10447	11517	1071	reverse	RPPX_28715	357	91.00%	356
CopG family transcriptional regulator CDS	CP009975 extraction - t4secretion region	11514	12002	489	reverse	RPPX_28720	163	88.60%	162
conjugal transfer protein TraG CDS	CP009975 extraction - t4secretion region	11999	14005	2007	reverse	RPPX_28725	669	90.90%	669
lipoprotein CDS	CP009975 extraction - t4secretion region	14241	14507	267	reverse	RPPX_28730	89	67.40%	89
D-alanyl-D-alanine endopeptidase CDS	CP009975 extraction - t4secretion region	14510	15454	945	reverse	RPPX_28735	315	79.90%	314
MFS transporter CDS	CP009975 extraction - t4secretion region	15469	16923	1455	reverse	RPPX_28740	485	27.20%	530
XRE family transcriptional regulator CDS	CP009975 extraction - t4secretion region	17022	17822	801	forward	RPPX_28745	267	50.40%	269
type VI secretion protein CDS	CP009975 extraction - t4secretion region	17836	19842	2007	reverse	RPPX_28750	669	90.70%	668
peptidase CDS	CP009975 extraction - t4secretion region	20291	20890	600	reverse	RPPX_28755	200	86.40%	199
transposase CDS	CP009975 extraction - t4secretion region	20887	21432	546	reverse	RPPX_28760	182	68.00%	181
parB partition protein CDS	CP009975 extraction - t4secretion region	21429	21716	288	reverse	RPPX_28765	96	83.90%	95
ATPase CDS	CP009975 extraction - t4secretion region	21713	22351	639	reverse	RPPX_28770	213	91.00%	212
RepA replication protein CDS	CP009975 extraction - t4secretion region	22641	23501	861	reverse	RPPX_28775	287	85.30%	286
transcriptional regulator CDS	CP009975 extraction - t4secretion region	23516	23797	282	reverse	RPPX_28780	94	83.00%	94
hypothetical protein CDS	CP009975 extraction - t4secretion region	23873	24679	807	reverse	RPPX_28785	269	79.90%	268
lipoprotein CDS	CP009975 extraction - t4secretion region	25001	25348	348	reverse	RPPX_28790	116	80.00%	116
XRE family transcriptional regulator CDS	CP009975 extraction - t4secretion region	25620	25916	297	forward	RPPX_28795	99	79.50%	98
hypothetical protein CDS	CP009975 extraction - t4secretion region	26148	27188	1041	forward	RPPX_28800	347	15.00%	397
