# Supplementary material for: Comparative analysis reveals the modular functional structure of conjugative megaplasmid pTTS12 of Pseudomonas putida S12: A paradigm for transferable traits, plasmid stability, and inheritance?
Source: Front Microbiol. 2022 Sep 23;13:1001472. doi: 10.3389/fmicb.2022.1001472 (PMC9537497; doi:10.3389/fmicb.2022.1001472)
Supplement: Supplementary file 7 [file Image_5.PDF]

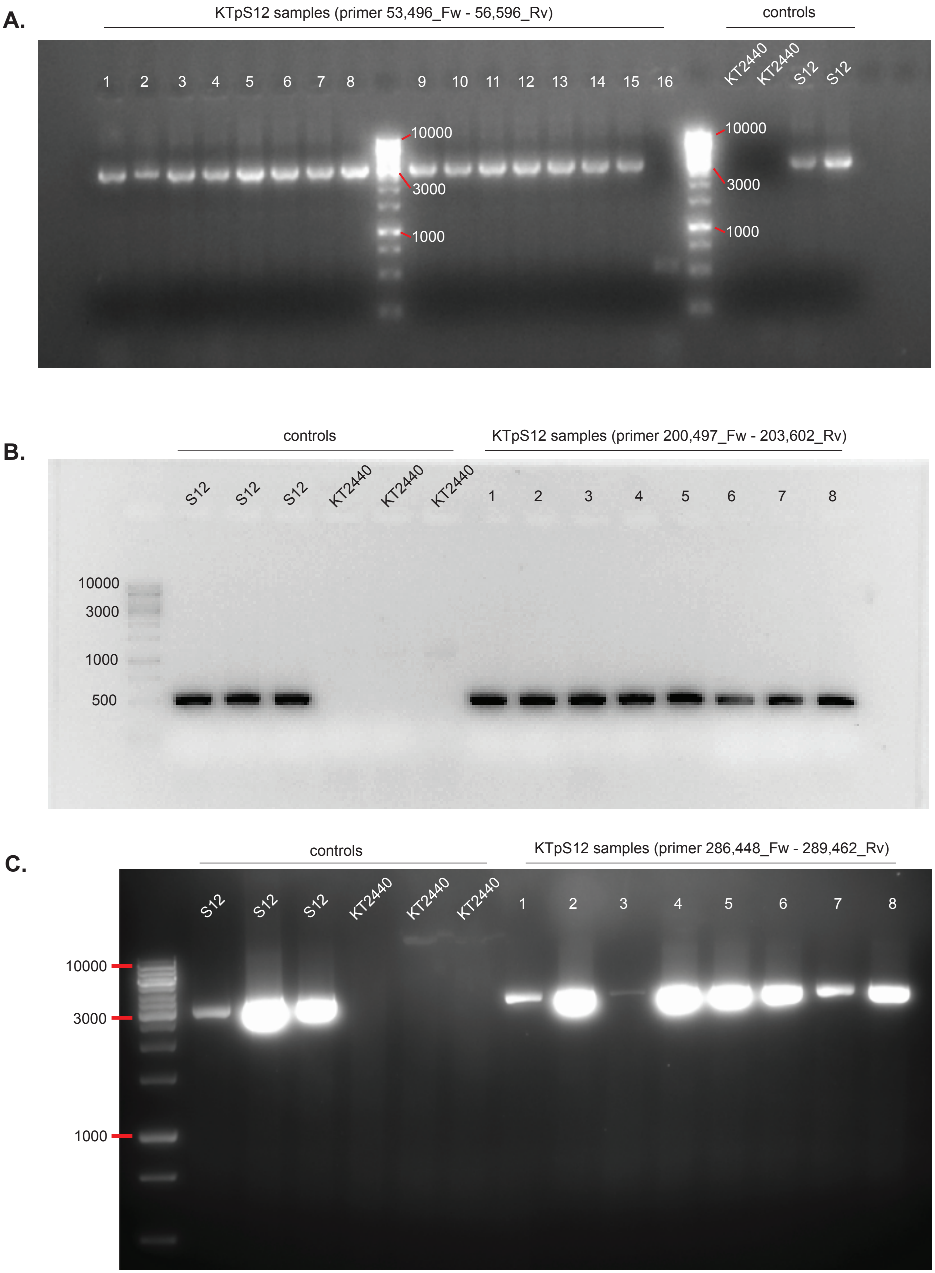


**Figure S5.** Electrophoresis gel image of PCR reactions to confirm correct pTTS12 transconjugant into KT2440 strain (strain KTp12) with A. primer pairs 53,496_Fw and 56,596_Rv (expected band size 3100 bp), B. primer pairs 200,497_Fw and 203,602_Rv (expected band size 511 bp), C. primer pairs 286,448_Fw and 289,462_Rv (expected band size 3015 bp. Six colonies (sample 1-6) were selected for further experiments to represent *P. putida* KTpS12 strain.
